# eQTL colocalization analysis highlights novel susceptibility genes in Autism Spectrum Disorders (ASD)

**DOI:** 10.1038/s41398-023-02621-0

**Published:** 2023-10-31

**Authors:** S. Dominguez-Alonso, A. Carracedo, C. Rodriguez-Fontenla

**Affiliations:** 1 Grupo de Medicina Xenómica, Center for Research in Molecular Medicine and Chronic Diseases (CiMUS), Universidad de Santiago de Compostela, Santiago de Compostela, Spain; 2 Grupo de Medicina Xenómica, Fundación Instituto de Investigación Sanitaria de Santiago de Compostela (FIDIS), Center for Research in Molecular Medicine and Chronic Diseases (CiMUS), Universidad de Santiago de Compostela, Santiago de Compostela, Spain

**Keywords:** Genomics, Psychiatric disorders

## Abstract

Autism Spectrum Disorders (ASD) are a group of neurodevelopmental disorders (NDDs) characterized by difficulties in social interaction and communication, repetitive behavior, and restricted interests. ASD has proven to have a strong genetic component. However, defining causal genes is still one of the main challenges in GWAS, since the vast majority (>90%) of detected signals lie within the non-coding genome. Expression quantitative trait locus (eQTL) colocalization analysis determines whether a specific variant is responsible for both a local eQTL and GWAS association and has helped leverage data and rendering gene discovery for a wide array of diseases. Here we further mine the largest ASD GWAS performed to date (18,381 cases and 27,969 controls) altogether with GWAS summary statistics from the main PGC studies (Schizophrenia, MD (Major Depression) and ADHD (Attention Deficit/Hyperactivity Disorder)), by using eQTpLot, a newly developed tool that illustrates the colocalization of GWAS and eQTL signals in a locus, and the enrichment of and correlation between the candidate gene eQTLs and trait-significant variants. This analysis points up 8 genes with a significant eQTL colocalization signal in ASD (*CRHR1, KANSL1, MANBA, MAPT, MMP12, NKX2-2, PTPRE* and *WNT3*) and one gene (*SRPK2*) with a marginally significant colocalization signal (*r* = 0.69, *p* < 1 × 10^−6^), and specifically highlights the potentially causal role of *MAPT* (*r* = 0.76, *p* < 1 × 10^−6^), *NKX2-2* (*r* = 0.71, *p*-value = 2.26^−02^) and *PTPRE* (*r* = 0.97, *p*-value = 2.63^−04^) when restricting the analysis to brain tissue.

## Introduction

Autism Spectrum Disorders (ASD) are a group of phenotypically and genetically heterogeneous neurodevelopmental disorders (NDDs) characterized by difficulties in social interaction and communication, repetitive behavior, and restricted interests (MIM209850). The population prevalence of ASD shows regional differences, but has been constantly increasing over the last decades [1]. In Europe, there is an average estimated prevalence of 12.2 per 1,000 children aged 7–9 years (“Autism Spectrum Disorders in Europe (ASDEU)”) [2]. ASD is 4 times more common in males than females and has an early onset, with an average age of diagnosis of 4 years [3].

On the basis of a large number of twin studies, ASD has proven to have a great genetic component (high heritability estimated around 80%) [4] with both common and rare variants contributing to its etiology [5, 6]. Thus far, hundreds of ASD loci have been identified, however, it was not until the latest ASD GWAS meta-analysis (18,381 cases and 27,969 controls) that first common risk variants were robustly associated with ASD by more than doubling the discovery sample size compared to previous GWAS [7]. However, defining implicated causal genes is still one of the main challenges in GWAS, since the vast majority (>90%) of detected signals lie within the non-coding genome so that it is unclear how these non-coding variants affect traits and diseases [8].

One possible explanation is that these GWAS risk loci exert their effect by its implication on gene expression in different tissues [9], so that providing evidence that the SNP is an eQTL (expression quantitative trail locus) can improve the ability to clarify the nature of the mechanism driving the associations by identifying whether or not the same variant is causal in both GWASs and eQTL studies. Few studies have examined eQTLs associated with specific genes implicated in ASD [10, 11] and a recent study has found over 30 eQTL-associated DNA variants with significantly different allele distributions between ASD-affected and control [12].

We herein further mine the largest ASD GWAS as well as previous gene-based association (GBA) [13] and transcriptome-wide association analysis (TWAS) [14] altogether with publicly available eQTL data on 49 different tissues by using eQTpLot [15], a newly developed tool that illustrates the colocalization of GWAS and eQTL signals in a locus, and the enrichment of and correlation between the candidate gene eQTLs and trait-significant variants.

Our analysis provides first insights into potential ASD candidate genes regulatory mechanisms (i.e., the contribution to variation in gene expression level). It highlights the relevance of leveraging eQTL data to narrow down potentially causal genes within previously reported disease-associated loci and provides robust confirmation of previous findings from GBA and TWAS studies.

## Material and methods

### Samples and collection

#### Discovery set

We conducted colocalization analyses using variant data for 46,350 autistic individuals from the latest ASD GWAS meta-analysis [7] across 49 tissues (Genotype-Tissue Expression (GTEx) Project v8) [16], in order to evaluate the extent of overlap between eQTL and GWAS signatures in ASD (colocalization, correlation between GWAS and eQTLs), and to refine the identification of potentially causal genes with a functional effect in genomic areas including multiple genes and associated SNPs.

Summary statistics from the latest ASD GWAS meta-analysis were obtained from the public repository available in the PGC website (http://www.med.unc.edu/pgc/results-and-downloads). The following dataset was employed: iPSYCH_PGC_ASD_Nov2017.gz which includes the meta-analysis of ASD by the Lundbeck Foundation Initiative for Integrative Psychiatric Research (iPSYCH) and the Psychiatric Genomics Consortium (PGC) released in November 2017 (18,381 cases and 27,969 controls) (Table 1). Additional information about the genotyping and QC methods employed are available at the PGC website.

**Table 1:** Characterization of cohorts included in the colocalization analysis.

Study name	Trait	Sample size		Design	Ancestry	Reference
		Cases	Controls			
iPSYCH	ASD	13,076	22,664	Case/Control	European	Grove et al. [7]
PGC	ASD	7387	11,359	Trios^a^	European	Grove et al. [7]
	MD	246,363	561,190	Case/Control	European	Howard et al. [36]
INDEPENDENT PGC	SZP	40,675	64,643	Case/Control	European	Pardiñas et al. [37]
CLOZUK + PGC	SZP	36,989	113,075	Case/Control	European	SWGPGC [38]
	ADHD	20,183	35,191	Case/Control	European	Demontis et al. [39]

*MD* Major Depression, *SZP* Schizophrenia, *ADHD* Attention Deficit/Hyperactivity Disorder, *SWGPGC* Schizophrenia Working Group of the Psychiatric Genomics Consortium.

^a^For trio designs, the control individuals are pseudocontrols generated from non-transmitted alleles.

eQTL data were obtained from GTEx v8 which examines 15,201 RNA-sequencing samples from 49 tissues of 838 postmortem donors. GTEx v8 data were obtained on February 3, 2022 from the GTEx Portal: GTEx_Analysis_v8_eQTL.tar (files *.signif_variant_gene_pairs.txt.gz).

#### Correlated neuropsychiatric diseases set

Given the close genetic and phenotypic relationships between ASD, SZP (Schizophrenia), MD (Major Depression) and ADHD (Attention Deficit/Hyperactivity Disorder) [7], follow-up analyses were performed using GWAS summary statistics from the main PGC studies that are available for download, with altogether 1 study for MD, 2 for Schizophrenia and 1 for ADHD (Table 1).

## eQTL analyses (visualization of colocalization between eQTL and gwas data)

### Selecting ASD associated genes from GBA and TWAS

For the following analyses, several genes were selected from previous ASD studies (Table 2, Supplementary Table 1). All analyses for each ASD associated gene use the previous summary statistics of the ASD GWAS meta-analysis as an input.

**Table 2:** ASD associated genes collected from bibliography and selected for eQTL colocalization study.

Genes	Genomic Region (Index variant)	Chr	Study	Reference
*KIZ, XRN2, NKX2–2, NKX2–4*	rs910805	20	GWAS	Grove et al. [7]
*C8orf74, SOX7, PINX1*	rs10099100	8		
*LOC102723661, PTBP2*	rs201910565	1		
*MACROD2*	rs71190156	20		
*KMT2E, SRPK2*	rs111931861	7		
*XRN2, KIZ*	rs910805	20	GBA (MAGMA)	Grove et al. [7]
*KCNN2*	rs13188074	5		
*KANSL1, MAPT*	rs142920272	17		
*MACROD2*	rs71190156	20		
*WNT3*	s146122400	17		
*MFHAS1*	rs17701675	8		
*XKR6*	rs6992403	8		
*MSRA*	rs7831557	8		
*CRHR1*	chr17:43965129	17		
*SOX7*	rs10099100	8		
*NTM*	rs529507	11		
*MMP12*	chr11:102751102	11		
*BLK*	rs2736342	8		
*XRN2, NKX2-4, KIZ, NKX2-2*	rs910805	20	GBA (PASCAL)	Alonso-Gonzalez et al. [13]
*KCNN2*	rs13188074	5		
*CRHR1-IT1, LOC644172*	rs142920272	17		
*C8orf74*	rs10099100	8		
*PINX1*	rs10099100	8	TWAS (UTMOST)	Rodríguez-Fontenla et al. [14]
*NKX2-2, NKX2-4*	rs910805	20		
*PTPRE*	rs227375	10		
*MANBA*	–	4		
*ERI1*	–	8		
*MITF*	–	3		
*CIPC*	–	14		

Genes in bold were associated for the first time in the referred study, underlined gene names were associated at the gastrointestinal level, thus will not be considered for following analyses.

*GBA* gene-based analysis, *GWAS* genome-wide association analysis, *TWAS* transcriptome-wide association analysis.

Twelve genes were selected through proximity to the 5 SNPs reaching genome-wide significance in the ASD GWAS from Grove and colleagues [7] (i.e., within 50 kb of the region spanned by all SNPs with *r*^2^ ≥ 0.6 to the index variant), who also performed a gene-based analysis (GBA) using MAGMA, allowing the identification of 15 associated genes. The majority of these genes were located near the genome-wide significant SNPs identified in the GWAS, but 7 genes are located in four additional genomic regions and a cluster of genes is harbored on chromosome 17 (*KANSL1, WNT3, MAPT and CRHR1*).

Another GBA further mining the same GWAS data but leveraging a novel GBA tool (PASCAL (Pathway scoring algorithm)) [13], highlighted additional genes which are here considered (*CRHR1-IT1* and *LOC644172*), although most of them were previously reported by MAGMA.

Lastly, Rodriguez-Fontenla et al. also integrated tissue-expression and genetic data from Grove ASD GWAS by performing transcriptome-wide association studies (TWAS) [14], demonstrating the association of 4 genes at the brain level (*CIPC, PINX1, NKX2-2* and *PTPRE*) and highlighting the association of *NKX2-2, MANBA, ERI1* and *MITF* at the gastrointestinal level. Due to lack of eQTL data to raise statistical power in the genes associated at the gastrointestinal level, they will not be taken into account in this study.

### eQTpLot: visualization of colocalization between EQTL and GWAS data

#### Input data and significance threshdolds

We ran eQTpLot using as input ASD GWAS summary statistics data from the Psychiatric Genomics Consortium (PGC) and 5 available studies aforementioned (*p*-values for the SNPs from GWAS analysis) and GTEx (expression data including *p*-values for the SNP from eQTL analysis) (see section above).

eQTpLot requires setting different arguments as the significance threshold for eQTL data and the significance threshold for GWAS data.

We defined a variant as an expression quantitative trait locus (eQTL) for the candidate gene if its gene expression *p*-value was ≤0.05 (significance threshold for eQTL, default option in eQTpLot); variants with *p*-value > 0.05 were excluded from the analysis. In order to identify the list of all significant variant-gene pairs in the GTex eQTL dataset, a genome-wide empirical *p*-value threshold, p_t_, was defined as the empirical *p*-value of the gene closest to the 0.05 FDR threshold. *p*_t_ was then used to calculate a nominal *p*-value threshold for each gene [17, 18]. Variants with a nominal *p*-value below the gene-level threshold were considered significant and included in the final list of variant-gene pairs.

The significance threshold for GWAS data was set to 5 × 10^−08^ for colocalization analysis (corresponds to the horizontal line in plots and GWAS significance thresholds for the eQTL enrichment plot, default option in eQTpLot) .

#### EQTL-GWAS colocalization plots

##### Multi and PAN Tissue analysis

In order to visualize the variants’ effect on candidate gene expression across all tissues, we performed Pan Tissue analysis by setting the argument tissue to ‘all’. In order to visualize its effect across multiple tissues contained in a specified list of tissues, we performed the Multi Tissue analysis focusing on brain tissue (“Brain_Amygdala”, “Brain_Anterior_cingulate_cortex_BA24”, “Brain_Caudate_basal_ganglia”, “Brain_Cerebellar_Hemisphere”, “Brain_Cerebellum”, “Brain_Cortex”, “Brain_Frontal_Cortex_BA9”, “Brain_Hippocampus”, “Brain_Hypothalamus”, “Brain_Nucleus_accumbens_basal_ganglia”, “Brain_Putamen_basal_ganglia”, “Brain_Spinal_cord_cervical_c-1” and “Brain_Substantia_nigra”).

The analyzed phenotype was ASD in all instances and the assembly of the human genome was hg19.

eQTL-GWAS colocalization plots were created defining a locus of interest (LOI) for each of the selected genes to include the target gene’s coordinates along with ± 1000 kb of flanking genome (specified with the argument range) (Supplementary Table 1, Supplementary Fig. 1), so that GWAS data (summary statistics) are filtered out to include only those variants which fall within the LOI. The mapping window was defined as 1 Mb on either side of the gene of interest, based on the consideration that only cis-eQTL (associated with expression of a gene within a 1 Mb distance [19]) were included in our analysis. The significance threshold was set to 5 × 10^−08^ for colocalization analysis (red line in colocalization plots).

##### Congruous and incongruous variants

An additional parameter, ‘congruence’, was set to TRUE in order to divide variants into two groups: congruous (variants with the same direction of effect on gene expression and the GWAS trait; *e.g*., a variant that is significantly associated with a target’s gene increased expression also results in an increase in the GWAS trait) and incongruous (variants with opposite effects on gene expression and the GWAS trait, *e.g.* a variant that is significantly associated with a target’s gene increased expression but results in a decrease in the GWAS trait).

##### EQTL enrichment plots

eQTL enrichment plots were generated for the selected LOI to test if there is a significant enrichment (*p*-value by Fisher’s exact test) for eQTLs among GWAS-significant variants, so that each variant in the chromosomal space defined inside the LOI is represented along the horizontal axis, with the inverse log of the *p*-value of association with the specified GWAS trait on the vertical axis.

##### P–P correlation plots

So as to analyze the correlation between GWAS and eQTL’s *p*-value, we plotted a best fit linear regression over the points, so that each of the variants within the LOI is plotted with −log10 (*p*-value eQTL) along the horizontal axis and −log10 (*p*-value GWAS) along the vertical axis. The Pearson correlation coefficient (*r*) and *p*-value of correlation (*p*) are also computed and displayed in the plot, differentiating between variants with congruous and incongruous direction of effects. Significant correlations are defined as having a *r* ≥ 0.7 and *p*-value of correlation ≤0.05.

### Gene expression, gene network and related human disease-associated genes analysis

#### Gene expression heatmap

GENE2FUNC, a tool of FUMA GWAS (https://fuma.ctglab.nl/) [20] was employed to carry out a gene expression heatmap. Those genes represented in Table 3 (genes yielding significance in the colocalization analysis) were used as an input. Expression values are TPM (Transcripts Per Million) for GTEx v8 and RPKM (Read Per Kilobase per Million). Heatmaps display the normalized expression value (zero mean normalization of log2 transformed expression), and darker red means higher relative expression of that gene in each label (tissue type), compared to a darker blue color in the same label.

#### Gene network analysis

HumanBase (https://hb.flatironinstitute.org/) was used to build a gene network for novel findings selected by the eQTL colocalization analysis (Table 3) and specific to brain tissue, capturing tissue-specific gene function (Tissue-Specific Gene Networks: GIANT). *MAPT, NKX2-2*, and *PTPRE* were selected as the input genes along with brain tissue in the 5 existing data types (co-expression, TF binding, interaction, GSEA microRNA targets, GSEA perturbations). The resultant network contains the subset of functionally related genes (Supplementary Table 4, *n* = 50), all of which were used to test for functional enrichment using genes annotated to Gene Ontology biological process terms. Within each cluster, representative processes and pathways that showed enrichment were identified and presented along with their corresponding *Q* values. The *Q* value of each term is calculated using one-sided Fisher’s exact tests and Benjamini–Hochberg corrections to correct for multiple tests (Table 4). We also carried out an enrichment analysis in DIsGeNET (https:// metascape.org), containing a large collections of genes and variants associated to human diseases (429,036 gene-disease associations (GDAs), linking 17,381 genes to 15,093 diseases and 72,870 variant-disease associations (VDAs), between 46,589 SNPs and 6356 diseases and phenotypes) [21].

## Results

For each gene with a significant association between GWAS and eQTL’s *p*-value (*r* > 0.7, *p*-value < 0.05), we further addressed colocalization in an additional set of GWAS studies for SZP, ADHD and MD because of previously reported highly significant genetic correlation [7, 22], so that we get more clues on the possible mechanisms underlying the colocalization signals.

From the total of 27 LOI analyzed here (Supplementary Table 2, Supplementary Figs. 2–44), we found 8 previously associated via GWAS/TWAS/GBA in which eQTL and GWAS signals colocalize (*r* > 0.7, *p*-value < 0.05): *CRHR1*, *KANSL1*, *MANBA*, *MAPT*, *MMP12*, *NKX2-2*, *PTPRE* and *WNT3* (Table 3, Supplementary Figs. 2–44). Although not reaching the positive correlation coeficient threshold of *r* > 0.7, *SRPK2* yields a marginal positive correlation with great statistical support (*r* = 0.69, *p* < 1 × 10^−6^). Moreover, we addressed whether the SNPs-eQTLs within these LOI colocalize in a different set of tissues, and found that *MAPT* (*r* = 0.76 congruent variants), *NKX2-2* (*r* = 0.71 congruent variants), and *PTPRE* (*r* = 0.70 congruent variants and *r* = 0.97 incongruent variants) exhibit the strongest correlation when restricting the eQTL analysis to brain tissue.

**Table 3:** Colocalization results for ASD genes yielding significance in at least one analysis.

	Pan Tissue analysis				Multi Tissue analysis (brain tissue)			
	Congruent		Incongruent		Congruent		Incongruent	
	*r*	*p*-value	*r*	*p*-value	*r*	*p*-value	*r*	*p*-value
*CRHR1*	0.96	<1 × 10^−6^	0.96	<1 × 10^−6^	−0.52	<1 × 10^−6^	−0.50	<1 × 10^−6^
*KANSL1*	0.82	<1 × 10^−6^	0.85	<1 × 10^−6^	−0.20	<1 × 10^−6^	−0.07	8.05^−03^
*MANBA*	0.62	<1 × 10^−6^	0.70	<1 × 10^−6^	0.02	7.61^−01^	0	9.87^−01^
*MAPT*	0.96	<1 × 10^−6^	0.96	<1 × 10^−6^	0.69	<1 × 10^−6^	**0.76**	**<1 × 10**^**−6**^
*MMP12*	0.72	2.14^−04^	0.56	1.01^−03^	*	*	*	*
*NKX2-2*	−0.36	2.25^−01^	0.71	2.26^−02^	−0.36	2.25^−01^	**0.71**	**2.26**^**−02**^
*PTPRE*	0.02	8.48^−01^	0.09	3.19^−01^	**0.70**	**1.59**^**−03**^	**0.97**	**2.63**^**−04**^
*SRPK2*	0.69**	<1 × 10^−6^	0.66	<1 × 10^−6^	*	*	*	*
*WNT3*	0.93	<1 × 10^−6^	0.94	<1 × 10^−6^	−0.15	6.82^−02^	−0.24	1.39^−02^

Significant results are underlined. Boldfaced gene names indicate a significant correlation signal when the analysis is restricted to brain tissue.

*There is no available data in the eQTL dataset that satisfies the significant eQTL *p*-value threshold for the gene.

**r marginally reaches the specified threshold.

When addressed in the follow-up analysis, all genes besides *NKX2-2* were confirmed to have a significant association between GWAS and eQTL’s *p*-value in correlated neuropsychiatric disorders, with *CRHR1, KANSL1, MAPT, PTPRE* and *WNT3* yielding a significant correlation when restricted to brain tissue (Supplementary Table 3).

### *CRHR1* (Corticotropin Releasing Hormone Receptor 1)

The available eQTL data is currently insufficient to draw definitive conclusions regarding the role of *CRHR1* in brain tissue. However, in the Pan Tissue analysis for ASD, this gene has exhibited significant colocalization of congruent/incongruent variants (r = 0.96, *p*-value < 1 × 10^−6^) (Supplementary Fig. 5). Similar trends were observed in both schizophrenia datasets (congruent *r* = 0.97, *p*-value < 1 × 10^−6^; incongruent *r* = 0.73, *p*-value < 1 × 10^−6^).

### *KANSL1* (KAT8 Regulatory NSL Complex Subunit 1)

*KANSL1* has shown positive correlation in the Pan Tissue analysis for ASD and congruent/incongruent variants (congruent r = 0.82, *p*-value < 1 × 10^−6^; incongruent *r* = 0.85, *p*-value < 1 × 10^−6^) (Supplementary Fig. 7). A significant correlation signal was observed specifically in brain tissue when assessed in SZP (incongruent *r* = 0.73, *p*-value = 3.04^−02^).

### *MANBA* (Mannosidase Beta A)

In the ASD Pan Tissue analysis, incongruent variants for *MANBA* proof significantly correlated (*r* = 0.70, *p*-value < 1 × 10^−6^) (Supplementary Figure 12), being this findings replicated in MD (congruent *r* = 0.81, *p*-value < 1 × 10^−6^; incongruent *r* = 0.84, *p*-value < 1 × 10^−6^).

### *MMP12* (Matrix Metallopeptidase 12)

*MMP12* has exhibited a positive correlation with congruent variants in ASD in the Pan Tissue analysis (*r* = 0.72, *p*-value = 2.14^−04^) (Supplementary Fig. 16), and the same direction of effects was observed in ADHD (r = 0.75, *p*-value = 8.94^−05^).

### *SRPK2* (SRSF Protein Kinase 2)

*SRPK2* barely meets the specified threshold set for the coefficient of correlation, but it has strong statistical support in the Pan Tissue analysis (r congruent = 0.69, *p*-value < 1 × 10^−6^) (Supplementary Figure 24). There is insufficient data to determine the correlation in brain tissue. However, when analyzed in correlated disorders, a similar effect was observed in ADHD (*r* congruent = 0.67, *p*-value < 1 × 10^−6^).

### *WNT3* (Wnt Family Member 3)

For *WNT3* the correlation signal in ASD comes from congruent/incongruent variants in the entire set of tissues (congruent *r* = 0.93, *p*-value < 1 × 10^−6^; incongruent *r* = 0.945, *p*-value < 1 × 10^−6^) (Supplementary Fig. 25). However, when colocalization was analyzed in other neuropsychiatric disorders, all of them resulted in a significant signal, with the strongest correlation observed in brain tissue for incongruous variants in one schizophrenia dataset (*r* = 0.91, *p*-value < 1 × 10^−6^).

### Significant correlations in the multi tissue analysis

Additionally, *NKX2-2* (NK2 Homeobox 2), *PTPRE* (Protein Tyrosine Phosphatase Receptor Type E) and *MAPT* (Microtubule-Associated Protein Tau) demonstrate a positive correlation with eQTL signals in brain tissue.

**Fig. 1:**
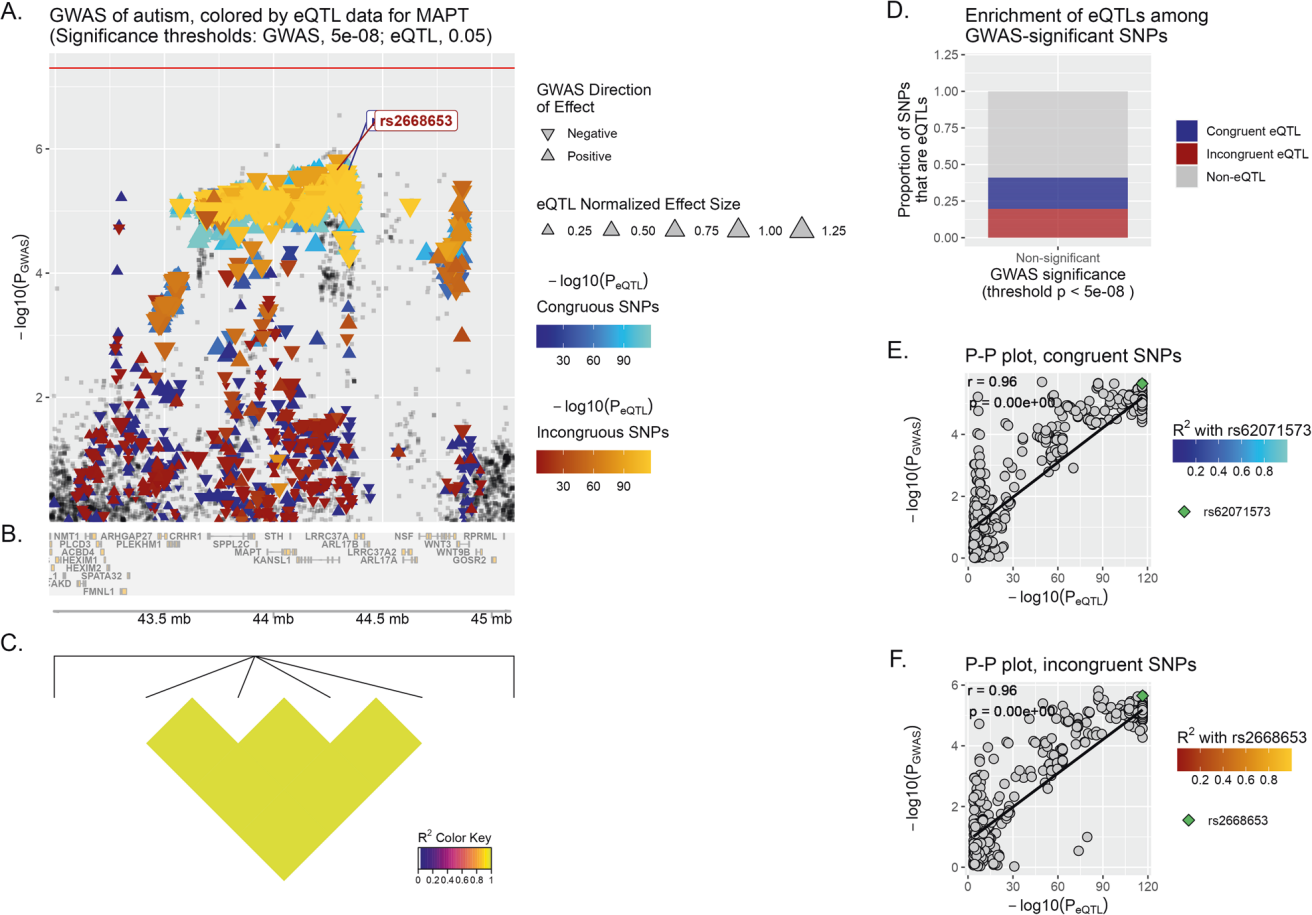
eQTpLot for Multi Tissue analysis (brain tissue) for *MAPT* in ASD. **A** Locus of interest, containing *MAPT*, with chromosomal space indicated along the horizontal axis. Within this plot, variants that lack eQTL data for the specified gene or do not meet the significance threshold are plotted as gray squares. On the opposite, variants that are determined to act as eQTLs for *MAPT* are plotted as colored triangles, with a color gradient corresponding to the inverse magnitude of its effect on gene expression or *p*-value. Congruous effects are plotted using a blue color scale, in contrast to variants having an incongruous effect, in red. The size of each triangle is proportional to the eQTL normalized effect size (NES), while the directionality of each triangle corresponds to the direction of effect of the variant on the GWAS trait. Horizontal red line marks the significance threshold in GWAS (*p*-value = 5 × 10^−08^). Finally, we show at the bottom of the plot a depiction of all genes’ genomic positions that fall inside the LOI (**B**) and a heatmap of LD information of all *MAPT* eQTL variants (**C**). **D** Enrichment of *MAPT* eQTLs among GWAS-significant/non-significant variants, while **E**, **F** depict the correlation between PGWAS and PeQTL for *MAPT* and ASD, with the computed Pearson correlation coefficient (*r*) and *p*-value (*p*) displayed on the plot. The lead variants, rs62071573 and rs2668653, are identified in each graph (by default the upper-right-most variant on the P–P plot), with all other variants plotted using a color scale corresponding to their squared coefficient of linkage correlation with this lead variant (if available). For reference, the same lead variants are also labeled in (**A**).

*MAPT* displays a significant correlation in the Multi Tissue analysis (brain) for incongruent variants *(r* = 0.76, *p* < 1 × 10^−6^) (Fig. 1), and follows the same trend in MD *(r* = 0.7, *p* < 1 × 10^−6^).

**Fig. 2:**
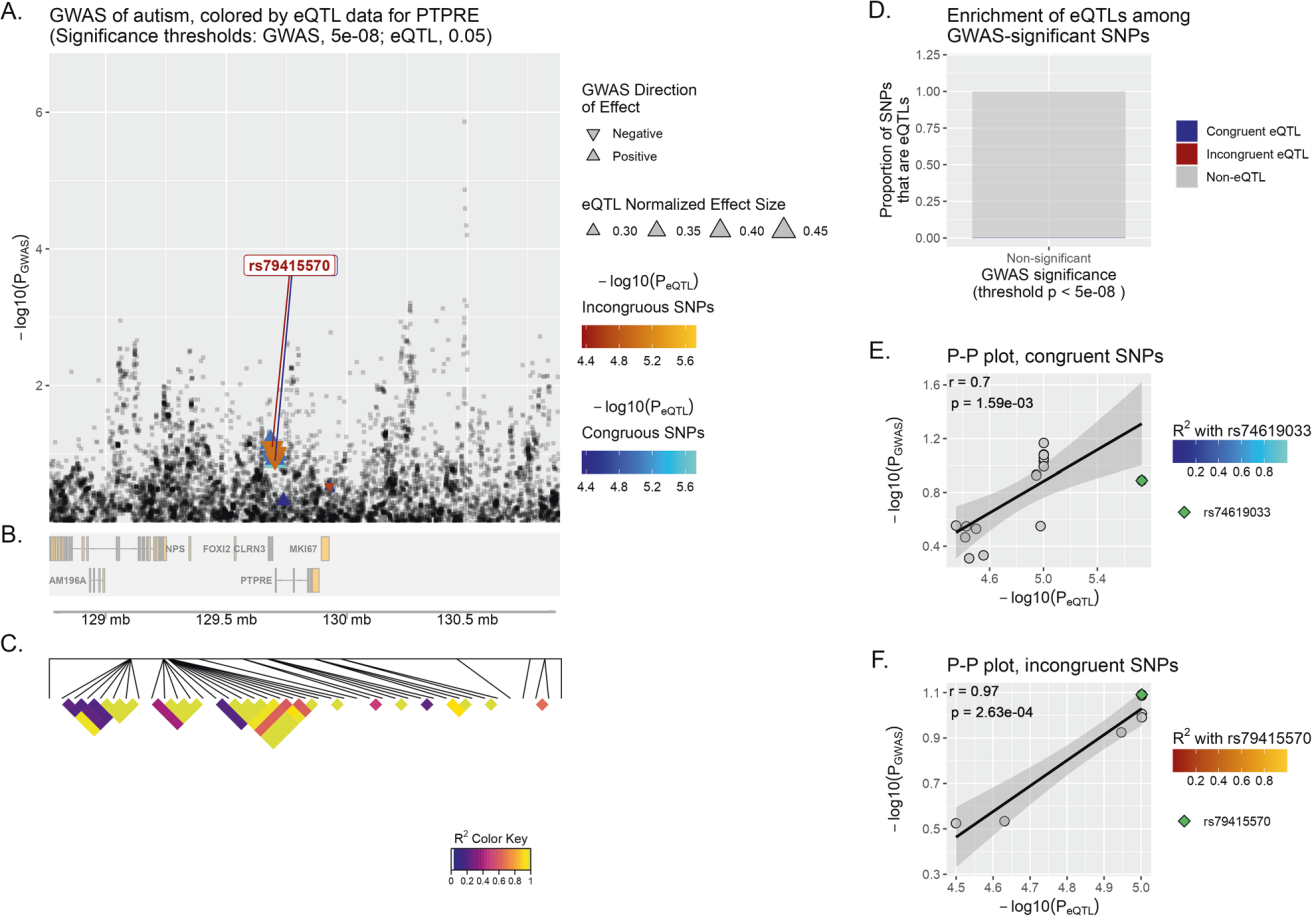
eQTpLot for Multi Tissue analysis (brain tissue) for *PTPRE* in ASD. See Fig. 1 for additional information about the figure legend.

*PTPRE* has yielded the strongest correlation in brain tissue overall (incongruent *r* = 0.97, *p*-value = 2.63^−04^) (Fig. 2). When examining this gene in the other set of neuropsychiatric diseases, significant correlations are observed in both ADHD and MD (incongruent *r* = 0.78, *p*-value = 8.46^−03^; incongruent *r* = 0.94, *p*-value = 6.15^−04^, respectively).

*NKX2-2* also demonstrates the highest correlation in brain tissue (incongruent *r* = 0.71, *p*-value = 2.26^−03^), but is the only gene that was not replicated in any other neurodevelopmental disorder included in the analysis (plot not shown).

### Gene expression, gene networks and related human disease-associated genes

**Fig. 3:**
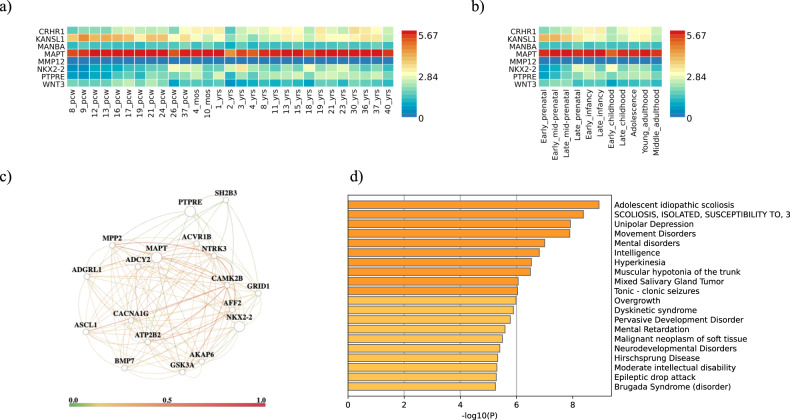
Expression analysis for genes showing eQTL colocalization and gene networks and associated human diseases analysis for genes achieving significance in brain tissue. **a** Gene expression heatmaps for *CRHR1*, *KANSL1*, *MANBA MAPT*, *MMP12, NKX2-2, PTPRE* and *WNT3* using BrainSpan 11 general developmental stages of brain samples and **b** BrainSpan 29 differentes ages of brain data. Genes are ordered by expression clusters and brain samples and ages by alphabetical order. MMP12 is not shown due to low expression levels in GTEx v8; **c** HumanBase gene network for *MAPT*, *NKX2-2* and *PTPRE* and functionally interactors in brain tissue (GIANT software); **d** Bar graph of enriched associated human diseases across input gene list, colored by *p*-values.

Expression analysis using BrainSpan RNA-seq data for the set of genes selected by eQTL analysis has shown overexpression of *MAPT* across every single developmental stage in comparison with the remaining studied genes (Fig. 3a, b). *MAPT, NKX2-2* and *PTPRE* and their interactors in brain tissue (Fig. 3c, Supplementary Table 4) show functional enrichment in processes associated with neurogenesis, neuron differentiation and nervous system functioning and development (Table 4). Moreover, these genes highlight different human diseases, primarily involved in movement and mental disorders (Fig. 3d).

**Table 4:** Functional enrichment for *MAPT, NKX2-2, PTPRE* and their interactors in brain tissue.

Name	Database	*q*-value
Peptidyl-threonine phosphorylation	Gene Ontology (BP)	0.014
Peptidyl-threonine modification	Gene Ontology (BP)	0.014
Neuron differentiation	Gene Ontology (BP)	0.014
Regulation of establishment of protein localization to mitochondrion	Gene Ontology (BP)	0.014
Generation of neurons	Gene Ontology (BP)	0.014
Neurogenesis	Gene Ontology (BP)	0.016
Regulation of neuron differentiation	Gene Ontology (BP)	0.028
Regulation of membrane potential	Gene Ontology (BP)	0.036
Regulation of neurogenesis	Gene Ontology (BP)	0.036
Negative regulation of neuron differentiation	Gene Ontology (BP)	0.036
Regulation of nervous system development	Gene Ontology (BP)	0.036
Negative regulation of transferase activity	Gene Ontology (BP)	0.036
Negative regulation of neurogenesis	Gene Ontology (BP)	0.039
Negative regulation of nervous system development	Gene Ontology (BP)	0.040
Establishment of protein localization to mitochondrion	Gene Ontology (BP)	0.044
Protein localization to mitochondrion	Gene Ontology (BP)	0.044
Positive regulation of transmembrane receptor protein serine/threonine kinase signaling pathway	Gene Ontology (BP)	0.044
Regulation of cell development	Gene Ontology (BP)	0.047

*BP*: *biological processes*.

## Discussion

One of the main difficulties when interpreting GWAS association signals is to identify the disease susceptibility genes underlying these associations, as the vast majority (>90%) fall within non-coding regions of the genome. Here, we show how approaches such as expression quantitative trait locus (eQTL) colocalization may help leverage GWAS data by connecting disease-associated loci with underlying mechanisms (*i.e*, the contribution to variation in gene expression level), being of service to determine which gene is most probably affected in specific tissues and to identify potential biological pathways.

In this analysis, we performed an eQTL colocalization analysis using the GTEx expression data and the largest ASD GWAS to date in order to identify potentially causal genes. Using this approach, we identified a total of 8 genes with a significant colocalizing eQTL (*CRHR1*, *KANSL1*, *MANBA*, *MAPT*, *MMP12*, *NKX2-2*, *PTPRE* and *WNT3*), with 3 of them (*MAPT, NKX2-2, PTPRE*) yielding a significant signal when restricted to brain tissue. Also, *SRPK2* achieved a high coefficient of correlation with great statistical support, barely reaching the defined threshold of *r* > 0.7.

*MAPT* was first associated with ASD in Grove et al. through a GBA [7]. It shows ubiquitous expression across all developmental stages and brain ages (Fig. 3a, b) and is differentially expressed in the nervous system, depending on the stage of neuronal maturation and neuron type. Mutations in the *MAPT* gene have been linked to various neurodegenerative conditions, including Alzheimer’s disease (AD), frontotemporal dementia, cortico-basal degeneration, and progressive supranuclear palsy [23]. Given the strong genetic correlation between Alzheimer’s disease and Schizophrenia [24], it is not surprising that we found a positive correlation for *MAPT*’s colocalization in the Schizophrenia dataset. Moreover, in another study, Huang et al. [25] further performed a TWAS of 7805 ASD proband-parent trios, which was subsequently replicated using 35,740 independent samples, and identified *MAPT* as a gene with a significant transcriptome-wide association with ASD.

In an additional TWAS, Rodriguez-Fontenla et al. [14] demonstrated for the first time the association of *PTPRE* at the brain level. It is worth noting that this specific gene demonstrates the highest correlation within brain tissue (*r* = 0.97) among all genes analyzed in this analysis. *PTPRE* encodes the protein tyrosine phosphatase, receptor type E, a member of the Protein tyrosine phosphatases (PTPs), which represent a superfamily of enzymes that play essential roles in normal development and physiology [26]. It has low human brain regional specificity and has not, do tate, been involved in any neurodevelopmental disorder. However, PTPs have been increasingly implicated in the control of neuronal synapse formation and function [27]. Moreover, another member of the PTP family, *PTPRD* (Protein Tyrosine Phosphatase Receptor Delta) has been genetically associated with neurodevelopmental disorders and psychiatric diseases, including ASD, ADHD and Schizophrenia [28]. These findings support the idea that *PTPRE* may be acting through similar molecular mechanisms leading to disorder onset, although further studies that characterize the role of *PTPRE* in the brain are required to elucidate its role in ASD.

Overall this shows how the utilization of both TWAS and eQTL colocalization methods provides a valuable approach to prioritize potentially causal genes underlying GWAS associations. This integrated approach enhances our ability to identify genes that are likely to play a role in the observed associations and provides further insights into the biological mechanisms involved in complex traits and diseases. By considering the expression patterns of genes and their genetic regulation, we can better understand the functional relevance of the identified genes and their potential contributions to the GWAS findings.

As a third relevant finding, we detected a positive correlation for *NKX2-2* in brain. *NKX2-2* was selected as being one of the nearest genes to the most genome-wide significant locus detected by Grove et al. (index variant rs910805, *p*-value = 2.04 × 10^−9^) [7]. This region spans three other genes (*KIZ*, *XRN2*, *NKX2–4*), but this analysis highlights *NKX2-2* through eQTL colocalization. Moreover, *NKX2-2* was also associated by Rodriguez-Fontenla et al. [14] at the brain level, but also at the gastrointestinal level. Our detected association was limited to brain tissue due to limited eQTL data for the gene in other tissues (eQTL variants for this gene only yielded significance in Cerebellum, although the previous TWAS study found a significant association for *NKX2-2* in brain nucleus accumbens basal ganglia and colon transverse). The TWAS performed by Huang et al. [25] also showed significant association in brain nucleus accumbens basal ganglia.

Although not reaching the correlation threshold for defining colocalization in this study (*r* > 0.7), we sought to consider *SRPK2*, not previously linked to ASD by any of the aforementioned analyses, nor in GBA or TWAS. However, this gene is one of the 5 genome-wide associated loci from Grove et al. (index variant rs111931861, *p*-value = 3.53 × 10^−8^), next to *KMT2E*, which has been already associated with a spectrum of NDDs, including ASD [29]. *SRPK2* triggers cell cycle progression in neurons and induces apoptosis and neurodegeneration, which are one of the main hallmarks in ASD [30, 31], suggesting that *SRPK2*’s functional characterization in ASD may be of high relevance. On the other hand, *SRPK2* has been widely linked to AD (Alzheimer’s disease) by phosphorylating tau, a neuronal microtubule-associated protein, and inducing its polymerization, thus leading to supression of tau-dependent microtubule polymerization, and inhibiting axonal elongation in neurons [30, 32, 33]. Depletion of *SRPK2* in dentate gyrus inhibits tau phosphorylation in APP/PS1 mouse (early-onset AD mouse model) and alleviates the impaired cognitive behaviors. This may be of special interest since recent studies have demonstrated that tau also enables autism-like behaviors and that even partial reduction of this protein prevents such behaviors and related neural abnormalities in independent mouse models [34].

Both of these results are quite relevant since: (i) rs910805 maps to *XRN2* (not previously implicated in ASD) but our analysis shows that associated variants at this locus are most probably altering *NKX2-2* expression; (ii) rs111931861 maps to *KMT2E* (already associated with ASD) but our analysis gives support to *SRPK2* (novel association). Overall, this highlights the relevance of performing complementary analysis to link GWAS variants with genes, and not just focusing on the ‘closest gene’ strategy [35].

Gene Ontology (GO) enrichment analysis of the 3 prioritized genes have shown enrichments in several brain-relevant ontologies for ASD etiology, such as neuron differentiation. Looking for enrichments in associated human diseases, we find an enrichment of these 3 genes, and their co-expressed interactors in brain tissue, in several conditions such as mental retardation and neurodevelopmental and psychiatric disorders among others, in agreement with the hypothesis that comorbidities/highly genetically correlated diseases that occur with ASD (*i.e*, epilepsy, gastrointestinal symptoms, depression, schizophrenia, etc.) are likely to share relevant associated genes and pathways.

In summary, we applied a colocalization approach using GTEx and identified two previously reported ASD genes (*NKX2-2* and *MAPT*, score 3 in SFARI rank) and two possible novel findings (*PTPRE* and *SRPK2)*. However, it is important to note three main limitations when exploring ASD common genetic variation: (i) early GWAS of ASD have failed to detect strong signals, possibly due to the presence of phenotypic heterogeneity across collections and the need of much larger sample sizes to achieve an adequate statistical power; (ii) numerous studies have found an important role of alternative splicing in ASD onset, so it may be possible that detected variants affect regulation at a different level than transcription regulation; (iii) due to intrinsic tissue heterogeneity, it may be needed to deepen our knowledge on eQTLs in specific cell types relevant to the disease, but also in different developmental stages, where eQTLs are not fully characterized.

Given all this limitations, our results show how eQTL analysis constitutes another way to identify the most probable gene (*i.e*. with the strongest support for causality) in genome-wide significant loci in a more biological relevant manner than just focusing on the closest gene to GWAS-significant associations, thus providing further evidence for functional validation. These results also highlighted the need of creating eQTL datasets in specific tissues/cell types/neurodevelopmental stages related to the disease in order to further identify statistically significant eQTLs. Moreover, ongoing GWAS projects with larger sample sizes, as the one presented here, will more than likely contribute to identify risk variants of modest effect not previously detected. Aditionally, the fact that most of the results are supported in datasets for the most genetically correlated diseases, suggests that understanding the regulatory mechanism at these loci may reveal the basis for pleiotropic effects across psychiatric disorders and that leveraging GWAS data from each disease might help to build a better comprehension of underlying common mechanisms for neurodevelopmental disorders.

### Supplementary information

Supplementary information
