# Supplementary material for: eQTL colocalization analysis highlights novel susceptibility genes in Autism Spectrum Disorders (ASD)
Source: Transl Psychiatry. 2023 Oct 31;13:336. doi: 10.1038/s41398-023-02621-0 (PMC10618232; doi:10.1038/s41398-023-02621-0)
Supplement: Supplementary file 1 — Supplementary information [file 41398_2023_2621_MOESM1_ESM.docx]

**eQTL colocalization analysis highlights novel susceptibility genes in Autism Spectrum Disorders (ASD)**

**Dominguez-Alonso S^1^, Carracedo A^1,2^, Rodriguez-Fontenla C^1*^.**

^1^Grupo de Medicina Xenómica, Center for Research in Molecular Medicine and Chronic Diseases (CiMUS), Universidad de Santiago de Compostela, Santiago de Compostela, Spain

^2^Grupo de Medicina Xenómica, Fundación Instituto de Investigación Sanitaria de Santiago de Compostela (FIDIS), Center for Research in Molecular Medicine and Chronic Diseases (CiMUS), Universidad de Santiago de Compostela, Santiago de Compostela, Spain

*** Correspondence:**

**Corresponding author:**

María Cristina Rodríguez Fontenla

Associate researcher

mariacristina.rodriguez.fontenla@usc.es

CiMUS (Center for Research in Molecular Medicine and Chronic Diseases)

Av Barcelona 31, 15706

Santiago de Compostela

A Coruña

Spain

^*^ These authors contributed equally to this work

CONTENTS

[PART I: SUPPLEMENTARY TABLES 4](#_Toc139277391)

[List of Supplementary Tables 4](#_Toc139277392)

[Supplementary Table 1 5](#_Toc139277393)

[Supplementary Table 2 6](#_Toc139277394)

[Supplementary Table 3 7](#_Toc139277395)

[Supplementary Table 4 8](#_Toc139277396)

[PART II: SUPPLEMENTARY FIGURES 9](#_Toc139277397)

[List of Supplementary Figures 9](#_Toc139277398)

[Regional Plots 11](#_Toc139277399)

[Supplementary Figure 1 12](#_Toc139277400)

[eQTL plots 12](#_Toc139277401)

[1. Pan Tissue Analysis 13](#_Toc139277402)

[Supplementary Figure 2 13](#_Toc139277403)

[Supplementary Figure 3 14](#_Toc139277404)

[Supplementary Figure 4 14](#_Toc139277405)

[Supplementary Figure 5 15](#_Toc139277406)

[Supplementary Figure 6 15](#_Toc139277407)

[Supplementary Figure 7 16](#_Toc139277408)

[Supplementary Figure 8 16](#_Toc139277409)

[Supplementary Figure 9 17](#_Toc139277410)

[Supplementary Figure 10 17](#_Toc139277411)

[Supplementary Figure 11 18](#_Toc139277412)

[Supplementary Figure 12 18](#_Toc139277413)

[Supplementary Figure 13 19](#_Toc139277414)

[Supplementary Figure 14 19](#_Toc139277415)

[Supplementary Figure 15 20](#_Toc139277416)

[Supplementary Figure 16 20](#_Toc139277417)

[Supplementary Figure 17 21](#_Toc139277418)

[Supplementary Figure 18 21](#_Toc139277419)

[Supplementary Figure 19 22](#_Toc139277420)

[Supplementary Figure 20 22](#_Toc139277421)

[Supplementary Figure 21 23](#_Toc139277422)

[Supplementary Figure 22 23](#_Toc139277423)

[Supplementary Figure 23 24](#_Toc139277424)

[Supplementary Figure 24 24](#_Toc139277425)

[Supplementary Figure 25 25](#_Toc139277426)

[Supplementary Figure 26 25](#_Toc139277427)

[Supplementary Figure 27 26](#_Toc139277428)

[2. Multi Tissue Analysis 27](#_Toc139277429)

[Supplementary Figure 28 27](#_Toc139277430)

[Supplementary Figure 29 27](#_Toc139277431)

[Supplementary Figure 30 28](#_Toc139277432)

[Supplementary Figure 31 28](#_Toc139277433)

[Supplementary Figure 32 29](#_Toc139277434)

[Supplementary Figure 33 29](#_Toc139277435)

[Supplementary Figure 34 30](#_Toc139277436)

[Supplementary Figure 35 30](#_Toc139277437)

[Supplementary Figure 36 31](#_Toc139277438)

[Supplementary Figure 37 31](#_Toc139277439)

[Supplementary Figure 38 32](#_Toc139277440)

[Supplementary Figure 39 32](#_Toc139277441)

[Supplementary Figure 41 33](#_Toc139277442)

[Supplementary Figure 42 34](#_Toc139277443)

[Supplementary Figure 43 34](#_Toc139277444)

[Supplementary Figure 44 35](#_Toc139277445)

[BIBLIOGRAPHY 36](#_Toc139277446)

# PART I: SUPPLEMENTARY TABLES

# List of Supplementary Tables

[1. Genomic coordinates of ASD associated genes. 5](#_Toc139277614)

[2. eQTL colocalization results for selected ASD genes. 6](#_Toc139277615)

[3. Colocalization results for potentially causal genes in correlated neuropsychiatric disorders. 7](#_Toc139277616)

[4. MAPT, NKX2-2 and PTPRE functionally related genes in brain tissue. 8](#_Toc139277617)

Supplementary Table 1**:** Genomic coordinates of ASD associated genes collected from bibliography and selected for eQTL colocalization study.

| **Genes** | **CHR** | **Start (BP)** | **Stop (BP)** |
| --- | --- | --- | --- |
| *BLK* | 8 | 11351499 | 11422113 |
| *C8orf74* | 8 | 10530146 | 10558103 |
| *CIPC* | 14 | 77564577 | 77583630 |
| *CRHR1* | 17 | 43861645 | 43913194 |
| *ERI1* | 8 | 8749734 | 8957772 |
| *KANSL1* | 17 | 44104700 | 44302740 |
| *KCNN2* | 5 | 113391702 | 113832197 |
| *KIZ* | 20 | 21106615 | 21227260 |
| *KMT2E* | 7 | 104581395 | 104754532 |
| *MACROD2* | 20 | 13976145 | 16033842 |
| *MANBA* | 4 | 103552642 | 103682151 |
| *MAPT* | 17 | 43971747 | 44105700 |
| *MFHAS1* | 8 | 8640863 | 8751131 |
| *MITF* | 3 | 69788585 | 70017488 |
| *MMP12* | 11 | 102733459 | 102745764 |
| *MSRA* | 8 | 9911733 | 10286401 |
| *NKX2-2* | 20 | 21491647 | 21494664 |
| *NKX2-4* | 20 | 21376004 | 21378047 |
| *NTM* | 11 | 131240202 | 132206716 |
| PINX1 | 8 | 10622470 | 10697409 |
| *PTBP2* | 1 | 97187160 | 97280605 |
| *PTPRE* | 10 | 129705316 | 129884119 |
| *SOX7* | 8 | 10581277 | 10588084 |
| *SRPK2* | 7 | 104751154 | 105039810 |
| *WNT3* | 17 | 44839871 | 44896126 |
| *XKR6* | 8 | 10751273 | 11058919 |
| *XRN2* | 20 | 21283921 | 21370463 |

Supplementary Table 2**:** eQTL colocalization analysis results for the 27 ASD associated genes collected from bibliography. Significant results (r>0.7, p-value <0.05) are underlined. Boldfaced gene names indicate a significant correlation signal when the analysis is restricted to brain tissue.

| **Gene** | **Pan Tissue Analysis** | | | | **Multi Tissue Analysis (Brain Tissue)** | | | | |
| --- | --- | --- | --- | --- | --- | --- | --- | --- | --- |
|  | **Congruent** | | **Incongruent** | | **Congruent** | | | **Incongruent** | |
|  | r | p-value | r | p-value | r | p-value | r | | p-value |
| *BLK* | 0,46 | <1x10^-6^ | 0,48 | <1x10^-6^ | 0,22 | 7,38^-01^ | 0,64 | | 2,49^-01^ |
| *C8orf74* | 0,39 | 5,09^-02^ | 0,43 | 3,29^-03^ | ** | ** | ** | | ** |
| *CIPC* | 0,48 | 2.35^-06^ | 0,41 | 1,12^-04^ | 0,69 | 1.43^-06^ | 0,41 | | 5,07^-03^ |
| *CRHR1* | 0,96 | <1x10^-6^ | 0,956 | <1x10^-6^ | -0,52 | <1x10^-6^ | -0,5 | | <1x10^-6^ |
| *ERI1* | 0,585 | <1x10^-6^ | 0,619 | <1x10^-6^ | -0.21 | 1.40^-02^ | -0,25 | | 3,93^-03^ |
| *KANSL1* | 0,82 | <1x10^-6^ | 0,85 | <1x10^-6^ | -0,2 | <1x10^-6^ | -0,074 | | 8,05^-03^ |
| *KCNN2* | -0,08 | 2,33^-01^ | -0,15 | 1,96^-02^ | * | * | * | | * |
| *KIZ* | 0,47 | <1x10^-6^ | 0,53 | <1x10^-6^ | 0,22 | 1,49^-01^ | -0,38 | | 9,94^-03^ |
| *KMT2E* | 0,27 | 8.58^-03^ | 0,12 | 2.85^-01^ | * | * | * | | * |
| *MACROD2* | -0,02 | 8,46^-01^ | -0,01 | 9,39^-01^ | * | * | * | | * |
| *MANBA* | 0,617 | <1x10^-6^ | 0,7 | <1x10^-6^ | 0,02 | 7,61^-01^ | 0,00 | | 9,87^-01^ |
| ***MAPT*** | 0,96 | 0 | 0,96 | 0 | 0,69 | <1x10^-6^ | 0,76 | | <1x10^-6^ |
| *MFHAS1* | 0,2 | <1x10^-6^ | 0,464 | <1x10^-6^ | * | * | * | | * |
| *MITF* | 0,462 | <1x10^-6^ | -0,385 | <1x10^-6^ | *** | *** | *** | | *** |
| *MMP12* | 0,72 | 2,14^-04^ | 0,56 | 1,01^-03^ | ** | ** | ** | | ** |
| *MSRA* | 0,27 | <1x10^-6^ | 0,23 | <1x10^-6^ | 0.01 | 9.18^-01^ | 0.2 | | 1.47^-02^ |
| ***NKX2-2*** | -0,361 | 2,25^-01^ | 0,71 | 2,26^-02^ | -0,36 | 2,25^-01^ | 0,71 | | 2,26^-02^ |
| *NKX2-4* | ** | ** | ** | ** | ** | ** | ** | | ** |
| *NTM* | 0,3 | <1x10^-6^ | 0,37 | <1x10^-6^ | 0,12 | 3,96^-01^ | 0,06 | | 7,82^-01^ |
| *PINX1* | 0.42 | 9.56^-05^ | 0.57 | <1x10^-6^ | 0.26 | 8.07^-02^ | 0.48 | | 6.49^-04^ |
| *PTBP2* | 0,35 | 3,21^-17^ | -0,396 | 1,35^-20^ | 0,17 | 1,26^-01^ | 0,14 | | 2,24^-01^ |
| ***PTPRE*** | 0,02 | 8,48^-01^ | 0,09 | 3,19^-01^ | 0,70 | 1,59^-03^ | 0,97 | | 2,63^-04^ |
| *SOX7* | -0.35 | <1x10^-6^ | -0.37 | <1x10^-6^ | 0.05 | 6.45^-01^ | -0.03 | | 7.88^-01^ |
| *SRPK2* | 0,69 | <1x10^-6^ | 0,66 | <1x10^-6^ | * | * | * | | * |
| *WNT3* | 0,93 | <1x10^-6^ | 0,945 | <1x10^-6^ | -0,15 | 6,82^-02^ | -0,24 | | 1,39^-02^ |
| *XKR6* | -0,18 | 3,12^-02^ | -0,13 | 1,34^-01^ | *** | *** | *** | | *** |
| *XRN2* | 0,38 | <1x10^-6^ | 0,44 | <1x10^-6^ | 0,35 | 3,19^-01^ | -0,23 | | 5,57^-01^ |

*Not enough data to generate P-P plot for Congruent/Incongruent eQTLs; **No data is available for the gene meeting the eQTL p-value significance threshold; *** Not enough finite observations

Supplementary Table 3**:** Colocalization results for potentially causal genes selected from the ASD eQTL colocalization analysis in correlated neuropsychiatric disorders. MD, major depression; SZP schizophrenia; ADHD, attention deficit/hyperactivity disorder; SWGPGC, Schizophrenia Working Group of the Psychiatric Genomics Consortium.

| **Gene** | **All tissues** | | | | | | **Brain** | | | | | | **Trait** | **Reference** | |
| --- | --- | --- | --- | --- | --- | --- | --- | --- | --- | --- | --- | --- | --- | --- | --- |
|  | **Congruent** | | | **Incongruent** | | | | **Congruent** | | | **Incongruent** | |  |  |  |
|  | r | p-value | r | | p-value | r | | | p-value | r | | p-value |  |  |  |
| ***CRHR1*** | 0.82  0.81 | 0  6.12^-50^ | 0.97  0.82 | | 4.53^-06^  6.71^-45^ | 0.68  0.87 | | | 8.73^-315^  3.47^-59^ | 0.97  0.73 | | 1.5^-06^  3.47^-28^ | SZP  SZP | | Pardiñas *et al.*, 2018^1^  SWGPGC, 2014^2^ |
| ***KANSL1*** | 0.85  0.8 | 0  1.13^-85^ | 0.14  0.84 | | 2.92^-02^  9.37^-100^ | 0.61  0.35 | | | 1.6^-242^  6.78^-11^ | 0.18  0.73 | | 1.28^-02^  6.19^-52^ | SZP  SZP | | Pardiñas *et al.*, 2018^1^  SWGPGC, 2014^2^ |
| *MANBA* | 0.81 | 1.92^-128^ | 0.84 | | 1.72^-154^ | 0.29 | | | 1.78^-05^ | 0.3 | | 2.02^-05^ | MD | | Demontis *et al.*, 2019^3^ |
| ***MAPT*** | 0.87  0.86  0.9 | 0  1.13^-95^  0 | 0.36  0.87  0.89 | | 5.29^-03^  1.45^-87^  0 | 0.17  0.57  0.61 | | | 1.76^-01^  2.46^-14^  1.76^-118^ | 0.45  0.49  0.7 | | 6.82^-112^  2.09^-11^  5.8^-173^ | SZP  SZP  MD | | Pardiñas *et al.*, 2018^1^  SWGPGC, 2014^2^  Demontis *et al.*, 2019^3^ |
| *MMP12* | 0.75 | 8.94^-05^ | 0.63 | | 1.16^-04^ | *** | | | *** | *** | | *** | ADHD | | Howard *et al.,* 2019^4^ |
| ***PTPRE*** | 0.2  -0.04 | 4.57^-02^  7.09^-01^ | 0.15  0.09 | | 1.24^-01^  3.64^-01^ | 0.34  0.37 | | | 2.87^-01^  1.53^-01^ | 0.78  0.94 | | 8.46^-03^  6.15^-04^ | ADHD  MD | | Howard *et al.,* 2019^4^  Demontis *et al.*, 2019^3^ |
| ***WNT3*** | -0.62  0.86  0.9  0.79 | 2.84^-26^  0  5.33^-81^  4.64^-216^ | -0.59  0.41  0.93  0.81 | | 5.54^-20^  3.62^-06^  3.13^-98^  1.15^-217^ | -0.7  0.68  0.88  0.71 | | | 1.45^-31^  7.11^-218^  3.01^-61^  8.23^-137^ | -0.68  0.21  0.91  0.82 | | 7.34^-24^  4.37^-02^  1.12^-74^  5.73^-212^ | ADHD  SZP  SZP  MD | | Howard *et al.,* 2019^4^  Pardiñas *et al.*, 2018^1^  SWGPGC, 2014^2^  Demontis *et al.*, 2019^3^ |

*** *No data is available for the gene meeting the eQTL p-value significance threshold. Significant results are underlined. Boldfaced gene names indicate a significant correlation signal when the analysis is restricted to brain tissue.*

Supplementary Table 4**:** *MAPT, NKX2-2* and *PTPRE* functionally related genes in brain tissue.

| **XSymbol** | **Name** |
| --- | --- |
| ***SH2B3*** | SH2B adaptor protein 3 |
| ***RAB31*** | RAB31 |
| ***ABR*** | ABR |
| ***ETS2*** | ETS proto-oncogene 2 |
| ***DUSP6*** | dual specificity phosphatase 6 |
| ***HBEGF*** | heparin binding EGF like growth factor |
| ***CSGALNACT2*** | chondroitin sulfate N-acetylgalactosaminyltransferase 2 |
| ***NUMB*** | NUMB |
| ***CCR1*** | C-C motif chemokine receptor 1 |
| ***ATP2B1*** | ATPase plasma membrane Ca2+ transporting 1 |
| ***LTBP2*** | latent transforming growth factor beta binding protein 2 |
| ***ARL4C*** | ADP ribosylation factor like GTPase 4C |
| ***IDS*** | iduronate 2-sulfatase |
| ***TRIM8*** | tripartite motif containing 8 |
| ***GLIPR1*** | GLI pathogenesis related 1 |
| ***HIVEP2*** | human immunodeficiency virus type I enhancer binding protein 2 |
| ***ARNTL*** | aryl hydrocarbon receptor nuclear translocator like |
| ***TNFAIP2*** | TNF alpha induced protein 2 |
| ***DOCK4*** | dedicator of cytokinesis 4 |
| ***VCAN*** | versican |
| ***IRS2*** | insulin receptor substrate 2 |
| ***THEMIS2*** | thymocyte selection associated family member 2 |
| ***EPHB2*** | EPH receptor B2 |
| ***MTMR6*** | myotubularin related protein 6 |
| ***SNN*** | stannin |
| ***ITGAM*** | integrin subunit alpha M |
| ***LAPTM5*** | lysosomal protein transmembrane 5 |
| ***LRRFIP1*** | LRR binding FLII interacting protein 1 |
| ***ATP6V1A*** | ATPase H+ transporting V1 subunit A |
| ***TACC1*** | transforming acidic coiled-coil containing protein 1 |
| ***KLF4*** | Kruppel like factor 4 |
| ***CXCL1*** | C-X-C motif chemokine ligand 1 |
| ***LCP2*** | lymphocyte cytosolic protein 2 |
| ***FAM49A*** | family with sequence similarity 49 member A |
| ***TRIM33*** | tripartite motif containing 33 |
| ***CREM*** | cAMP responsive element modulator |
| ***IGF2R*** | insulin like growth factor 2 receptor |
| ***ITPR1*** | inositol 1 |
| ***IL1R1*** | interleukin 1 receptor type 1 |
| ***ITSN2*** | intersectin 2 |
| ***PCDH7*** | protocadherin 7 |
| ***PELI1*** | pellino E3 ubiquitin protein ligase 1 |
| ***ABCA1*** | ATP binding cassette subfamily A member 1 |
| ***PTEN*** | phosphatase and tensin homolog |
| ***KIF1B*** | kinesin family member 1B |
| ***IL1B*** | interleukin 1 beta |
| ***IL13RA1*** | interleukin 13 receptor subunit alpha 1 |
| ***SPRED1*** | sprouty related EVH1 domain containing 1 |
| ***SIRPA*** | signal regulatory protein alpha |
| ***MICAL2*** | microtubule associated monooxygenase |

# PART II: SUPPLEMENTARY FIGURES

# List of Supplementary Figures

[Regional Plots 11](#_Toc139278594)

[1. Regional association plots for ASD genes selected by eQTL colocalization analysis. 12](#_Toc139278595)

[eQTL plots 12](#_Toc139278596)

[2. eQTpLot for Pan Tissue analysis for BLK in ASD. 13](#_Toc139278597)

[3. eQTpLot for Pan Tissue analysis for C8orf74 in ASD. 14](#_Toc139278598)

[4. eQTpLot for Pan Tissue analysis for CIPC in ASD. 14](#_Toc139278599)

[5. eQTpLot for Pan Tissue analysis for CRHR1 in ASD. 15](#_Toc139278600)

[6. eQTpLot for Pan Tissue analysis for ERI1 in ASD. 15](#_Toc139278601)

[7. eQTpLot for Pan Tissue analysis for KANSL1 in ASD. 16](#_Toc139278602)

[8. eQTpLot for Pan Tissue analysis for KCNN2 in ASD. 16](#_Toc139278603)

[9. eQTpLot for Pan Tissue analysis for KIZ in ASD 17](#_Toc139278604)

[10. eQTpLot for Pan Tissue analysis for KMT2E in ASD. 17](#_Toc139278605)

[11. eQTpLot for Pan Tissue analysis for MACROD2 in ASD. 18](#_Toc139278606)

[12. eQTpLot for Pan Tissue analysis for MANBA in ASD. 18](#_Toc139278607)

[13. eQTpLot for Pan Tissue analysis for MAPT in ASD. 19](#_Toc139278608)

[14. eQTpLot for Pan Tissue analysis for MFHAS1 in ASD. 19](#_Toc139278609)

[15. eQTpLot for Pan Tissue analysis for MITF in ASD. 20](#_Toc139278610)

[16. eQTpLot for Pan Tissue analysis for MMP12 in ASD. 20](#_Toc139278611)

[17. eQTpLot for Pan Tissue analysis for MSRA in ASD. 21](#_Toc139278612)

[18. eQTpLot for Pan Tissue analysis for NKX2-2 in ASD. 21](#_Toc139278613)

[19. eQTpLot for Pan Tissue analysis for NTM in ASD. 22](#_Toc139278614)

[20. eQTpLot for Pan Tissue analysis for PINX1 in ASD. 22](#_Toc139278615)

[21. eQTpLot for Pan Tissue analysis for PTBP2 in ASD. 23](#_Toc139278616)

[22. eQTpLot for Pan Tissue analysis for PTPRE in ASD. 23](#_Toc139278617)

[23. eQTpLot for Pan Tissue analysis for SOX7 in ASD. 24](#_Toc139278618)

[24. eQTpLot for Pan Tissue analysis for SRPK2 in ASD. 24](#_Toc139278619)

[25. eQTpLot for Pan Tissue analysis for WNT3 in ASD. 25](#_Toc139278620)

[26. eQTpLot for Pan Tissue analysis for XKR6 in ASD. 25](#_Toc139278621)

[27. eQTpLot for Pan Tissue analysis for XRN2 in ASD. 26](#_Toc139278622)

[28. eQTpLot for Multi Tissue analysis for BLK in ASD. 27](#_Toc139278623)

[29. eQTpLot for Multi Tissue analysis for CIPC in ASD. 27](#_Toc139278624)

[30. eQTpLot for Multi Tissue analysis for CRHR1 in ASD. 28](#_Toc139278625)

[31. eQTpLot for Multi Tissue analysis for ERI1 in ASD. 28](#_Toc139278626)

[32. eQTpLot for Multi Tissue analysis for KANSL1 in ASD. 29](#_Toc139278627)

[33. eQTpLot for Multi Tissue analysis for KIZ in ASD. 29](#_Toc139278628)

[34. eQTpLot for Multi Tissue analysis for MANBA in ASD. 30](#_Toc139278629)

[35. eQTpLot for Multi Tissue analysis for MAPT in ASD. 30](#_Toc139278630)

[36. eQTpLot for Multi Tissue analysis for MSRA in ASD. 31](#_Toc139278631)

[37. eQTpLot for Multi Tissue analysis for NKX2-2 in ASD. 31](#_Toc139278632)

[38. eQTpLot for Multi Tissue analysis for NTM in ASD. 32](#_Toc139278633)

[39. eQTpLot for Multi Tissue analysis for PINX1 in ASD. 32](#_Toc139278634)

[40. eQTpLot for Multi Tissue analysis for PTBP2 in ASD. 33](#_Toc139278635)

[41. eQTpLot for Multi Tissue analysis for PTPRE in ASD. 33](#_Toc139278636)

[42. eQTpLot for Multi Tissue analysis for SOX7 in ASD. 34](#_Toc139278637)

[43. eQTpLot for Multi Tissue analysis for WNT3 in ASD. 34](#_Toc139278638)

[44. eQTpLot for Multi Tissue analysis for XRN2 in ASD. 35](#_Toc139278639)

# Regional Plots

Regional Plots LocusZoom tool (http://locuszoom.org/) was employed to construct regional plots for the regions containing ASD associated genes with colocalizing eQTL signals. To this aim, meta-analysis data including marker name, p-values, odds ratio (OR), chromosome position (start-end), and index SNP/gene name were specified for the analysis. The source of LD information used to construct the r^2^ correlation matrix between SNPs in these regional plots was retrieved from hg19/1000 Genomes Nov 2014 EUR (European). The rest of the optional controls were used as default^5^.


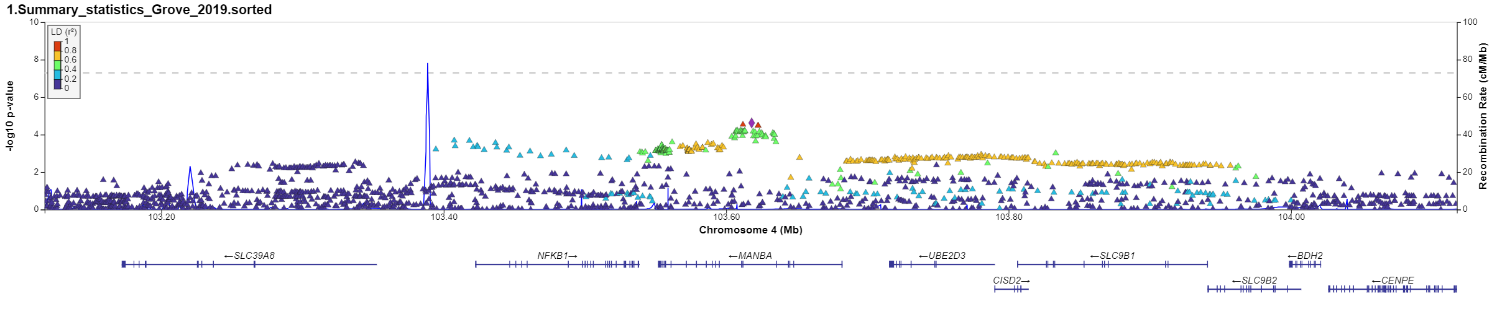

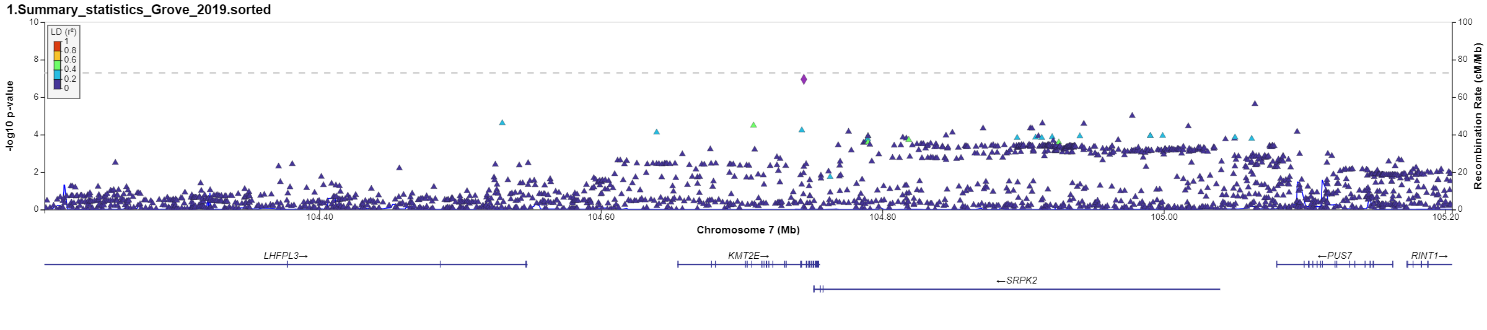


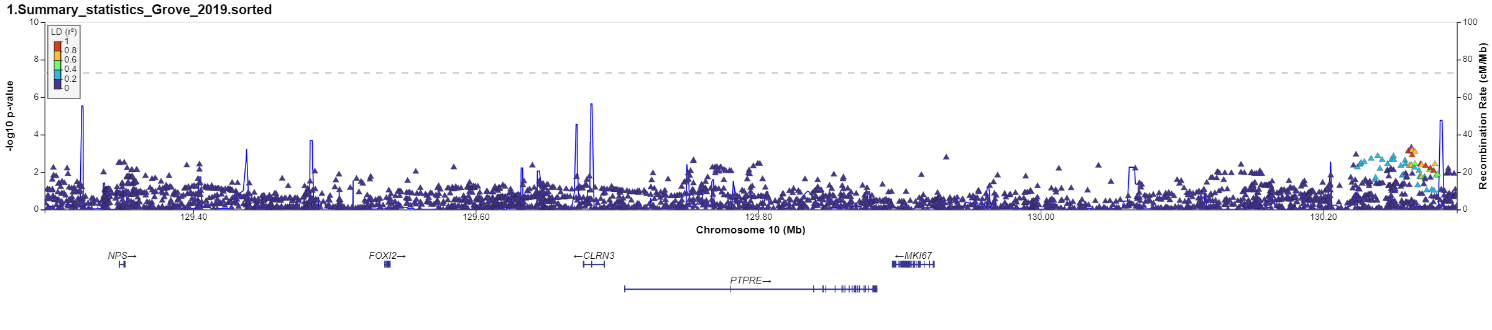


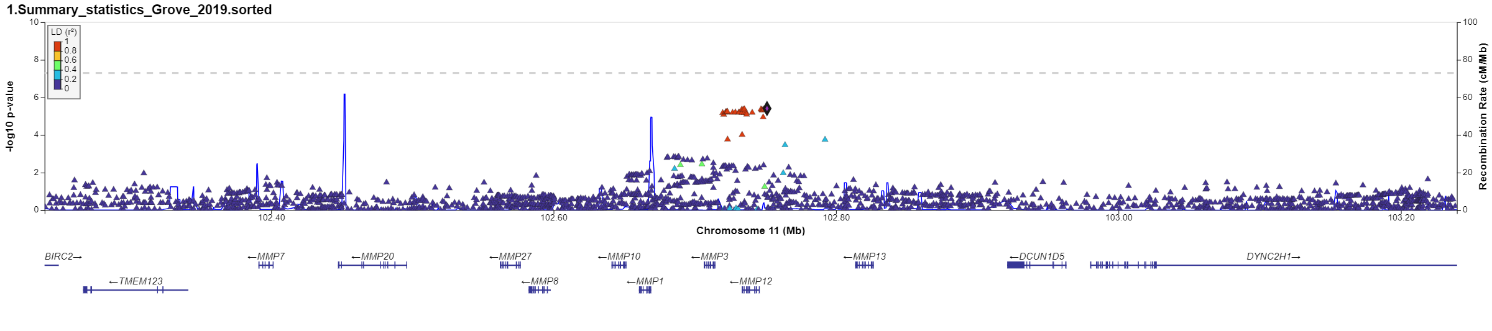


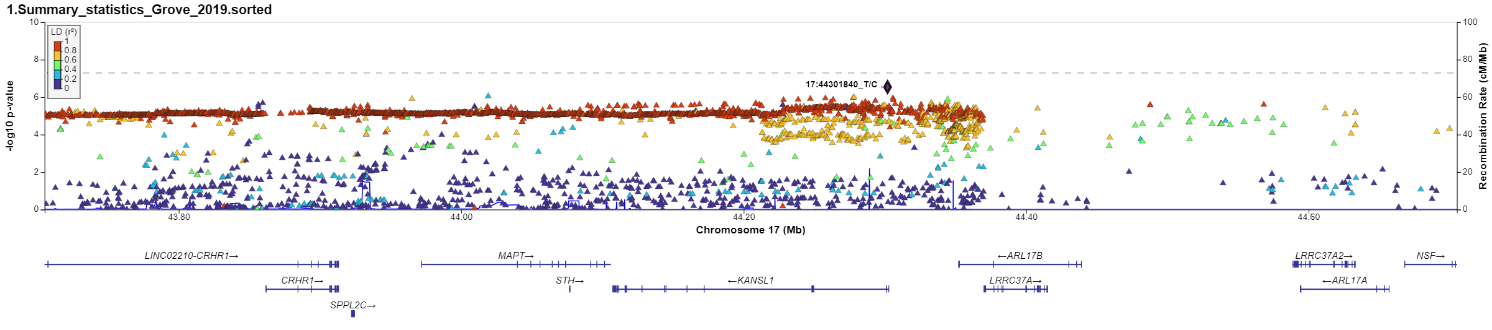


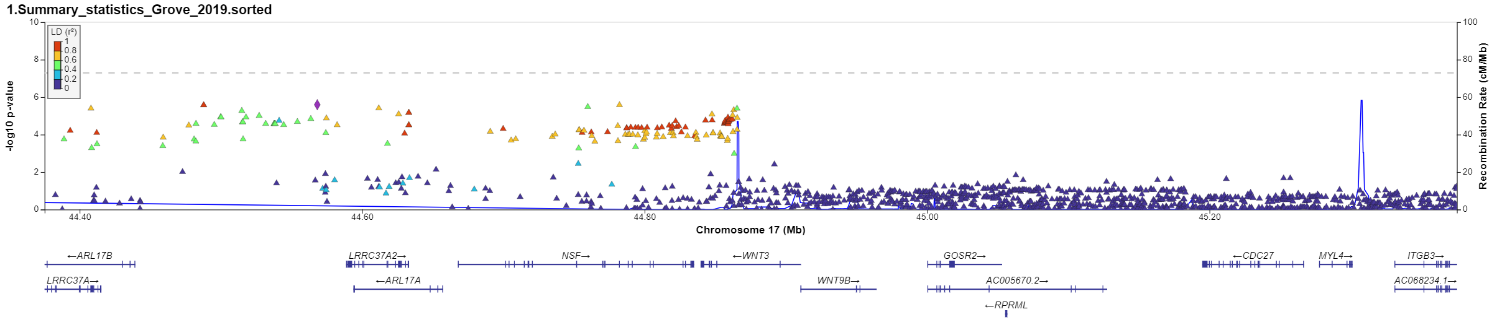


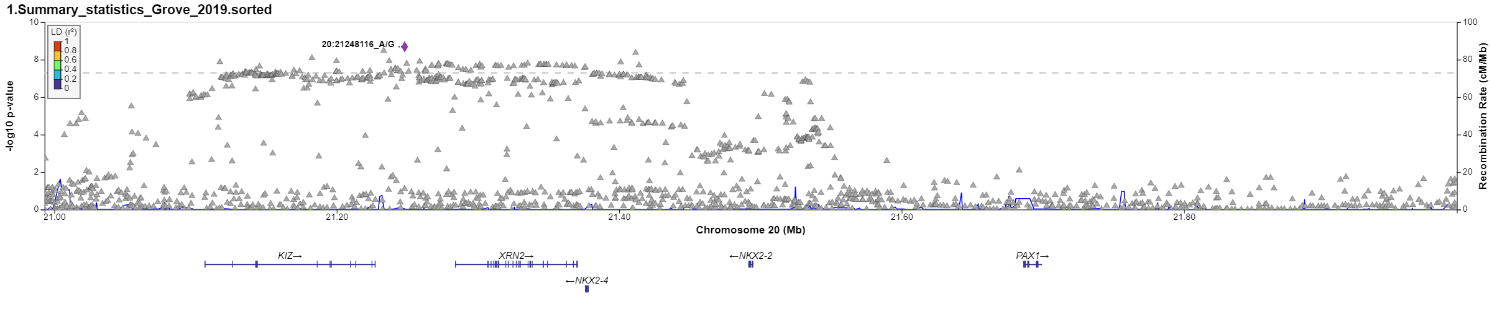


Supplementary Figure 1**:** Regional association plots for ASD genes selected by eQTL colocalization analysis. eQTpLot has highlighted *MANBA*, *SRPK2*, *PTPRE, MMP12, CRHR1*, *KANSL1*, *MAPT*, *WNT3* and *NKX2-2* (chromosomes 4, 7, 10, 11, 17, 20) as potentially causal genes. The regional association plot for chromosome 20 has no usable LD information for index SNP.

# eQTL plots

In figures 2–44 we provide the eQTL-GWAS colocalization plots for 27 ASD associated genes collected from bibliography and selected for eQTL colocalization study (see Supplementary Table 1). Supplementary figures 2-27 show the results for the Pan Tissue Analysis (varian’ts effect on candidate gene expression across all tissues). Supplementary figures 28-44 show the results for the Multi Tissue Analysis (varian’ts effect on candidate gene expression across brain tissue). The plots' interpretation is included in Supplementary Figure 2 as a reference (for further explanation see the Methods' section).

After filtering by the R^2^ and LD tresholds, fewer than two SNPs were present in the GWAS' summary statistics for *PINX1* and *SOX7,*  hence LD data was removed when plotting these genes.

No plot was generated for the genes raising errors (Supplementary Table 2).

##
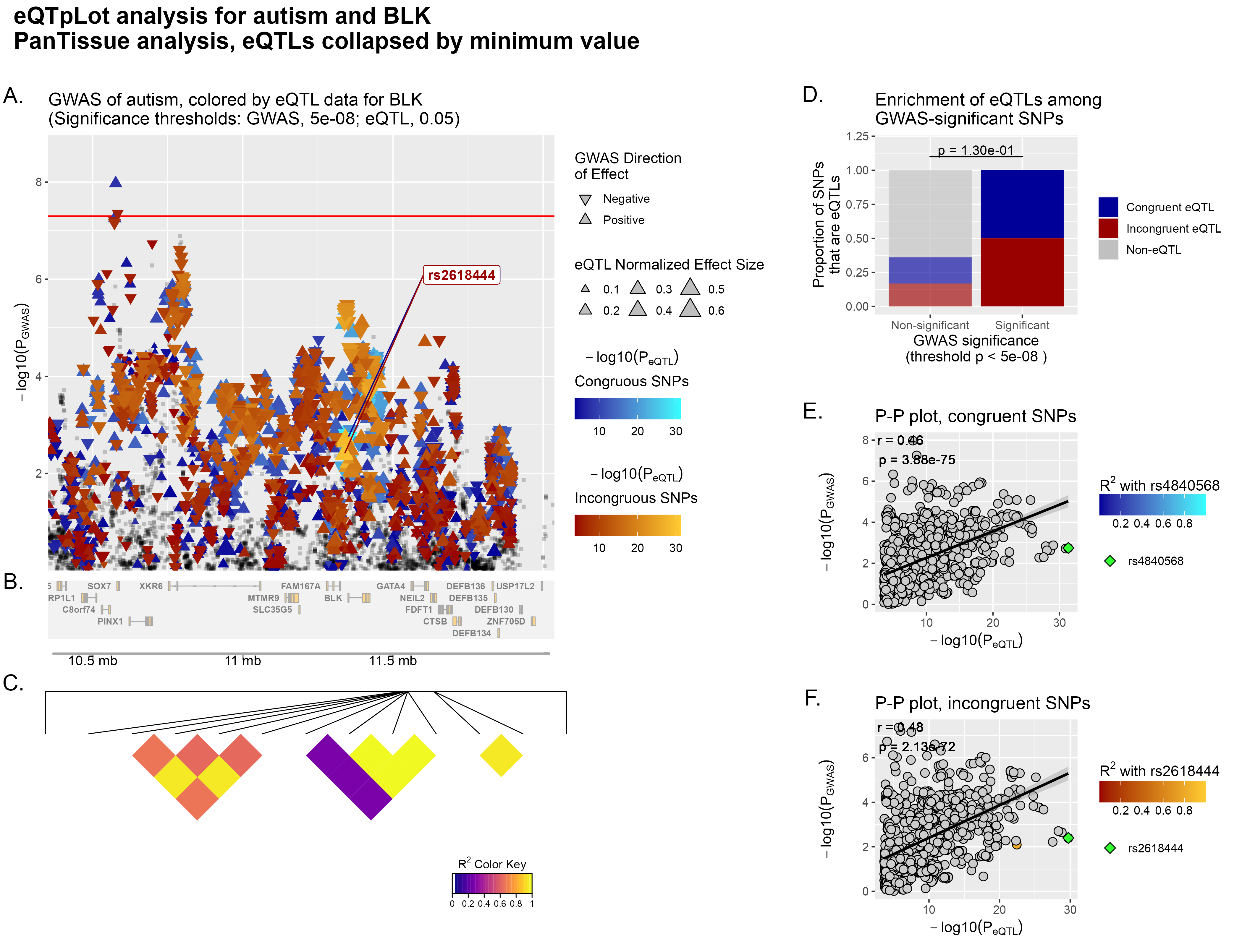
Pan Tissue Analysis

Supplementary Figure 2**:** eQTpLot for Pan Tissue analysis for *BLK* in ASD. Panel A shows the locus of interest, containing *BLK*, with chromosomal space indicated along the horizontal axis. Within this plot, variants that lack eQTL data for the specified gene or do not meet the significance threshold are plotted as grey squares. On the opposite, variants that are determined to act as eQTLs for MAPT are plotted as colored triangles, with a color gradient corresponding to the inverse magnitude of its effect on gene expression or p-value. Congruous effects are plotted using a blue color scale, in contrast to variants having an incongruous effect, in red. The size of each triangle is proportional to the eQTL normalized effect size (NES), while the directionality of each triangle corresponds to the direction of effect of the variant on the GWAS trait. Horizontal red line marks the significance threshold in GWAS (p-value=5x10^-08^). Finally, we show at the bottom of the plot a depiction of all genes’ genomic positions that fall inside the LOI (Panel B) and a heatmap of LD information of all *BLK* eQTL variants (Panel C). Panel D depicts the enrichment of *BLK* eQTLs among GWAS-significant/non-significant variants, while panel E-F depict the correlation between P_GWAS_ and P_eQTL_ for *BLK* and ASD, with the computed Pearson correlation coefficient (r) and p-value (p) displayed on the plot. The lead variants, rs4840568 and rs2618444, are identified in each graph (by default the upper-right-most variant on the P-P plot), with all other variants plotted using a color scale corresponding to their squared coefficient of linkage correlation with this lead variant (if available). For reference, the same lead variants are also labelled in panel A.

**
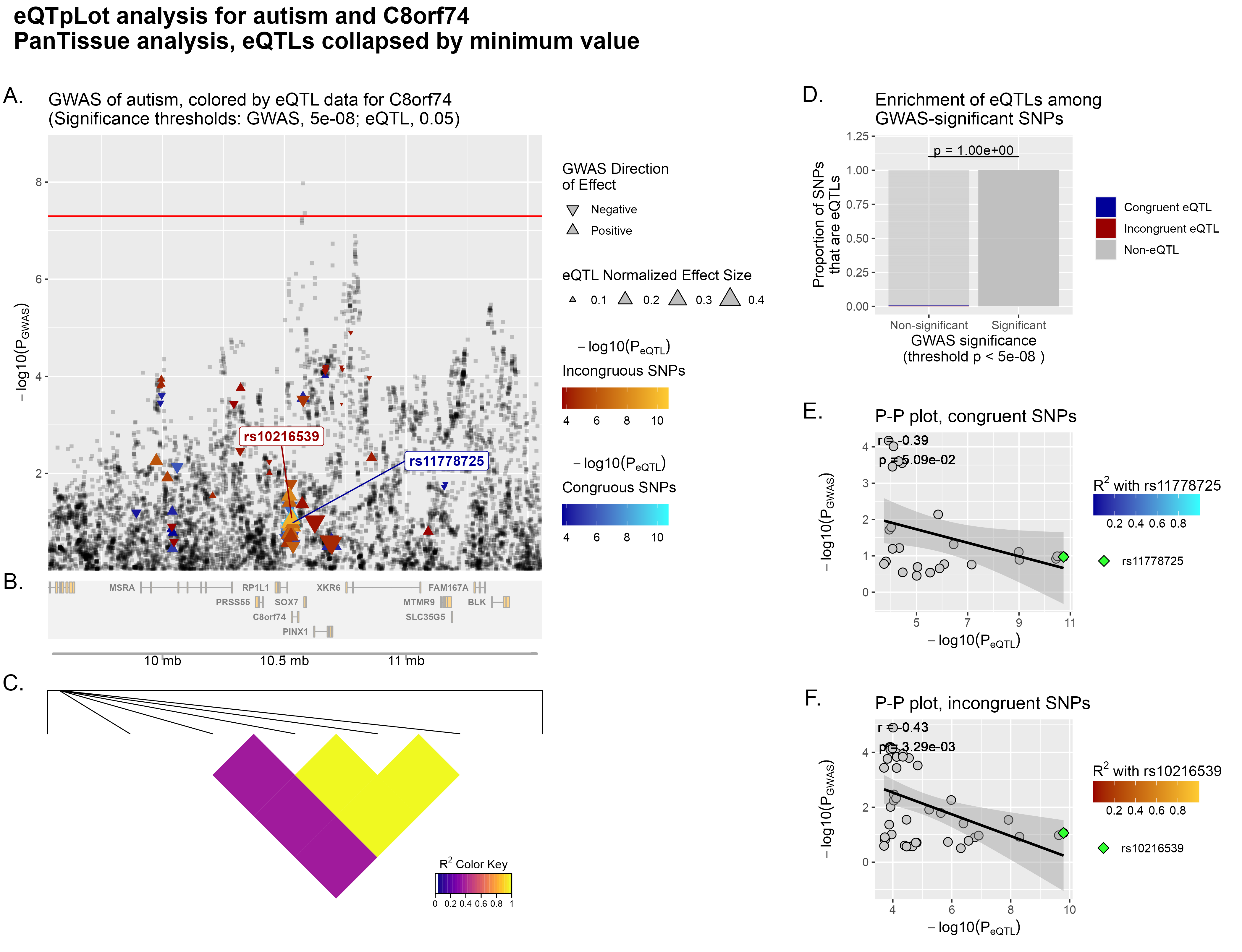
**

Supplementary Figure 3**:** eQTpLot for Pan Tissue analysis for *C8orf74* in ASD.


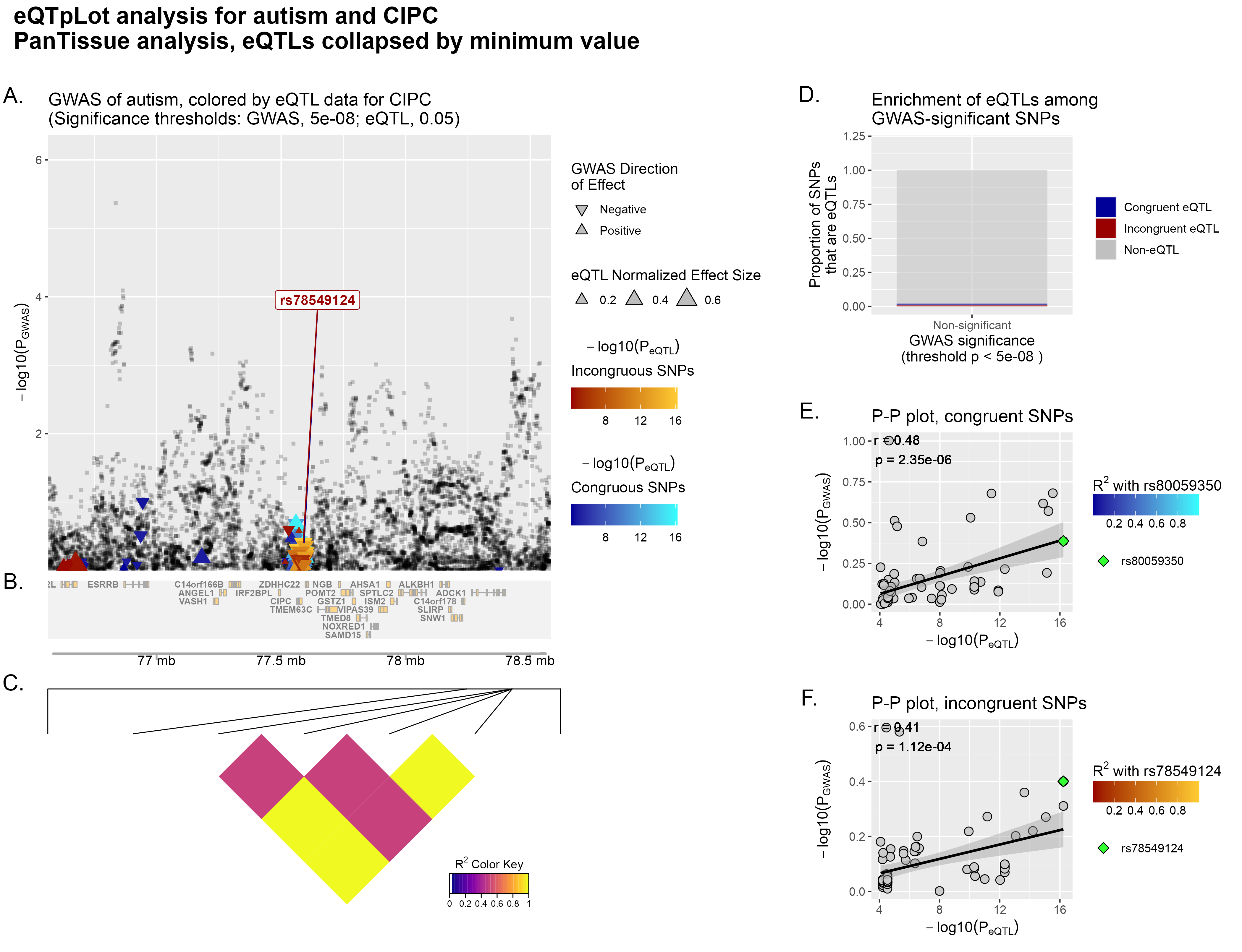


Supplementary Figure 4**:** eQTpLot for Pan Tissue analysis for *CIPC* in ASD.


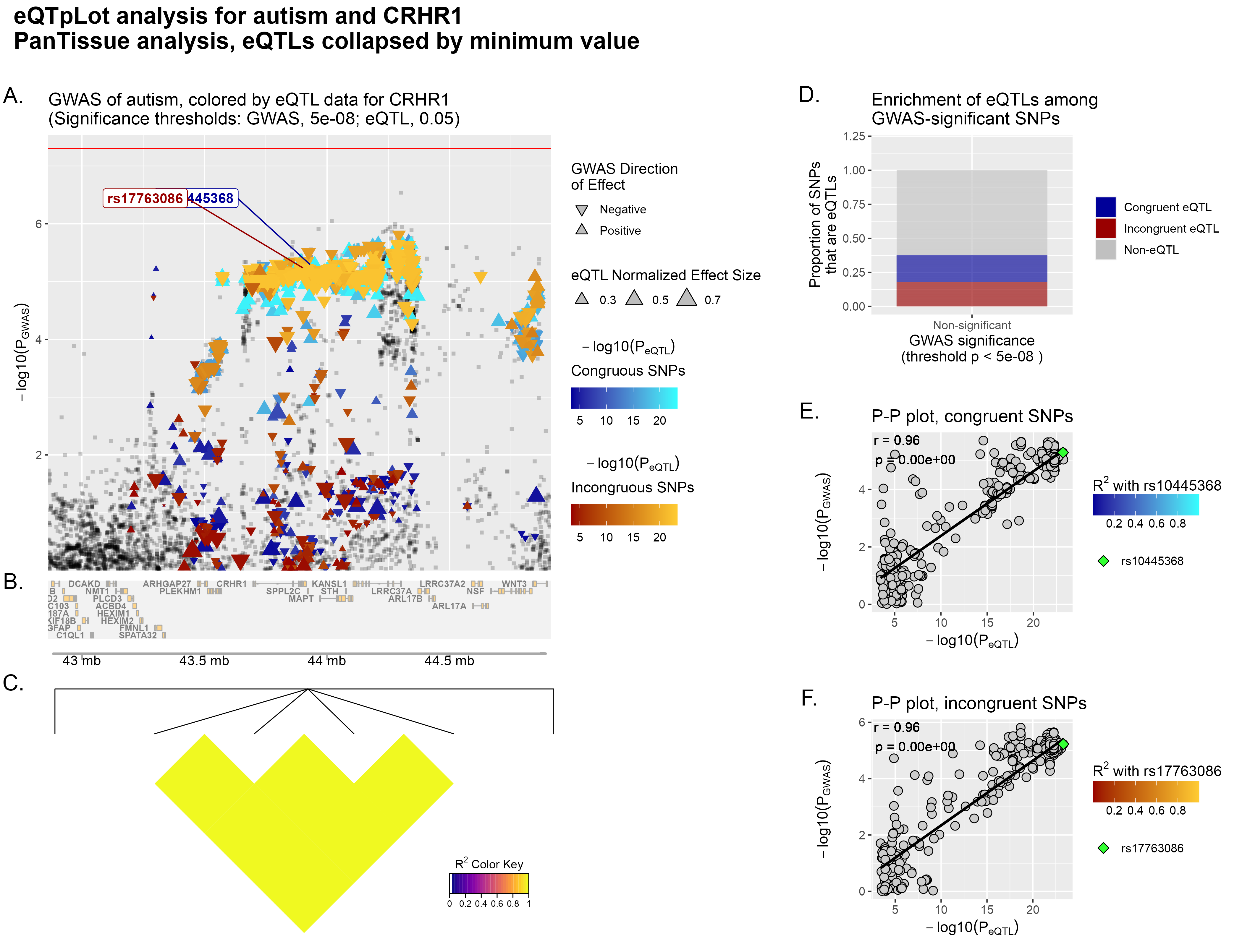


Supplementary Figure 5**:** eQTpLot for Pan Tissue analysis for *CRHR1* in ASD.


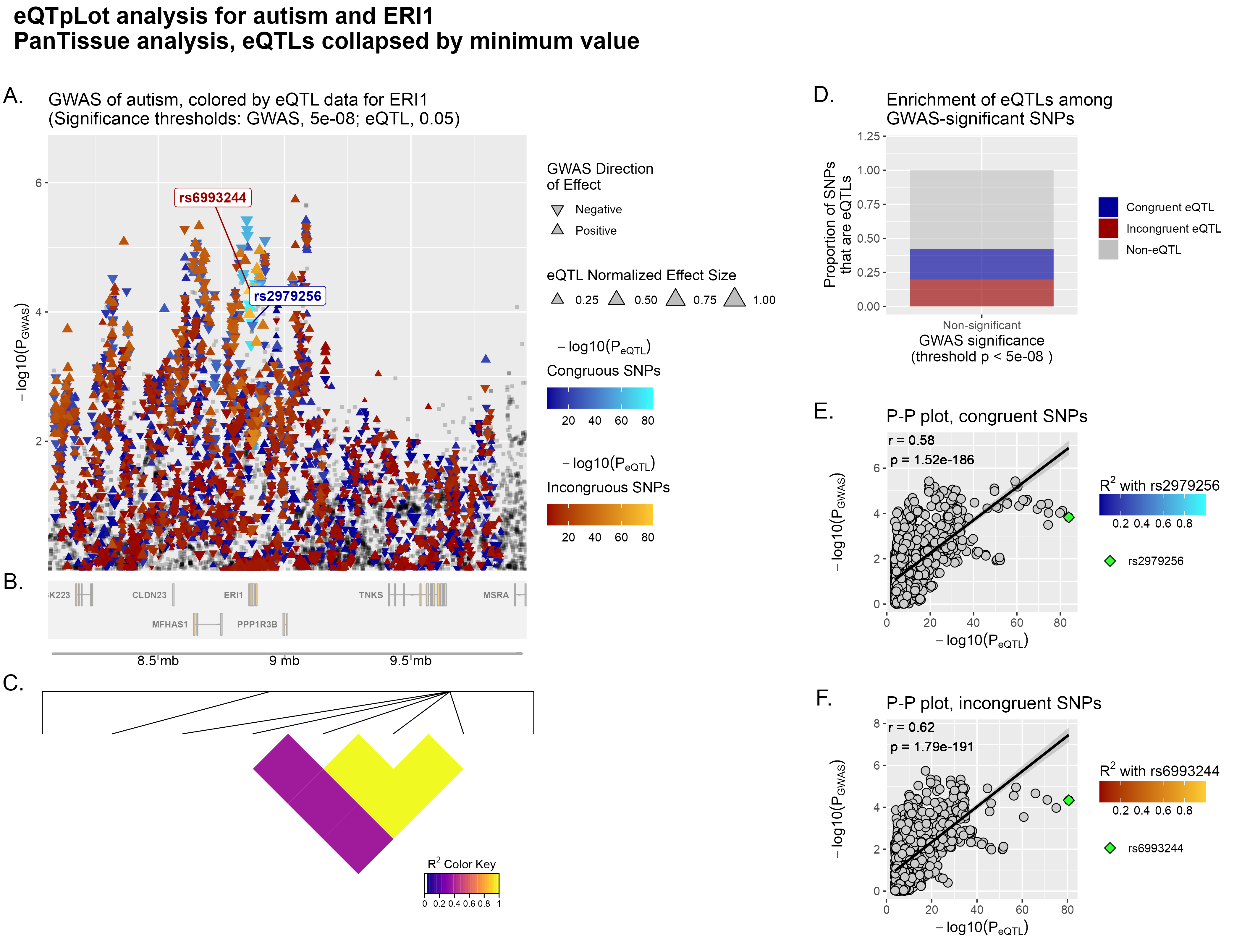


Supplementary Figure 6**:** eQTpLot for Pan Tissue analysis for *ERI1* in ASD.


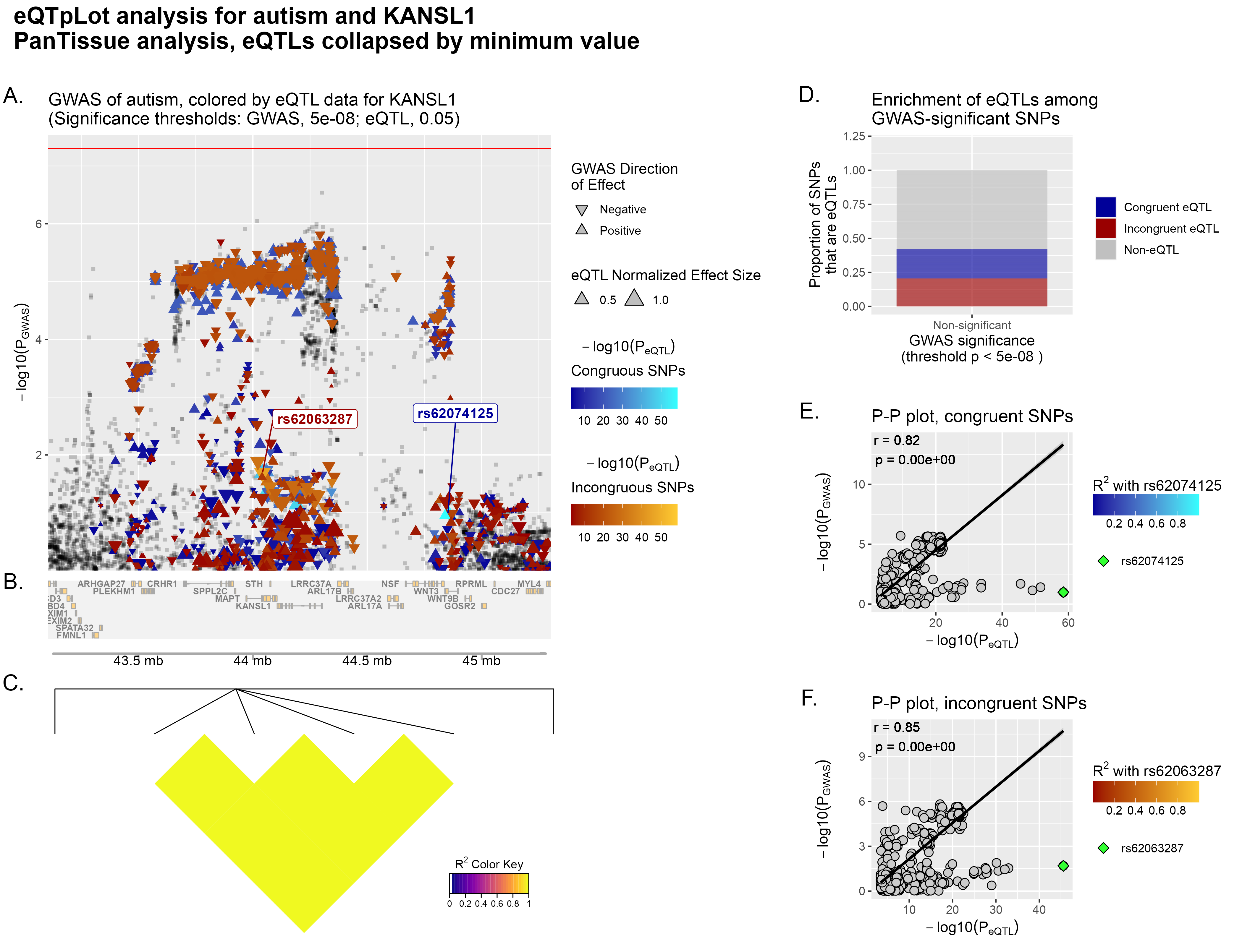


Supplementary Figure 7**:** eQTpLot for Pan Tissue analysis for *KANSL1* in ASD.

**
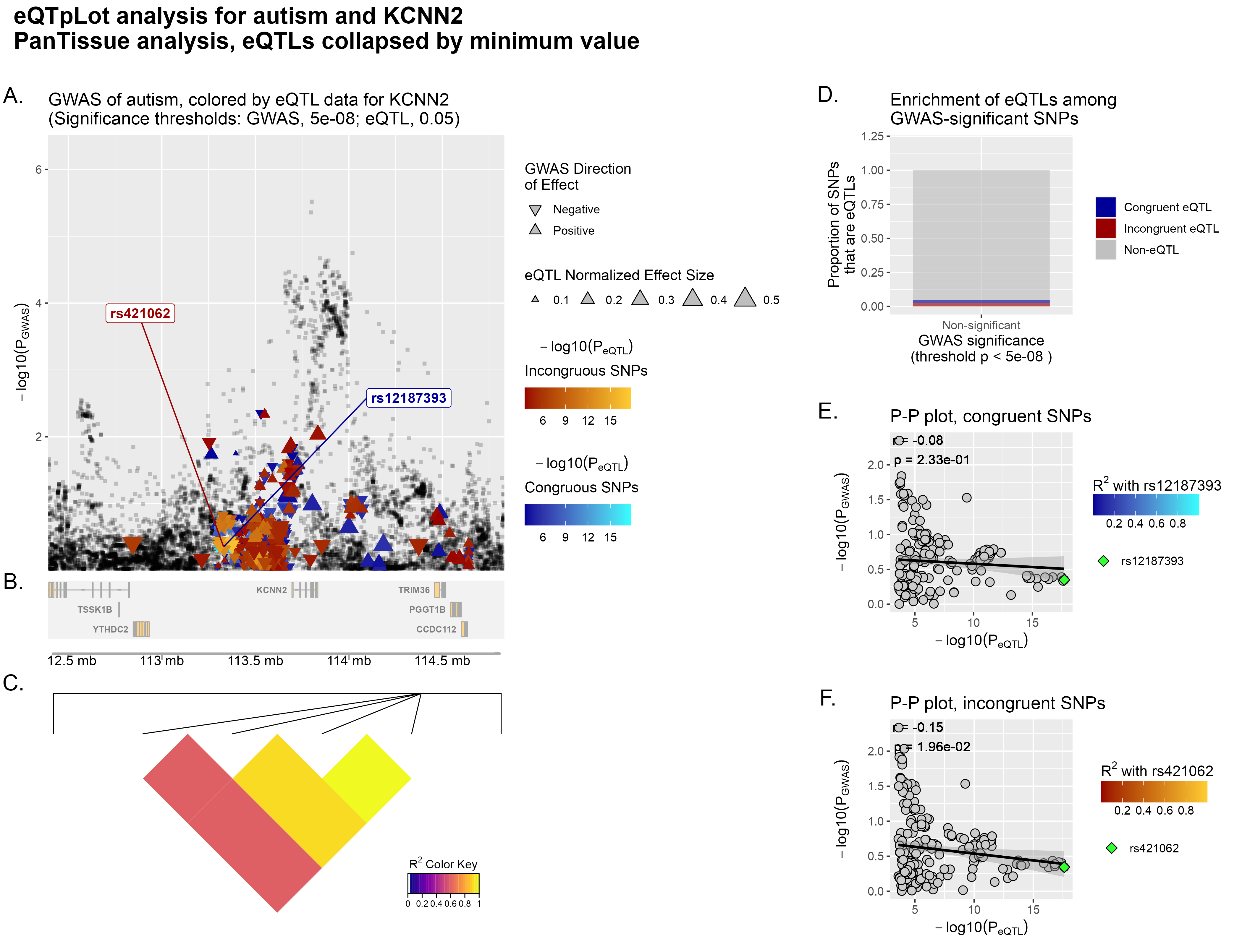
**

Supplementary Figure 8**:** eQTpLot for Pan Tissue analysis for *KCNN2* in ASD.


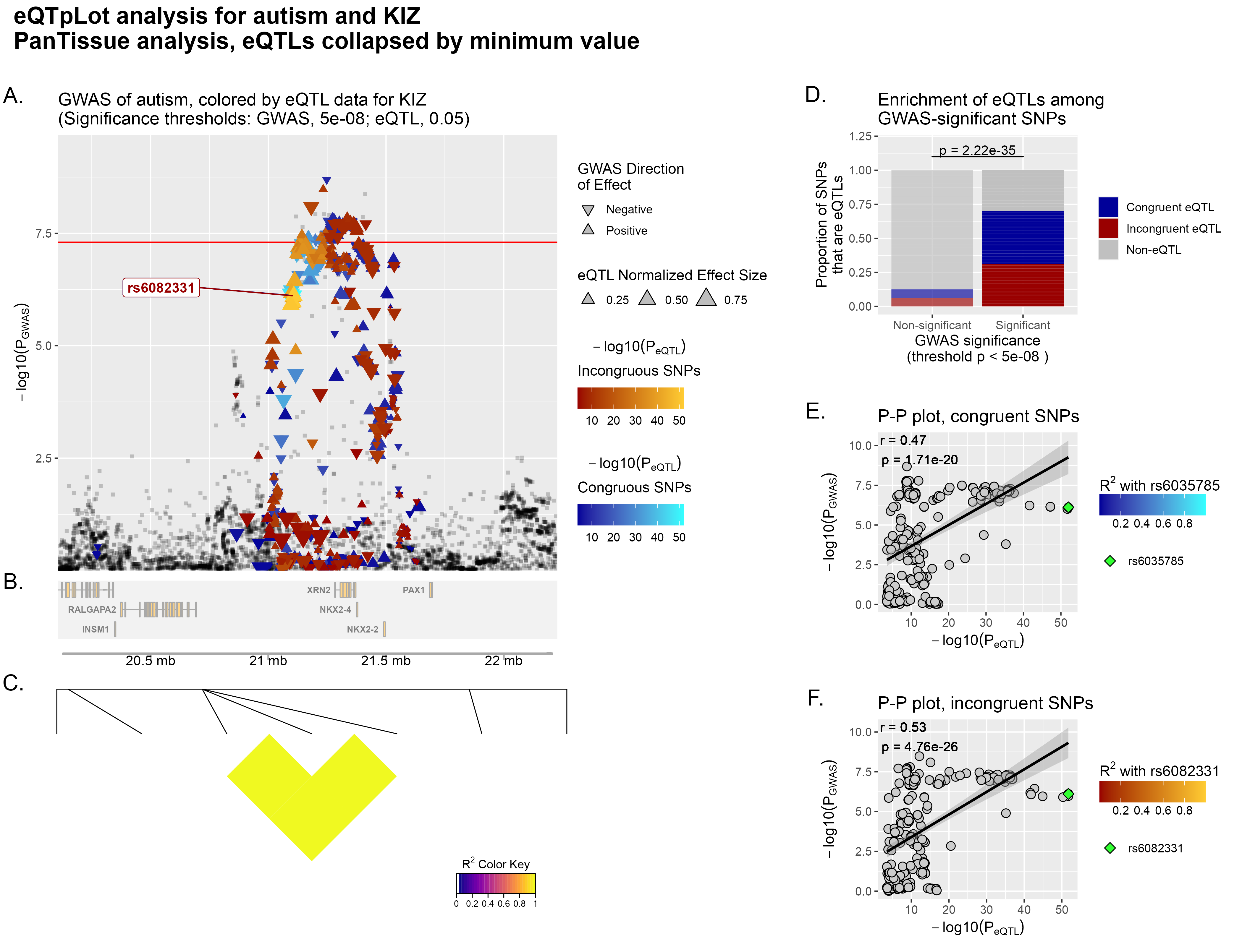


Supplementary Figure 9**:** eQTpLot for Pan Tissue analysis for *KIZ* in ASD.


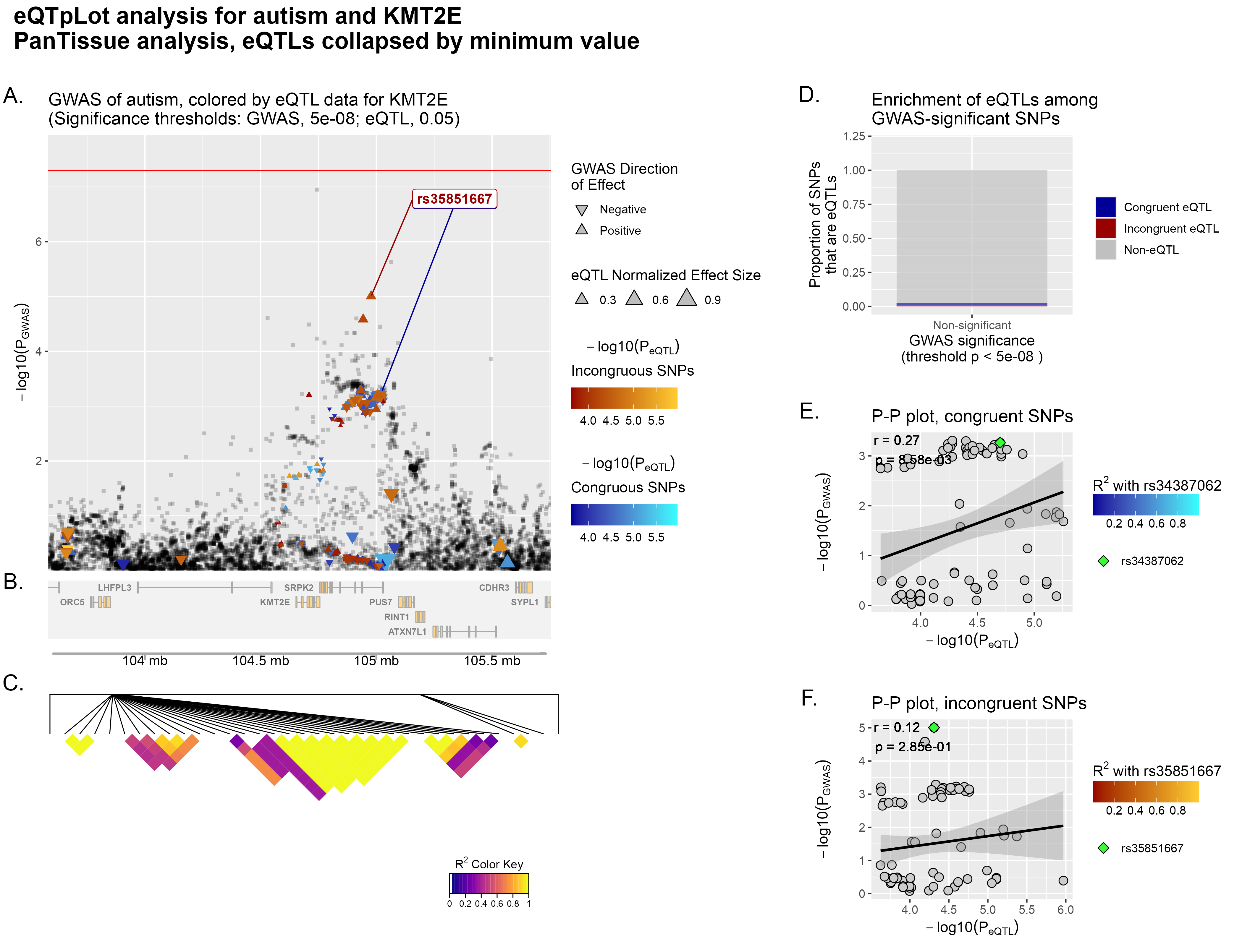
Supplementary Figure 10**:** eQTpLot for Pan Tissue analysis for *KMT2E* in ASD.


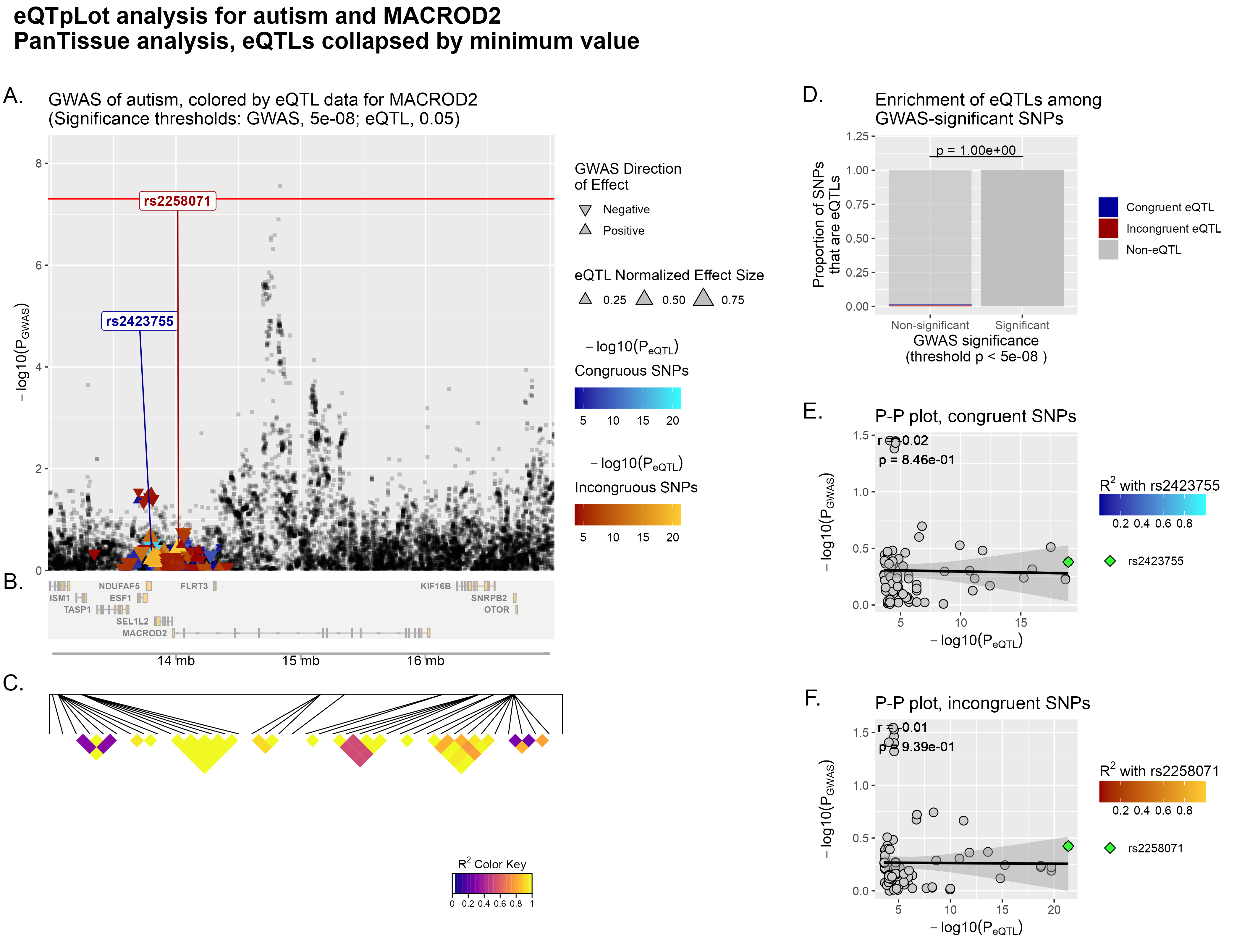
Supplementary Figure 11**:** eQTpLot for Pan Tissue analysis for *MACROD2* in ASD.


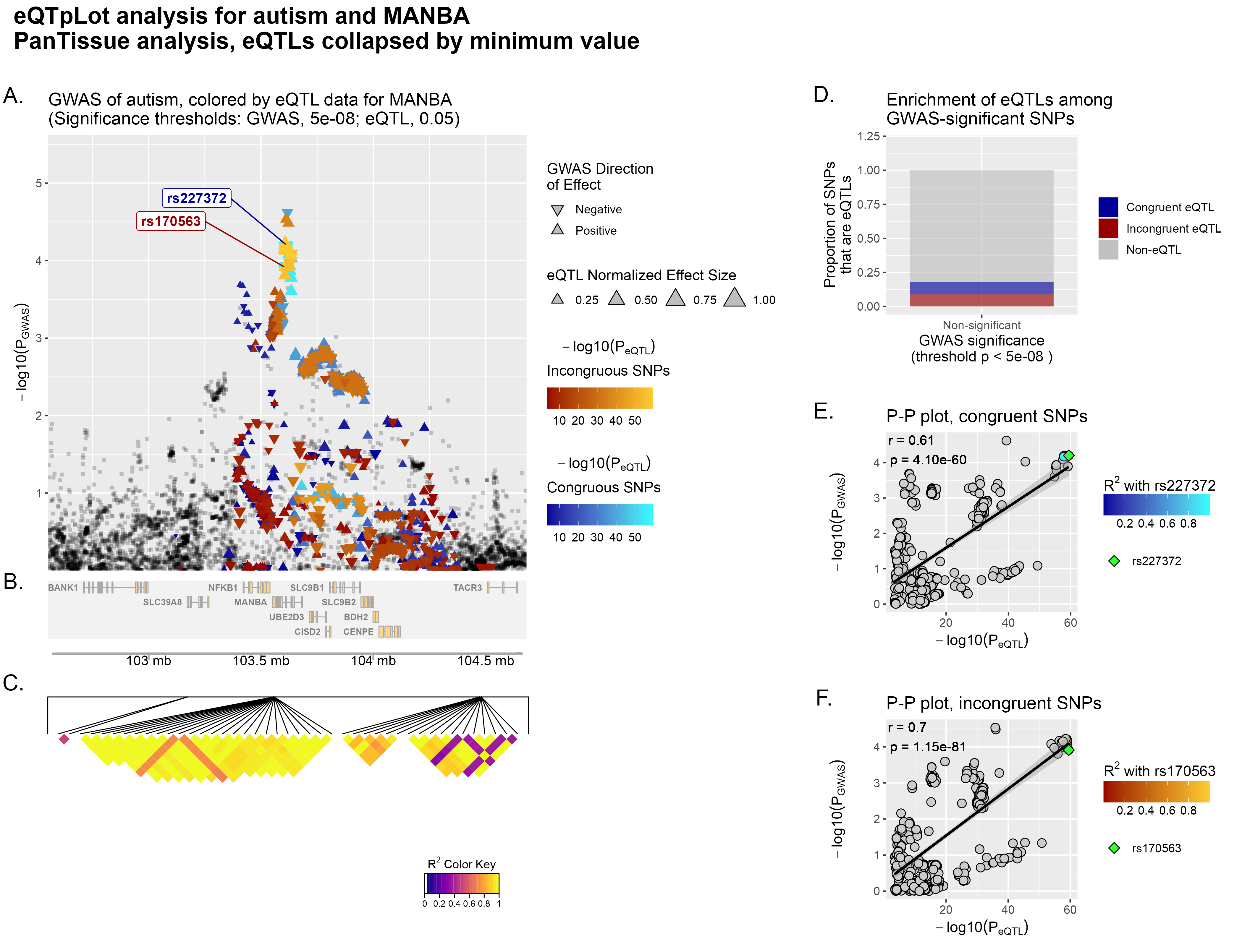


Supplementary Figure 12**:** eQTpLot for Pan Tissue analysis for *MANBA* in ASD.


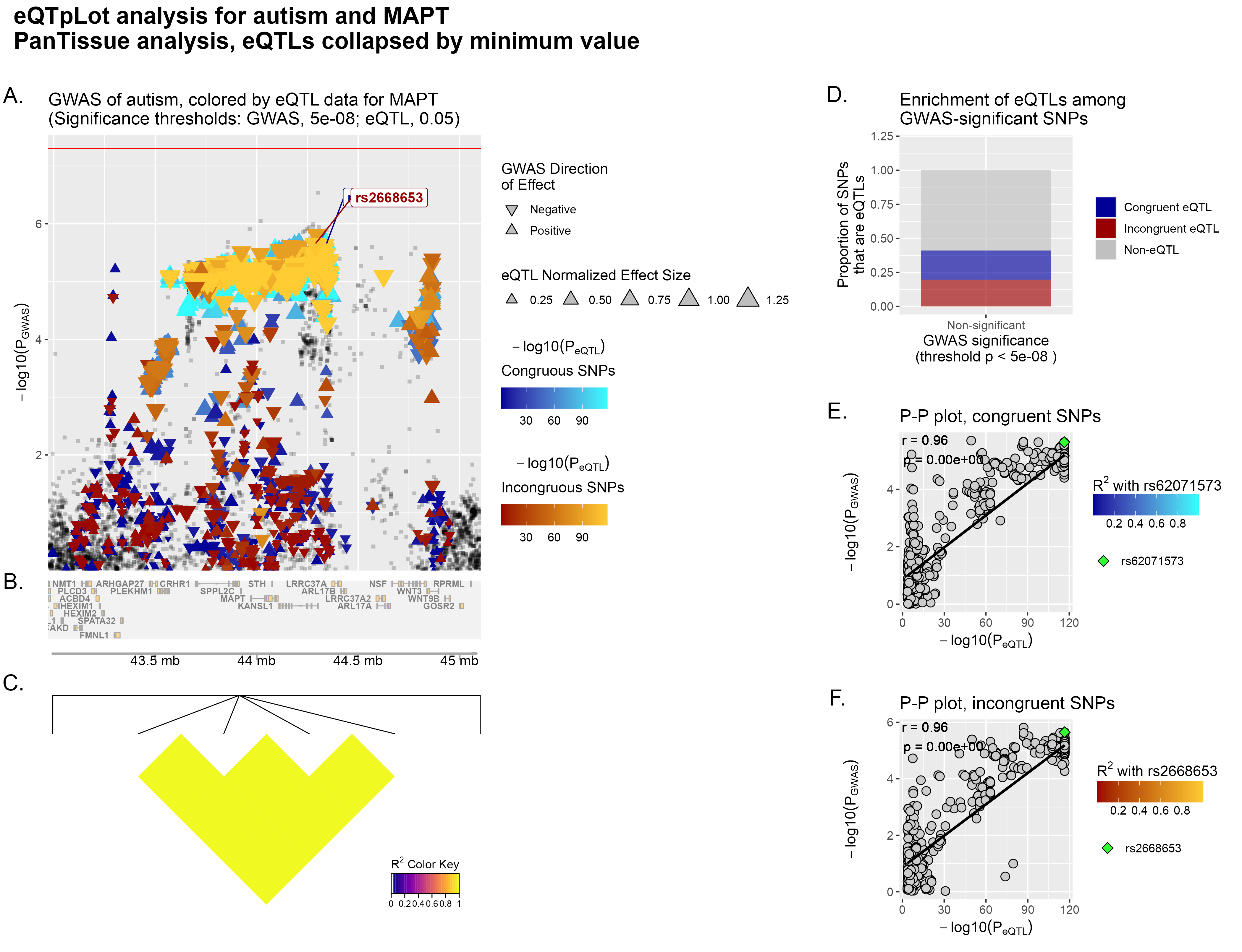


Supplementary Figure 13**:** eQTpLot for Pan Tissue analysis for *MAPT* in ASD.


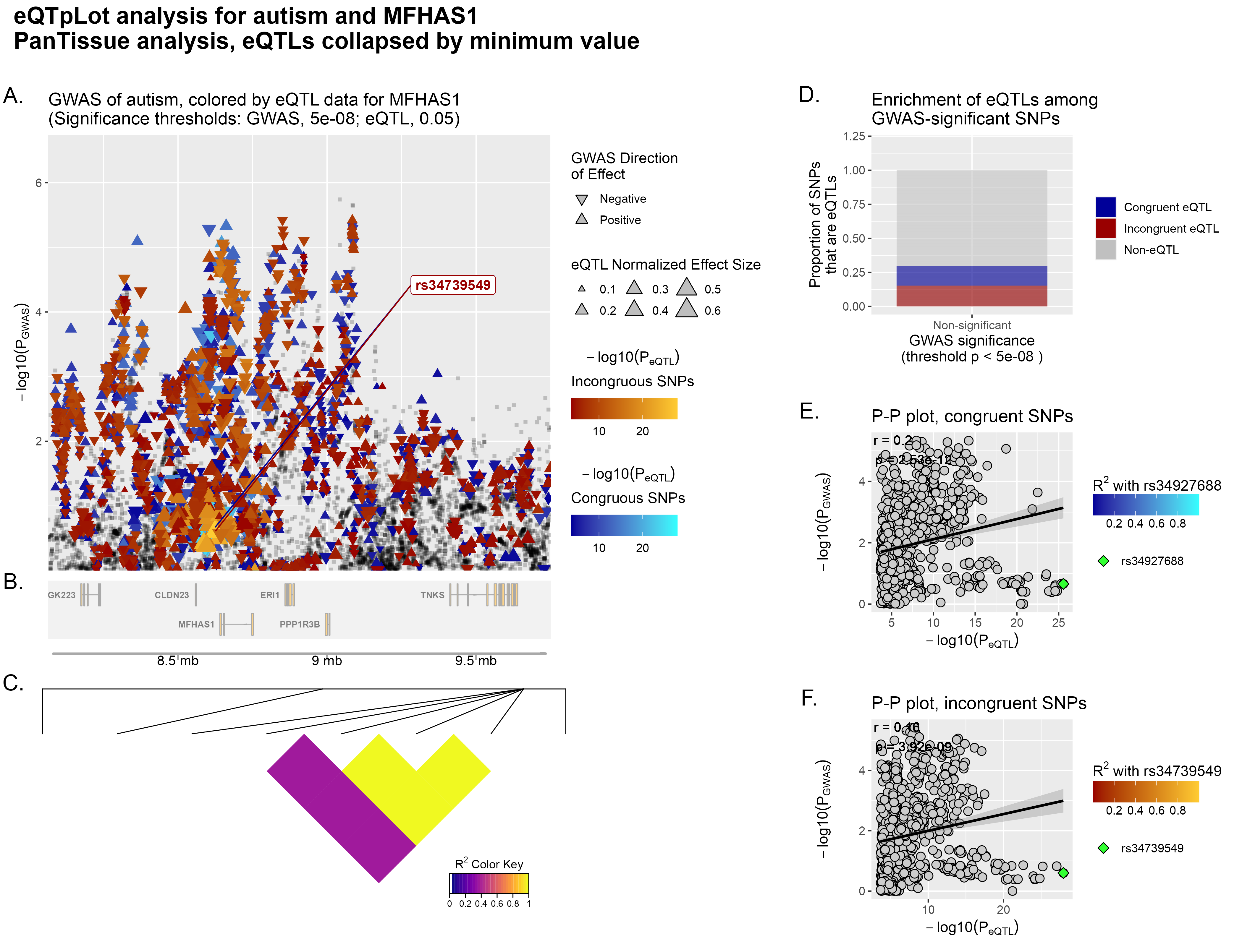


Supplementary Figure 14**:** eQTpLot for Pan Tissue analysis for *MFHAS1* in ASD.

**
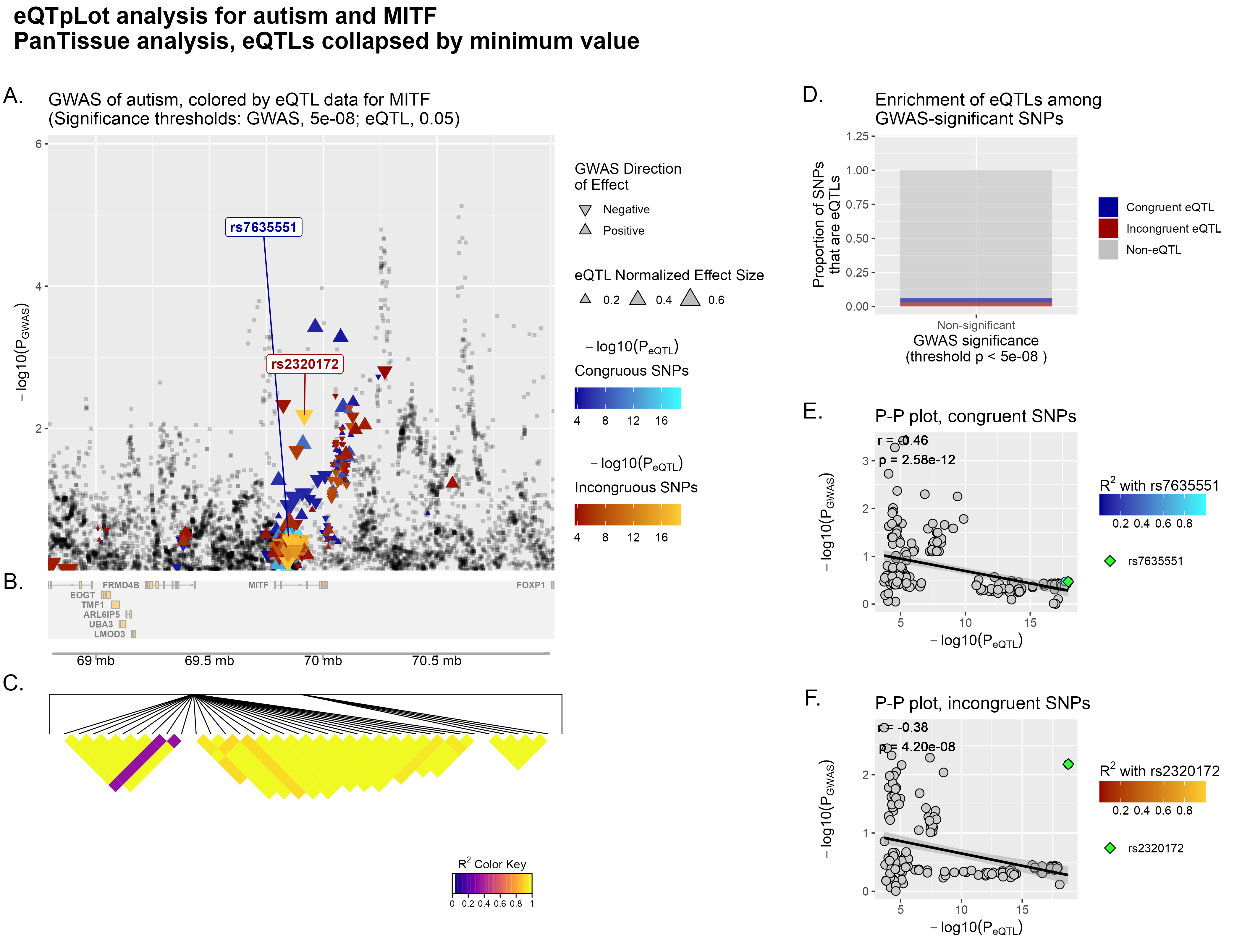
**

Supplementary Figure 15**:** eQTpLot for Pan Tissue analysis for *MITF* in ASD.


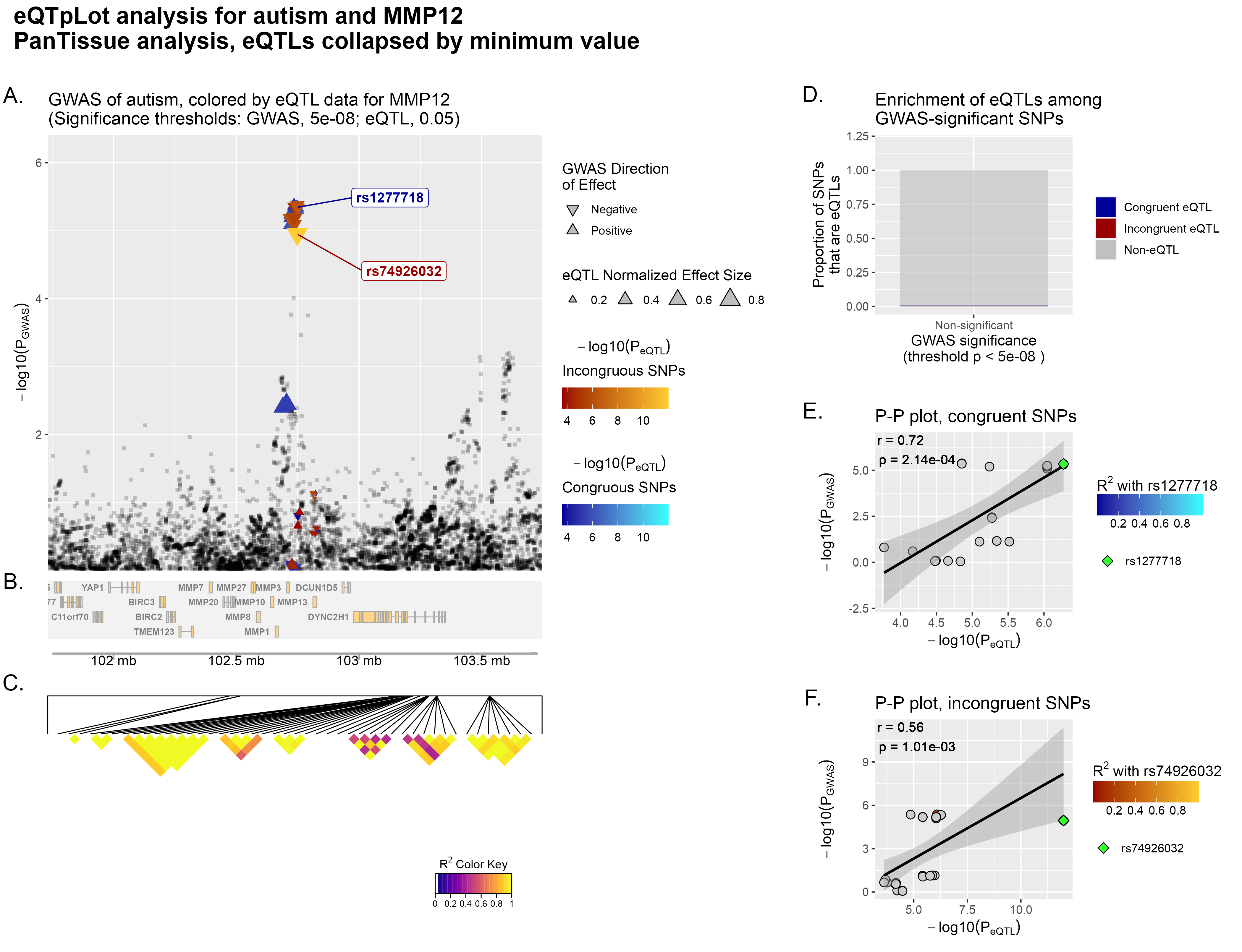


Supplementary Figure 16**:** eQTpLot for Pan Tissue analysis for *MMP12* in ASD.

**
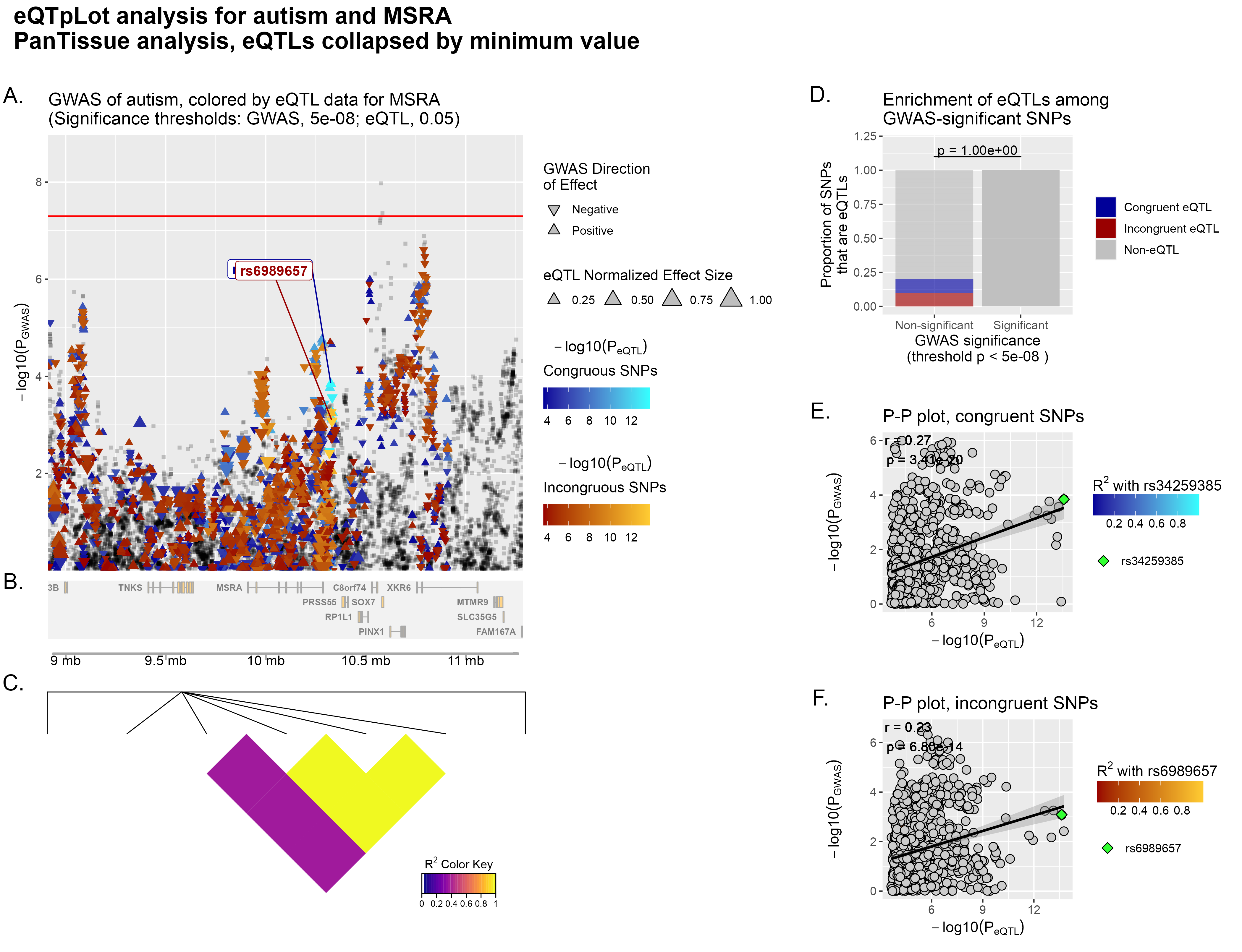
**

Supplementary Figure 17**:** eQTpLot for Pan Tissue analysis for *MSRA* in ASD.

**
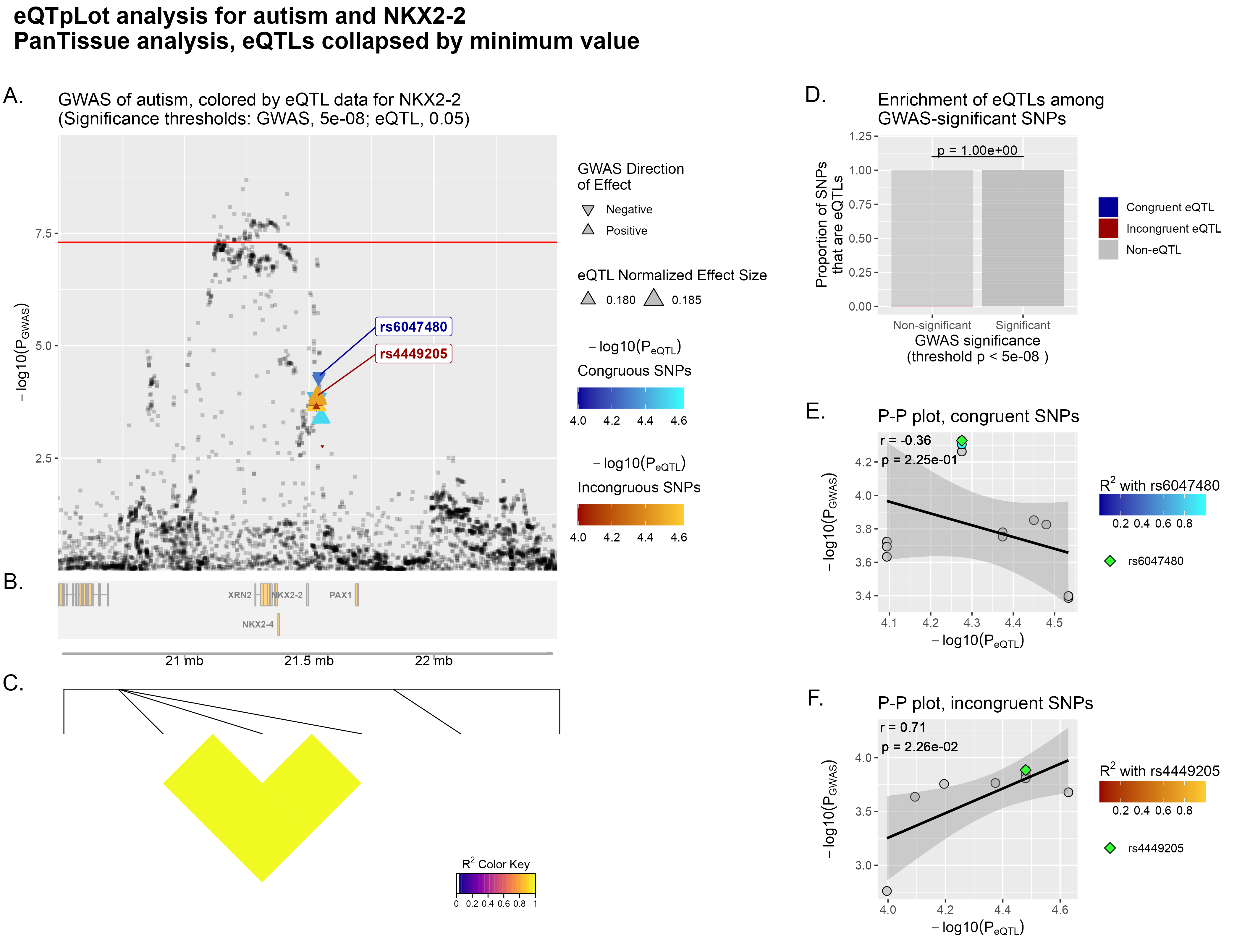
**

Supplementary Figure 18**:** eQTpLot for Pan Tissue analysis for *NKX2-2* in ASD.


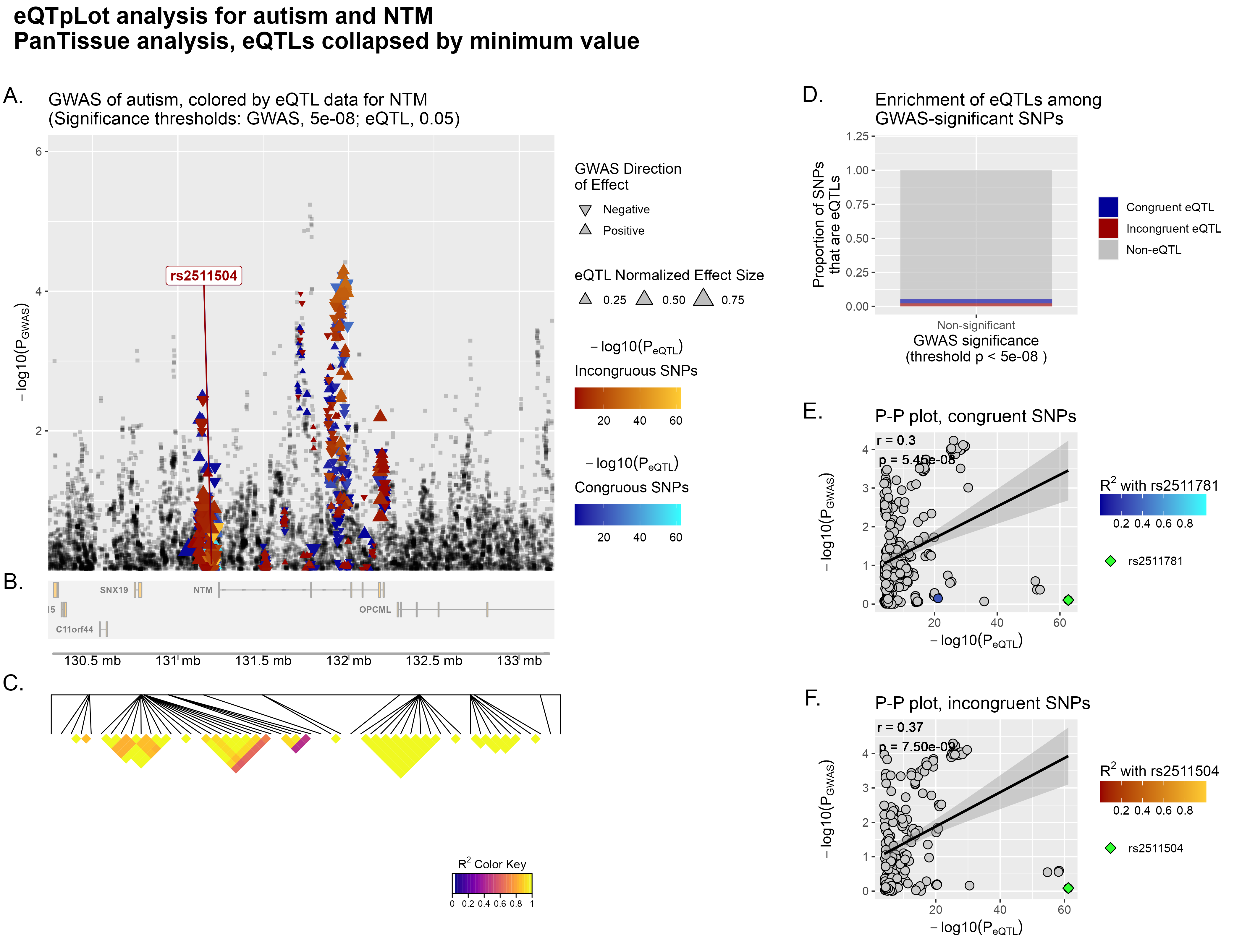


Supplementary Figure 19**:** eQTpLot for Pan Tissue analysis for *NTM* in ASD.


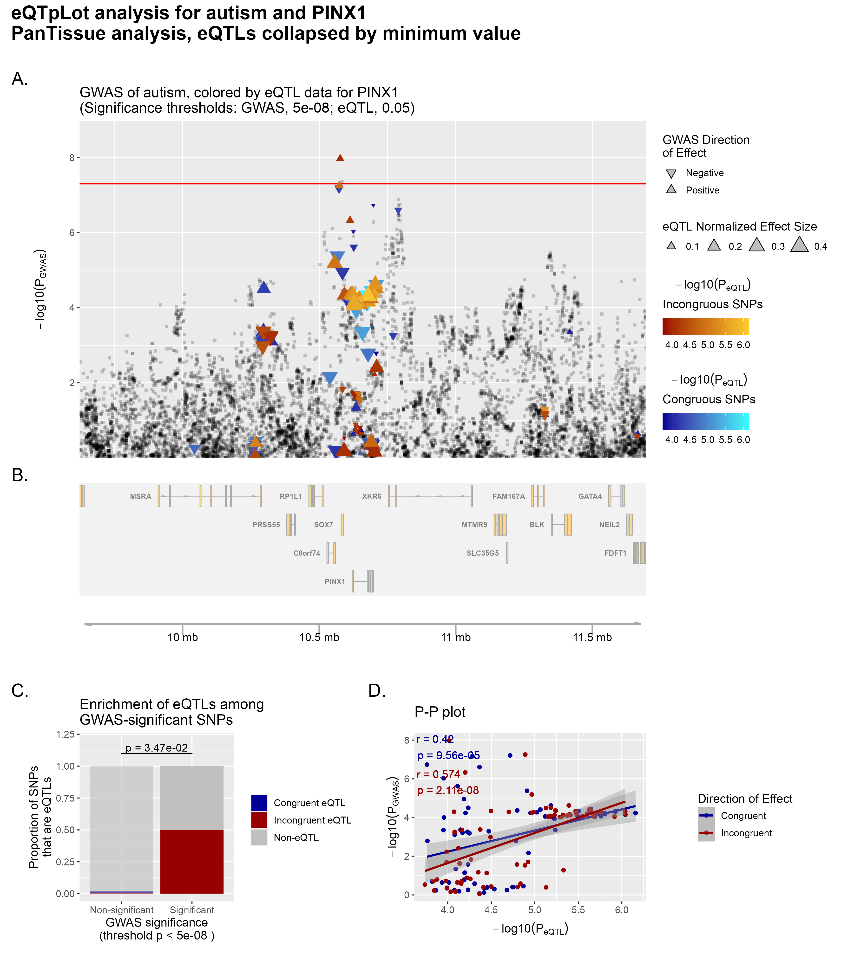


Supplementary Figure 20**:** eQTpLot for Pan Tissue analysis for *PINX1* in ASD. Linkage disequilibrium (LD) data is not included due to the fact that, after applying the supplied R2 and LD thresholds to filter the LD data, fewer than 2 SNPs that are also present in the GWAS summary statistics remain.


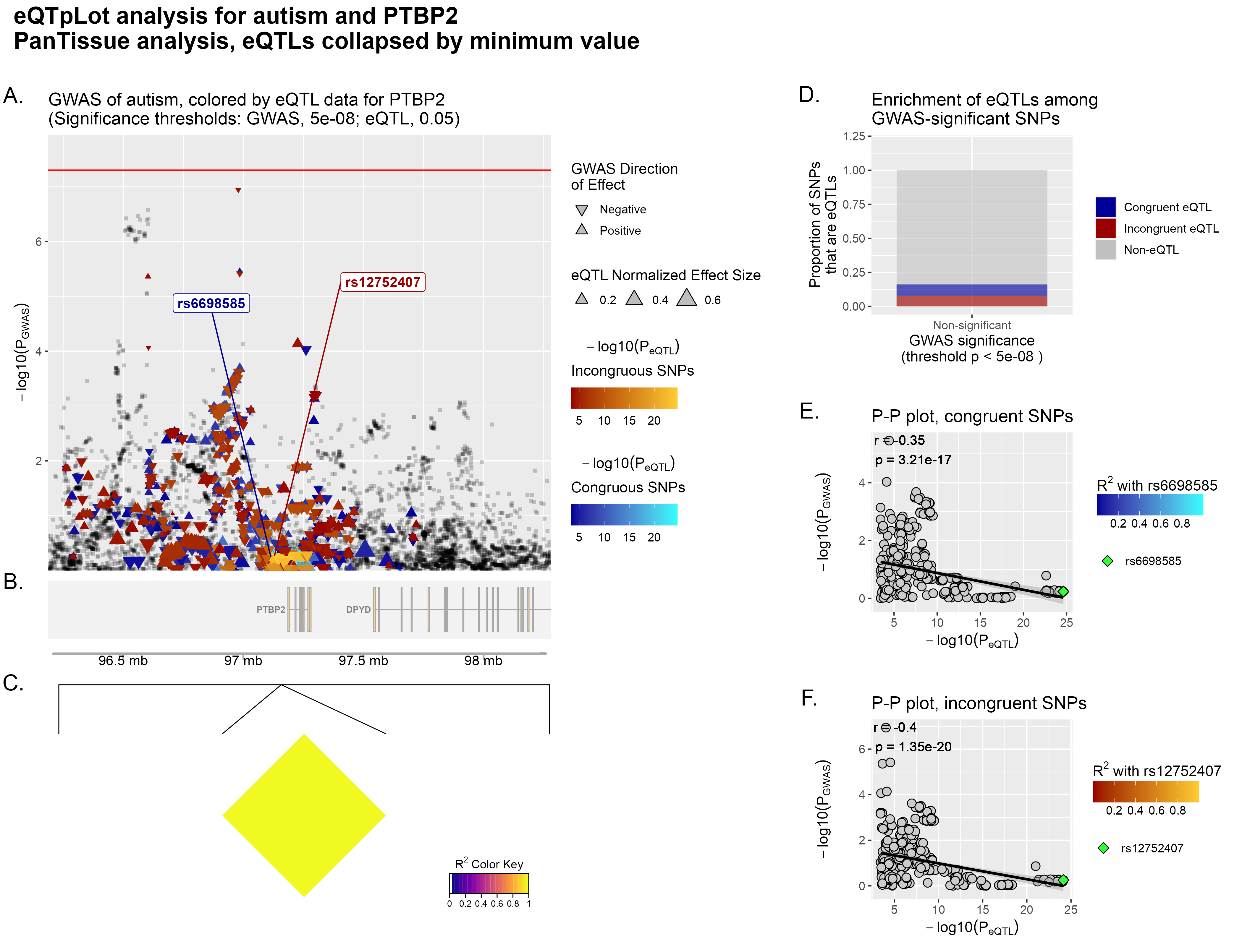
Supplementary Figure 21**:** eQTpLot for Pan Tissue analysis for *PTBP2* in ASD.


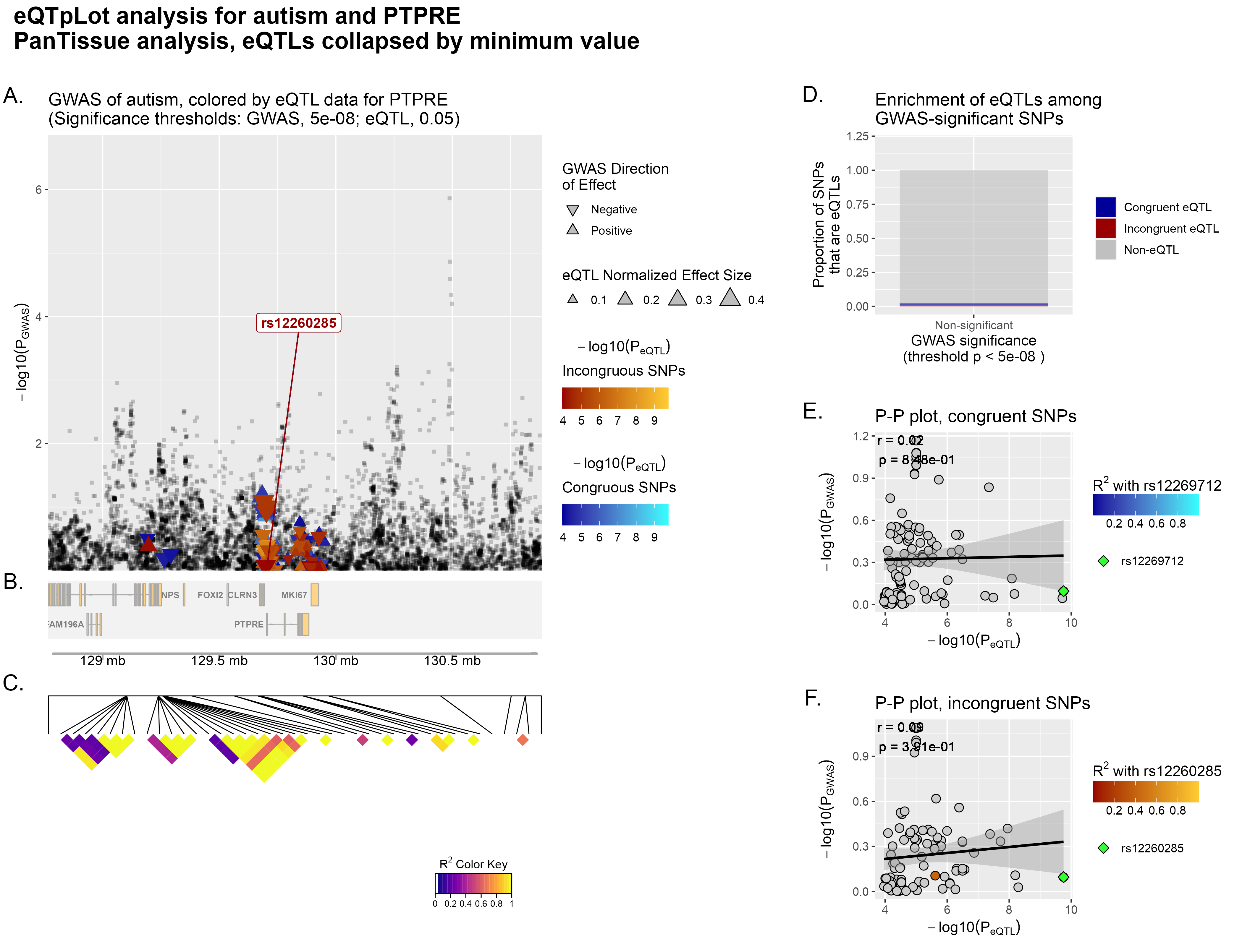


Supplementary Figure 22**:** eQTpLot for Pan Tissue analysis for *PTPRE* in ASD.


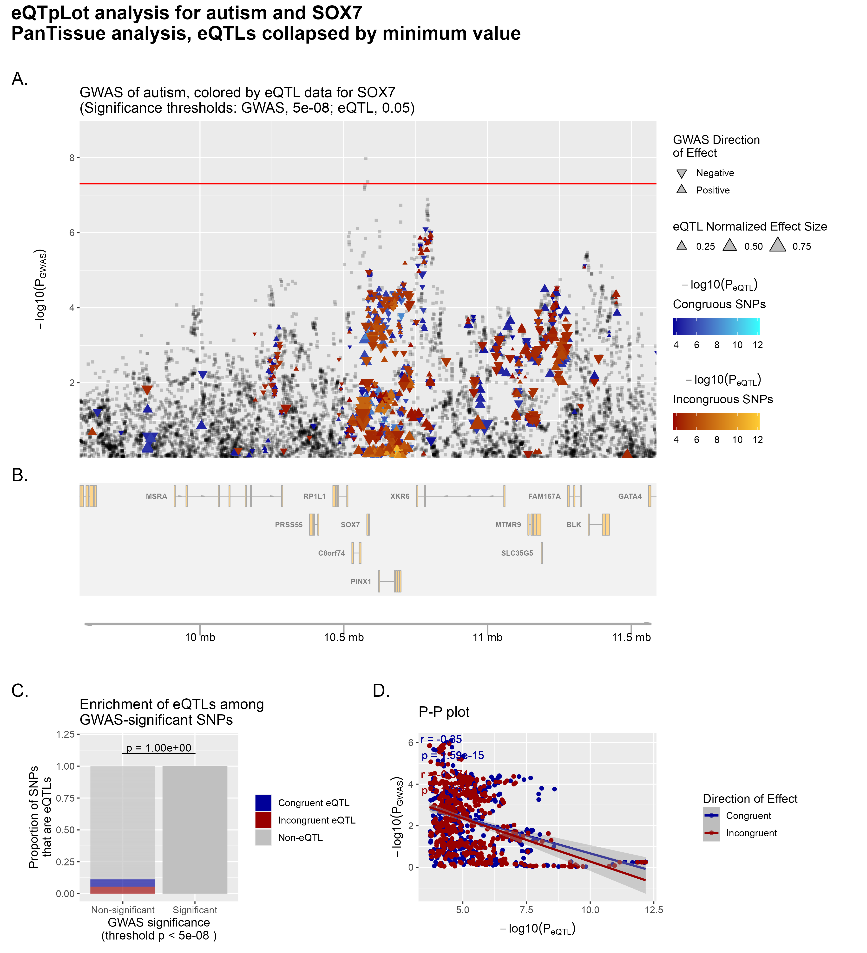
Supplementary Figure 23**:** eQTpLot for Pan Tissue analysis for *SOX7* in ASD. Linkage disequilibrium (LD) data is not included due to the fact that, after applying the supplied R2 and LD thresholds to filter the LD data, fewer than 2 SNPs that are also present in the GWAS summary statistics remain.


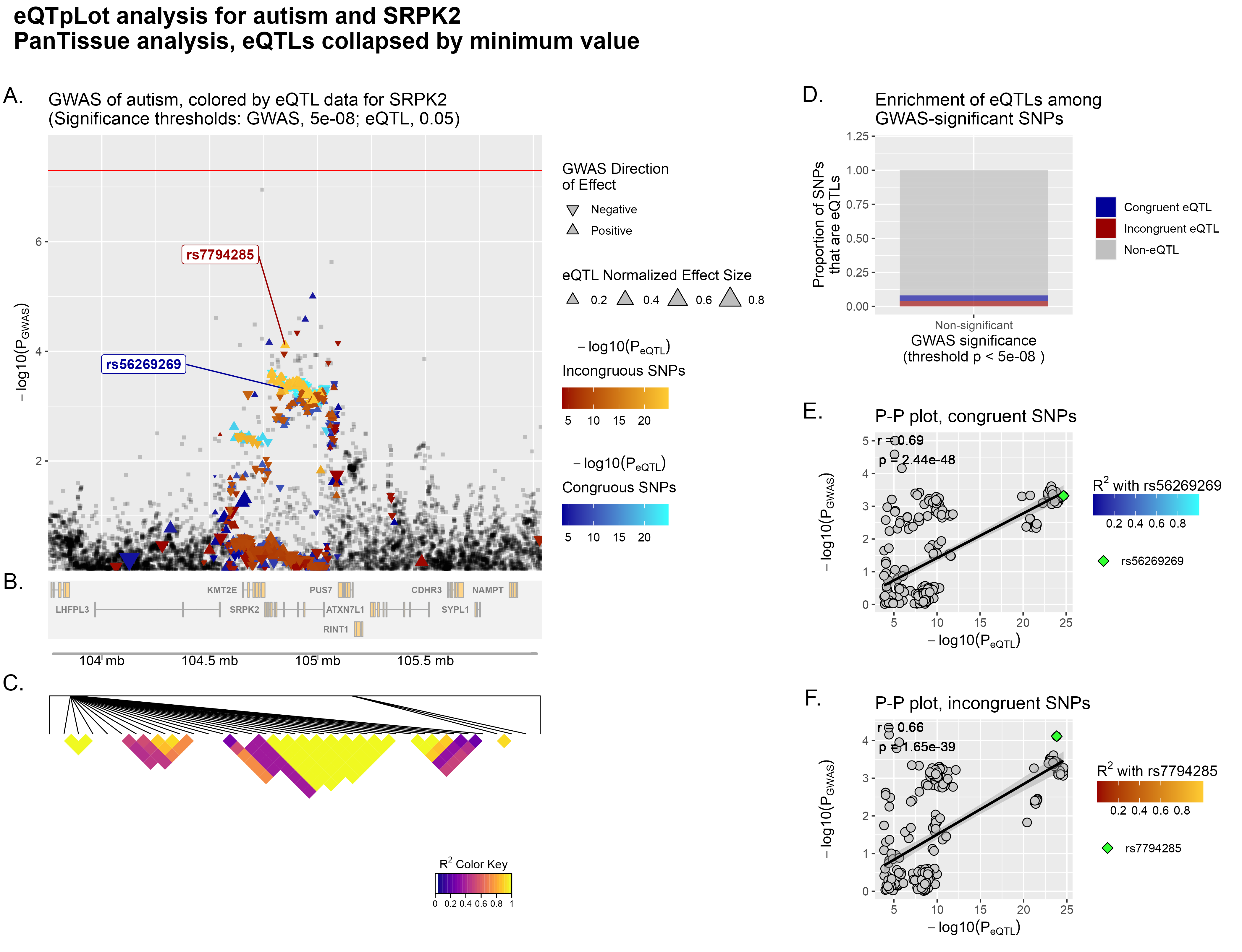
Supplementary Figure 24**:** eQTpLot for Pan Tissue analysis for *SRPK2* in ASD.


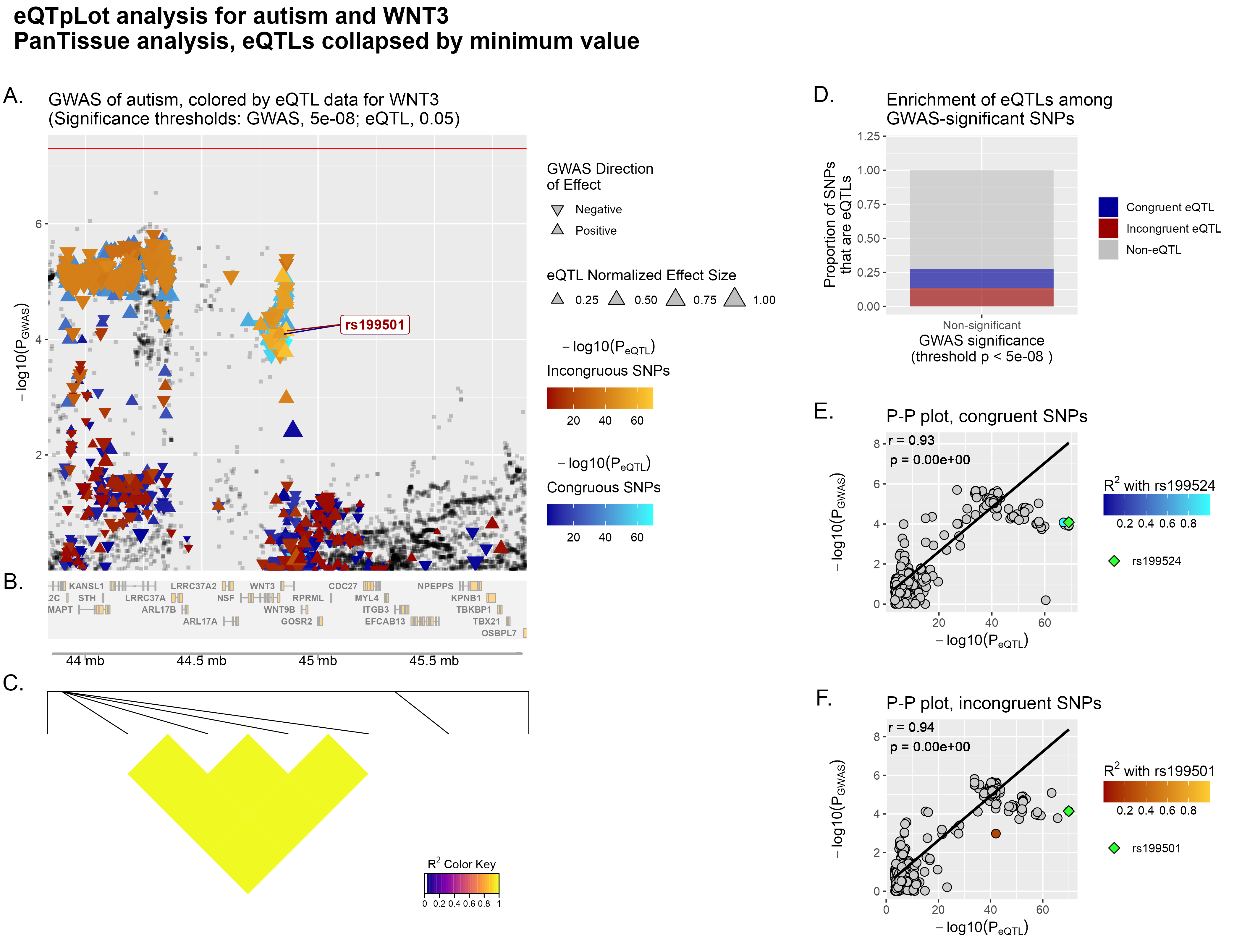
Supplementary Figure 25**:** eQTpLot for Pan Tissue analysis for *WNT3* in ASD.


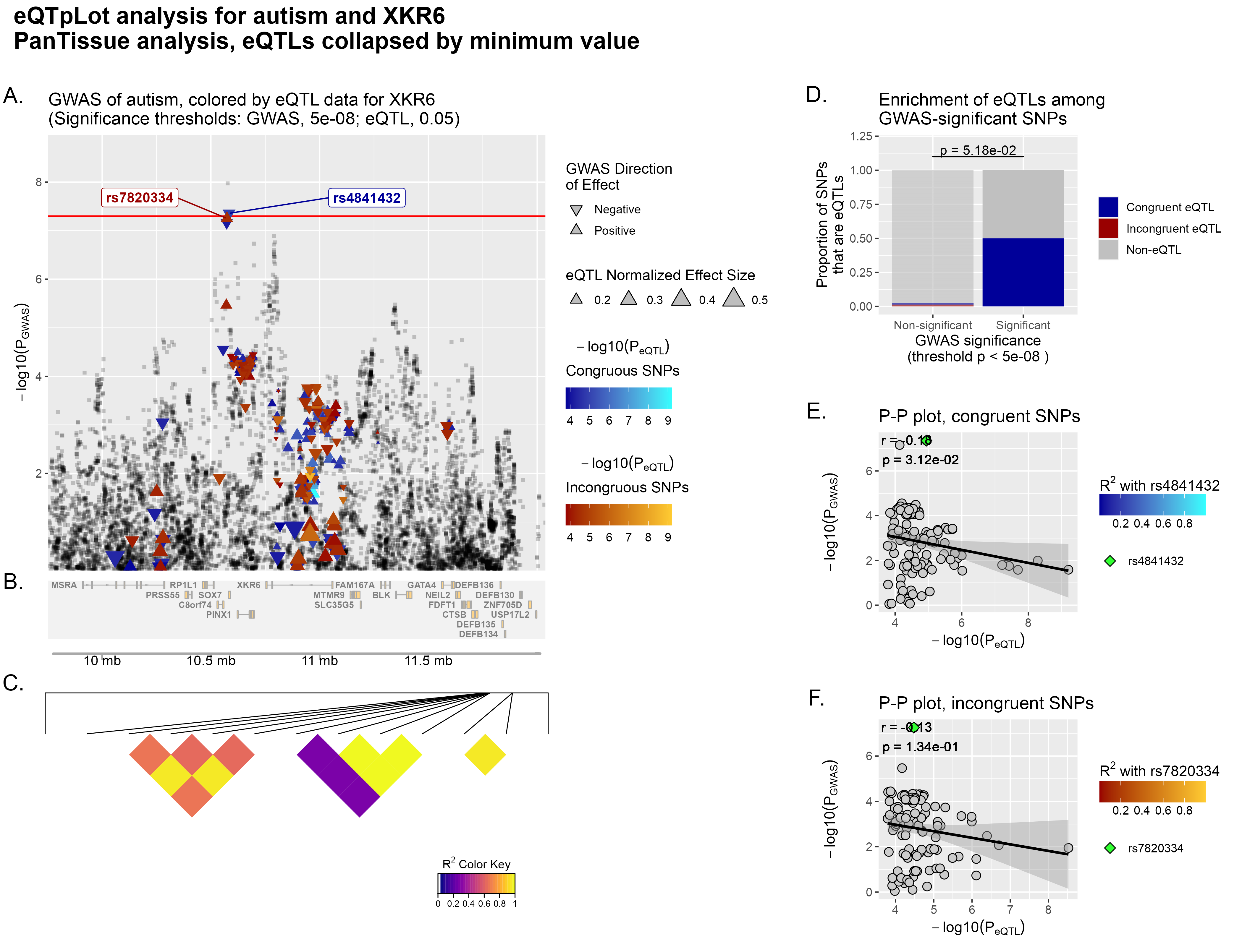


Supplementary Figure 26**:** eQTpLot for Pan Tissue analysis for *XKR6* in ASD.


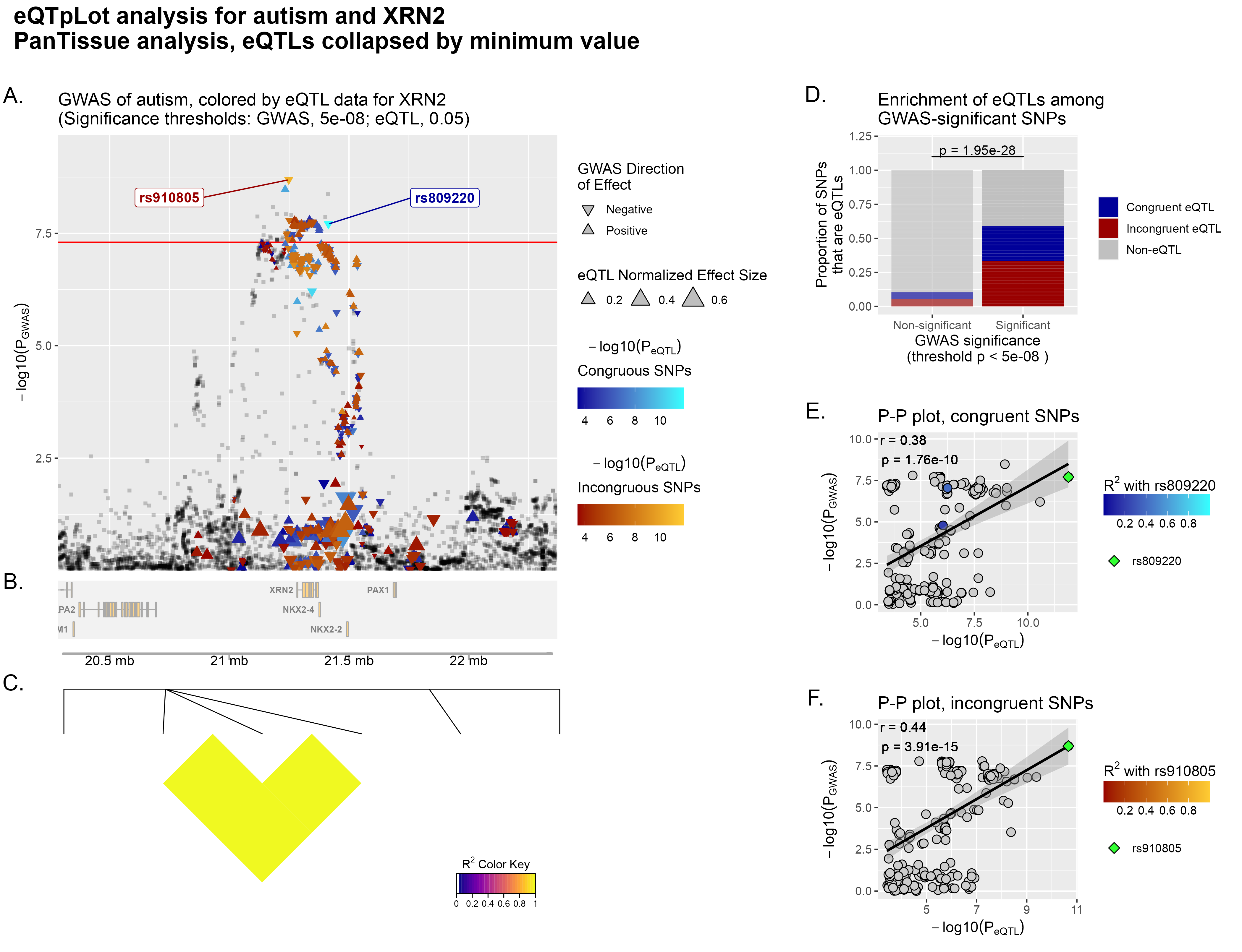


Supplementary Figure 27**:** eQTpLot for Pan Tissue analysis for *XRN2* in ASD.

##
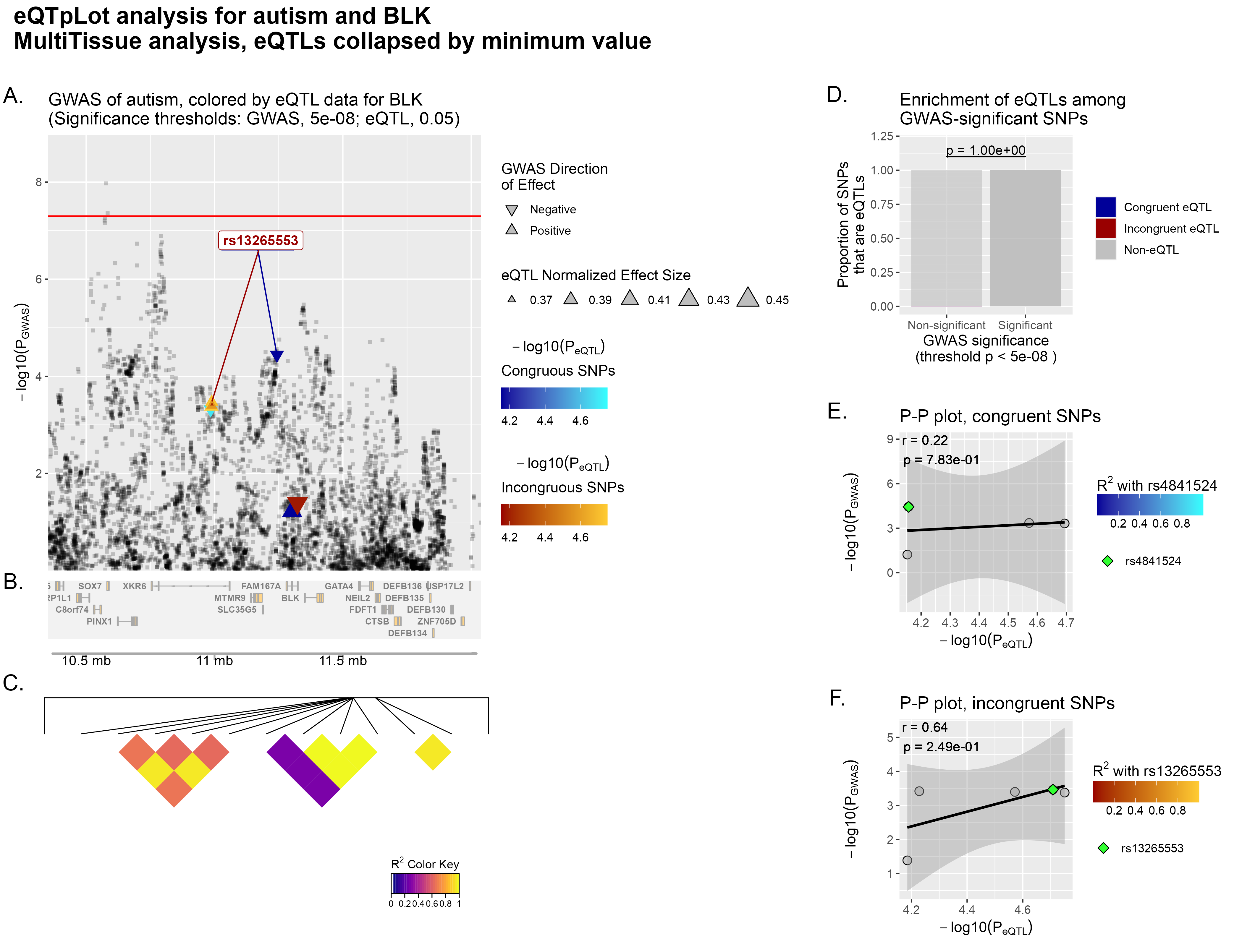
Multi Tissue Analysis

Supplementary Figure 28**:** eQTpLot for Multi Tissue analysis for *BLK* in ASD.


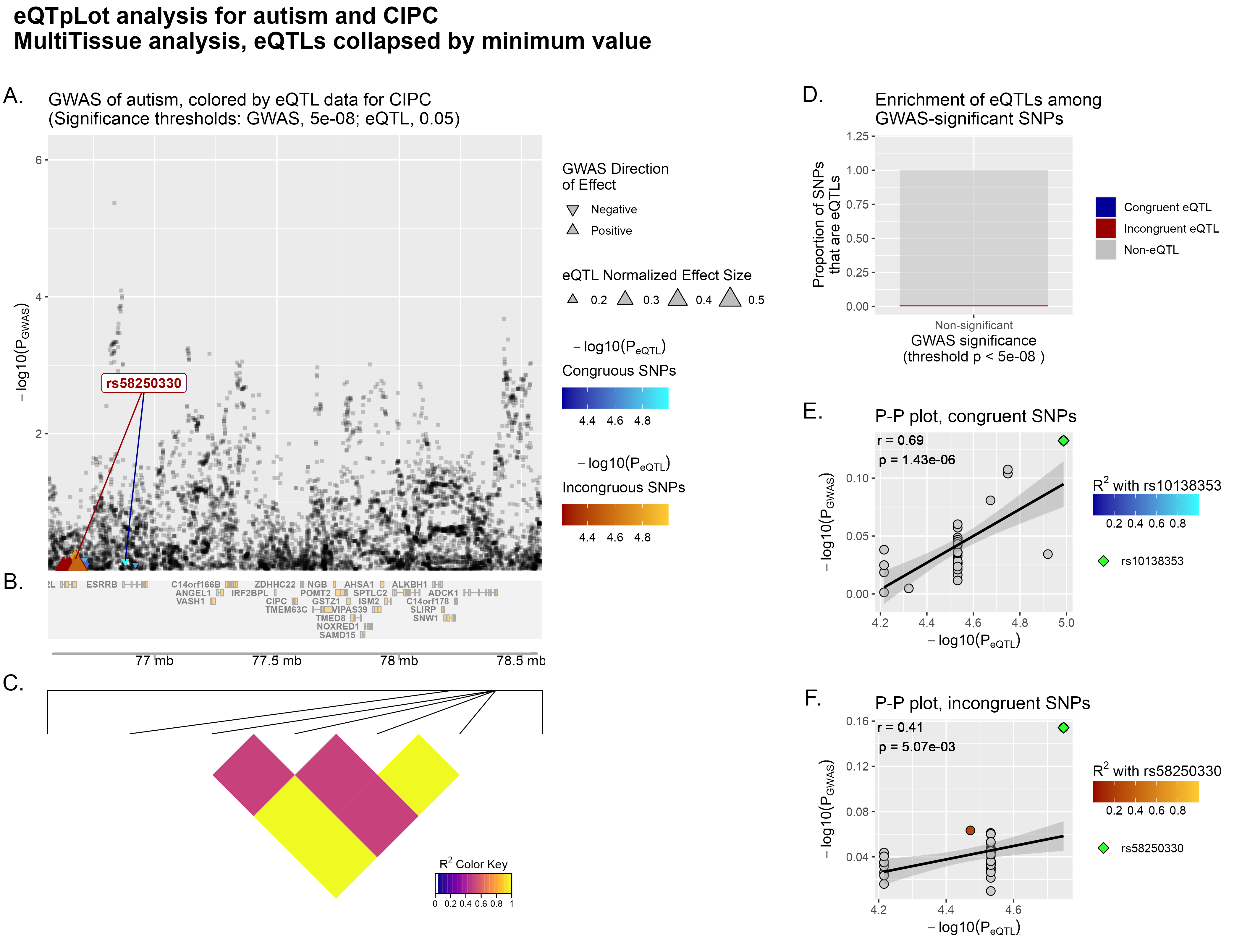


Supplementary Figure 29**:** eQTpLot for Multi Tissue analysis for *CIPC* in ASD.


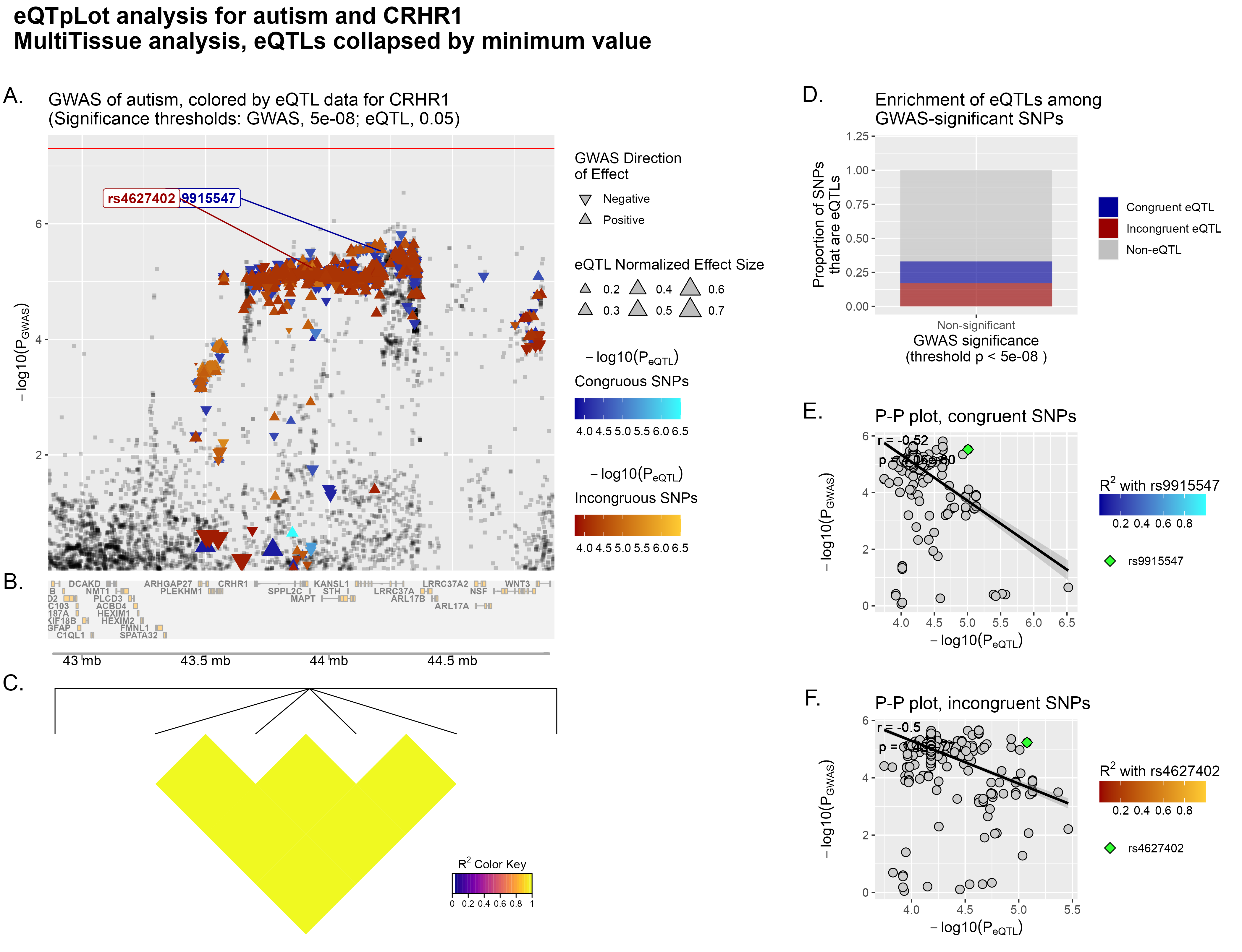
Supplementary Figure 30: eQTpLot for Multi Tissue analysis for *CRHR1* in ASD.


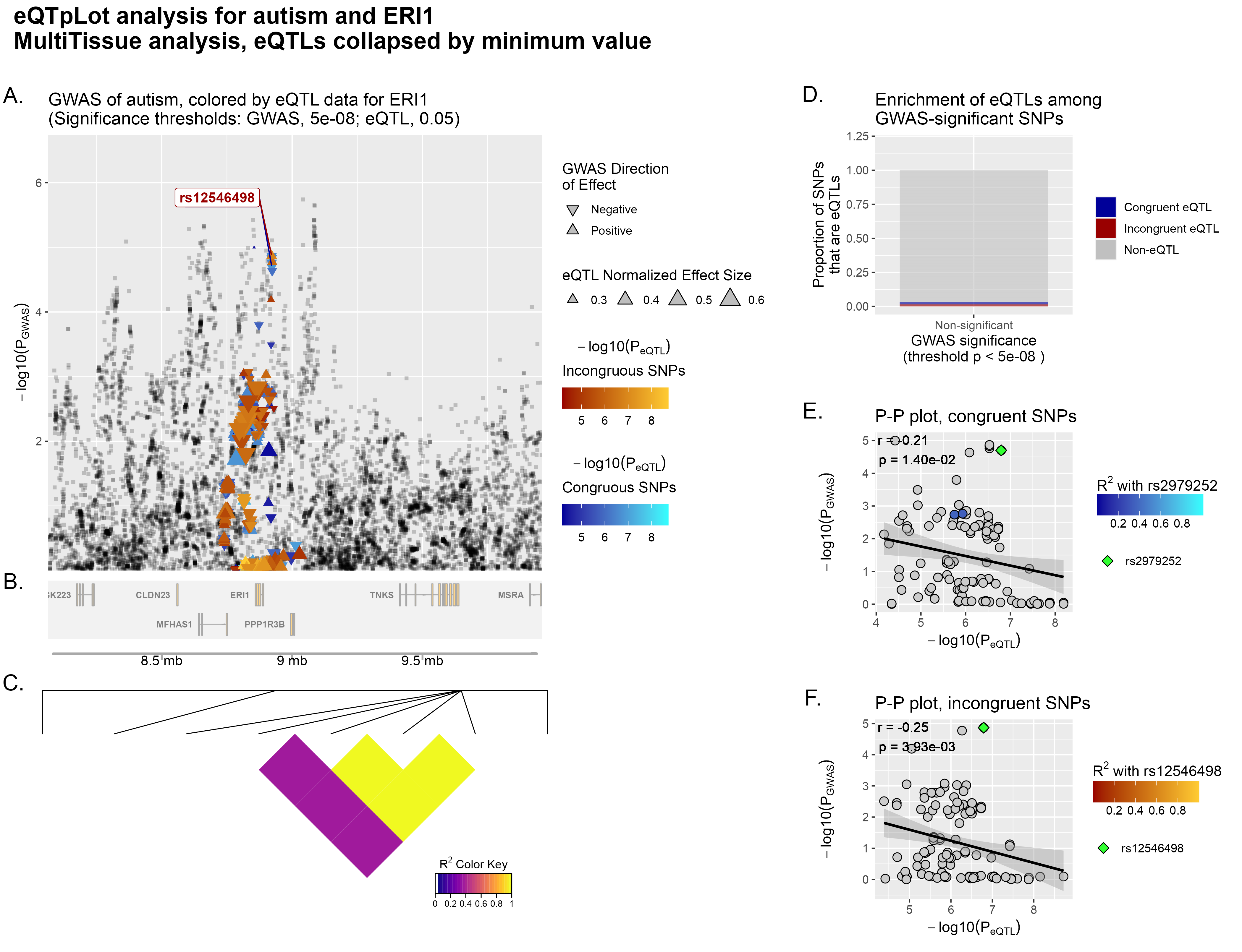


Supplementary Figure 31**:** eQTpLot for Multi Tissue analysis for *ERI1* in ASD.


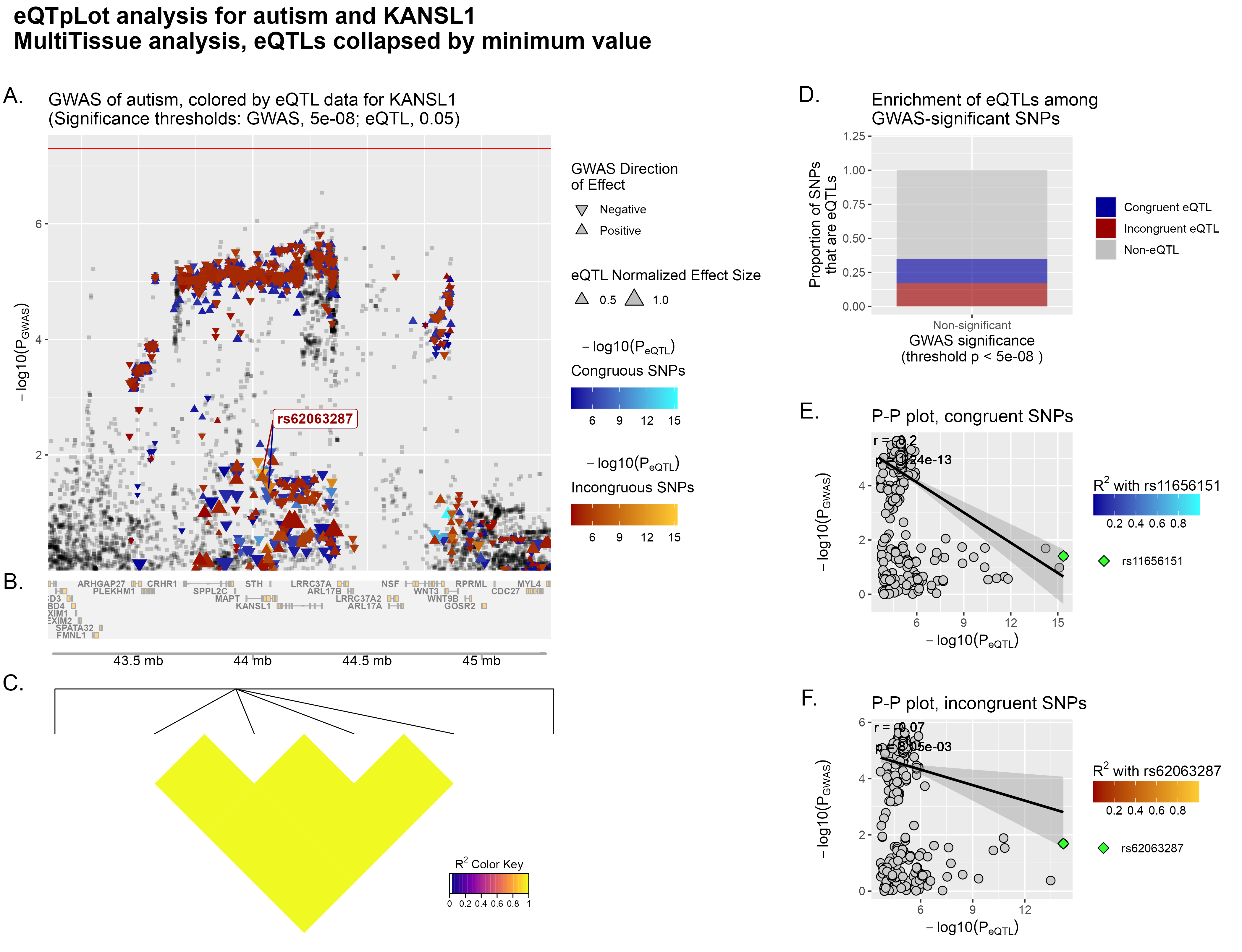
Supplementary Figure 32**:** eQTpLot for Multi Tissue analysis for *KANSL1* in ASD.


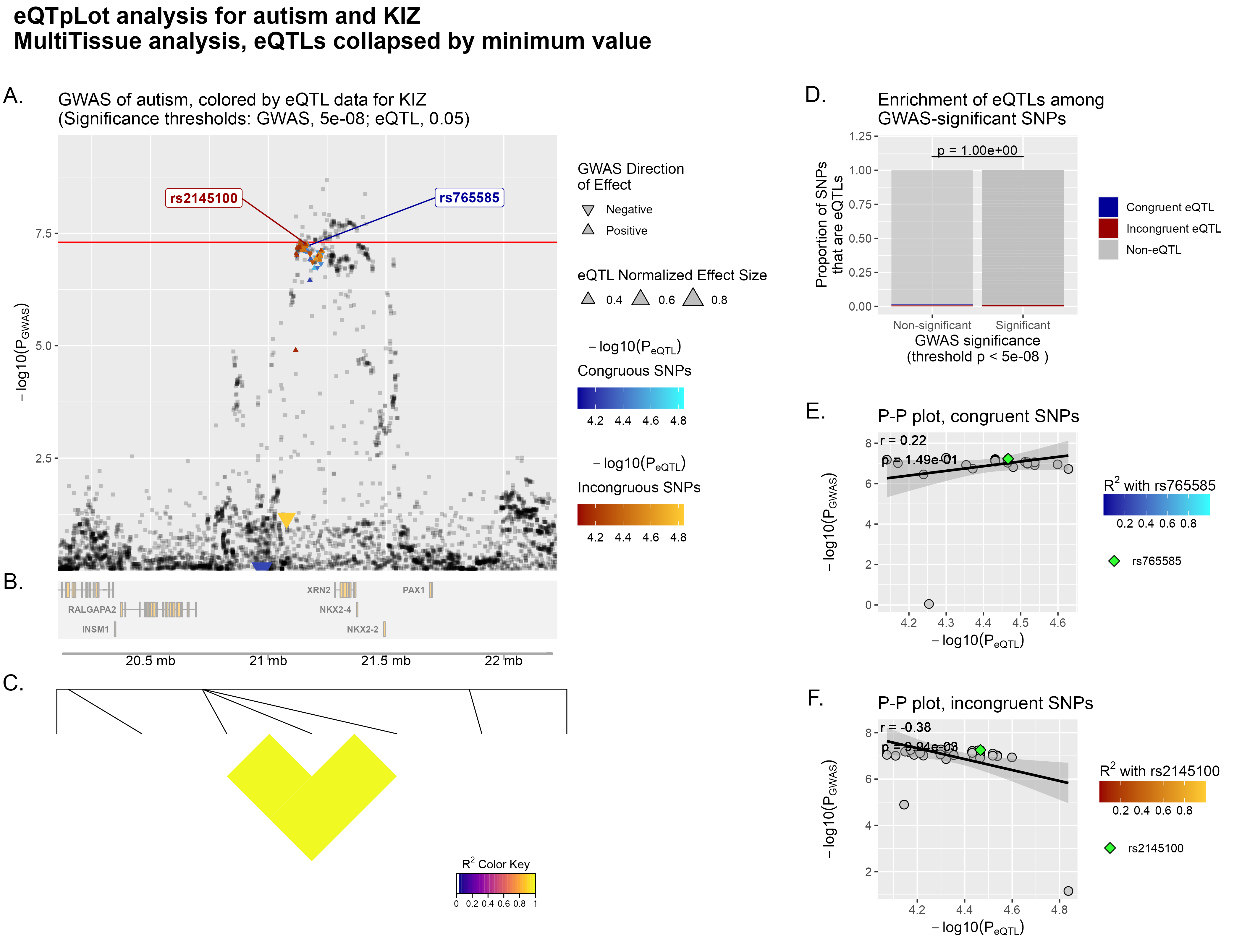


Supplementary Figure 33**:** eQTpLot for Multi Tissue analysis for *KIZ* in ASD.


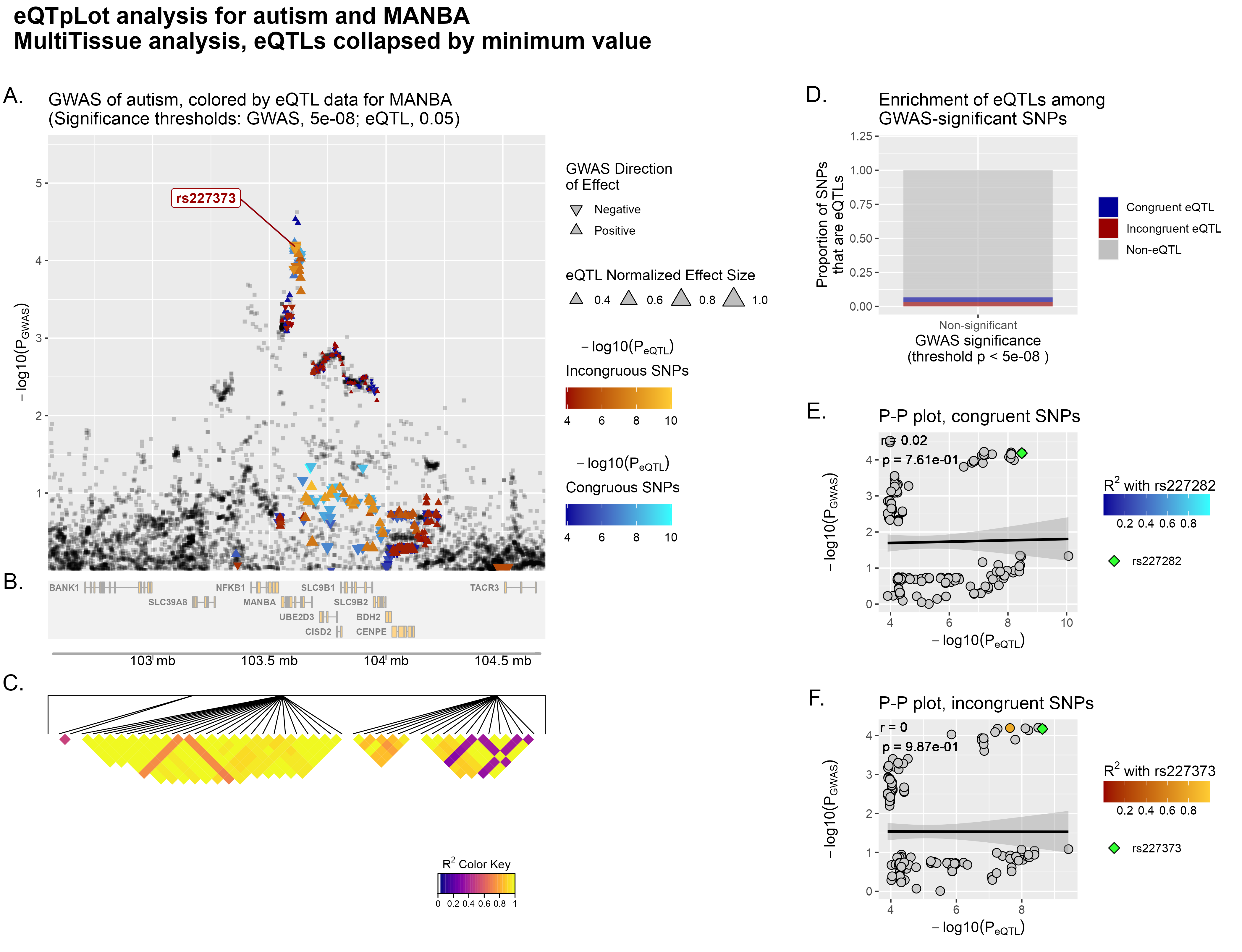
Supplementary Figure 34**:** eQTpLot for Multi Tissue analysis for *MANBA* in ASD.


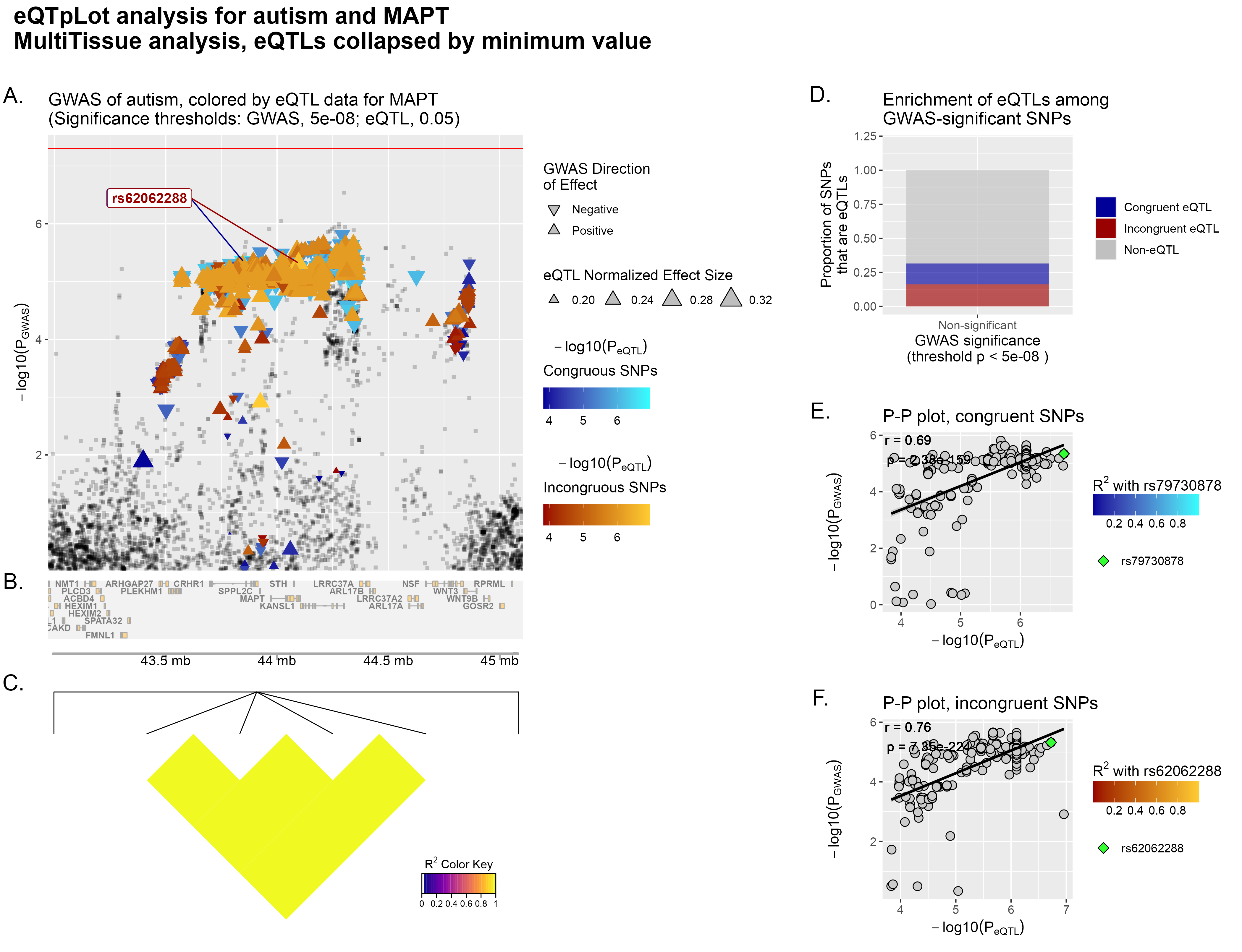


Supplementary Figure 35**:** eQTpLot for Multi Tissue analysis for *MAPT* in ASD.


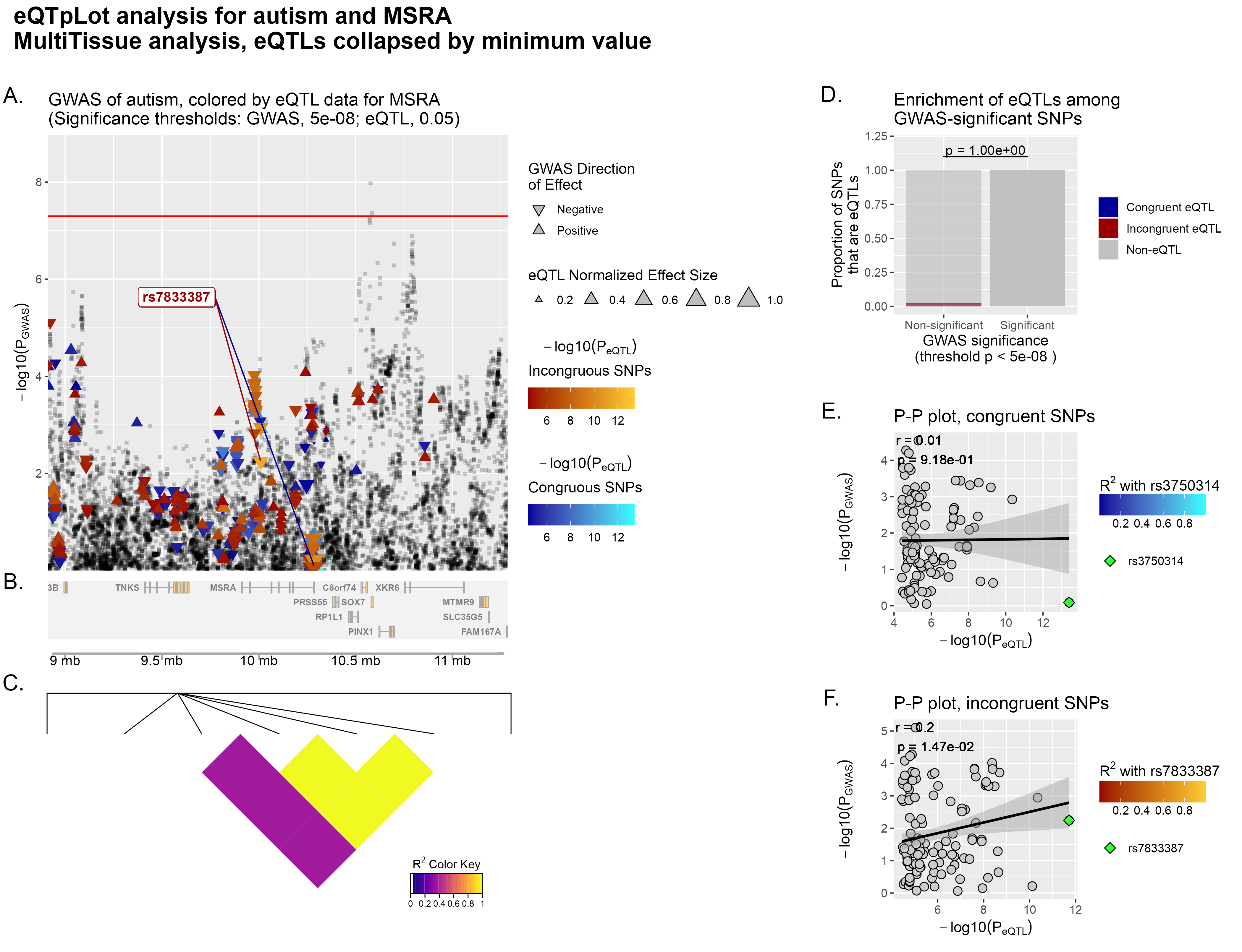


Supplementary Figure 36**:** eQTpLot for Multi Tissue analysis for *MSRA* in ASD.


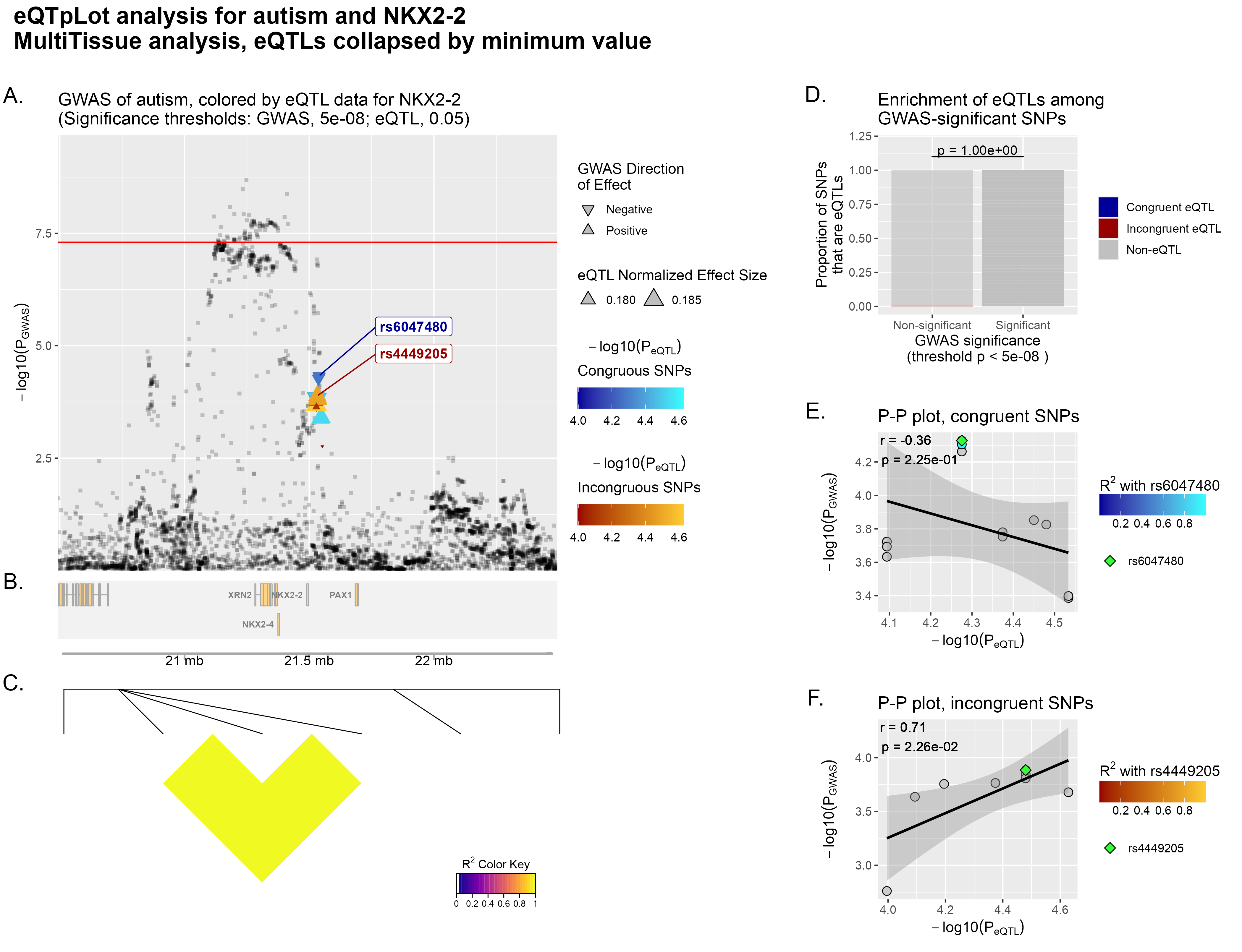


Supplementary Figure 37**:** eQTpLot for Multi Tissue analysis for *NKX2-2* in ASD.


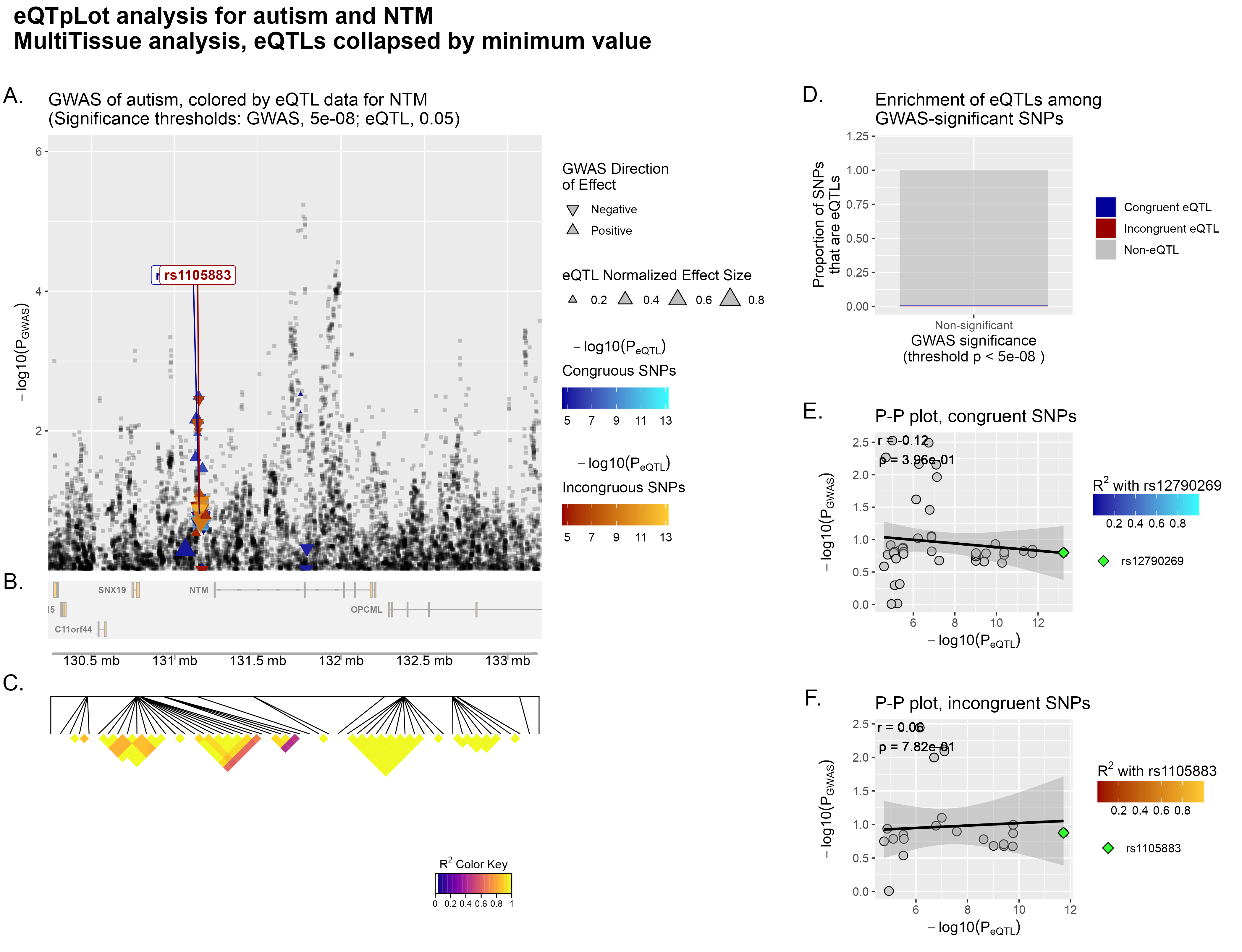
Supplementary Figure 38**:** eQTpLot for Multi Tissue analysis for *NTM* in ASD.

**
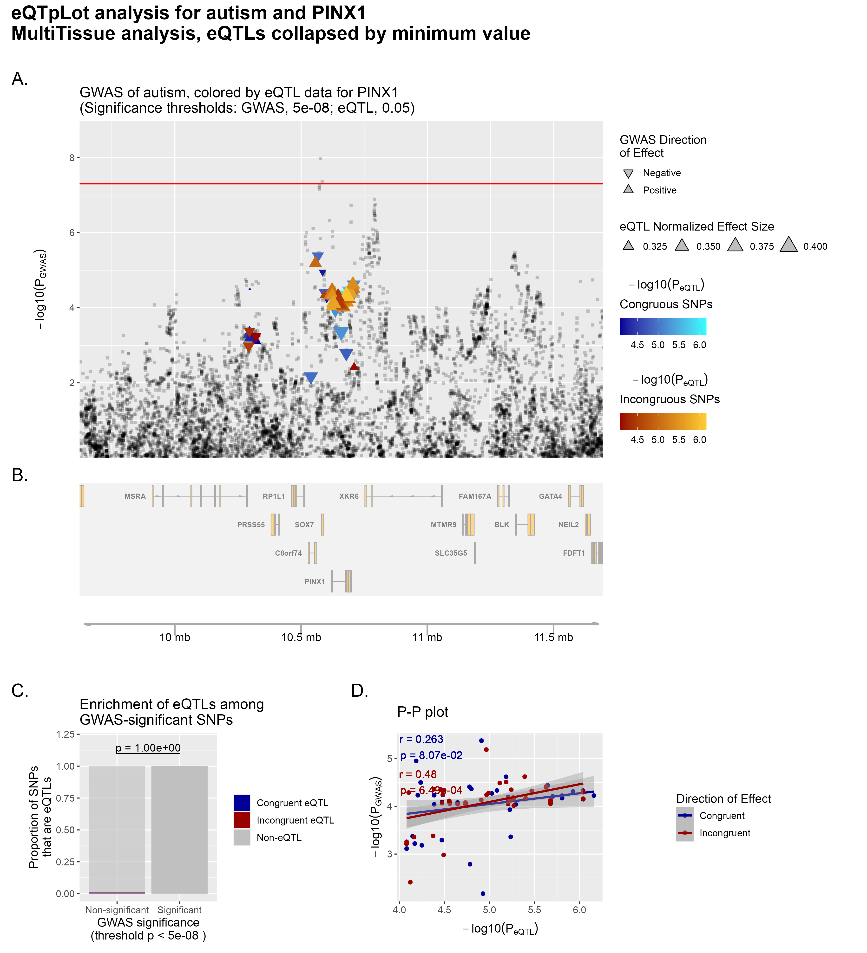
**

Supplementary Figure 39**:** eQTpLot for Multi Tissue analysis for *PINX1* in ASD. Linkage disequilibrium (LD) data is not included due to the fact that, after applying the supplied R2 and LD thresholds to filter the LD data, fewer than 2 SNPs that are also present in the GWAS summary statistics remain.


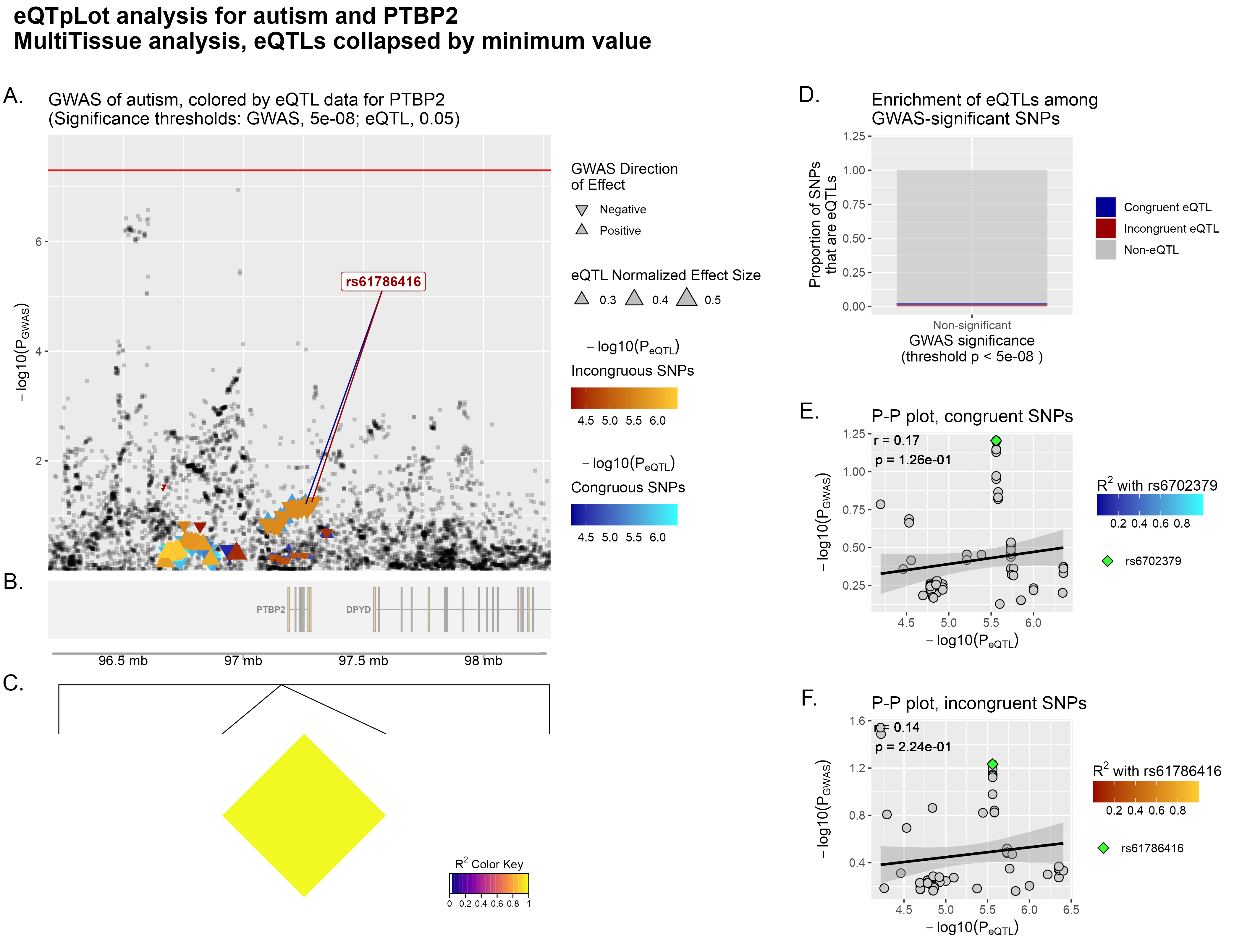


**S**upplementary Figure 40**:** eQTpLot for Multi Tissue analysis for *PTBP2* in ASD.


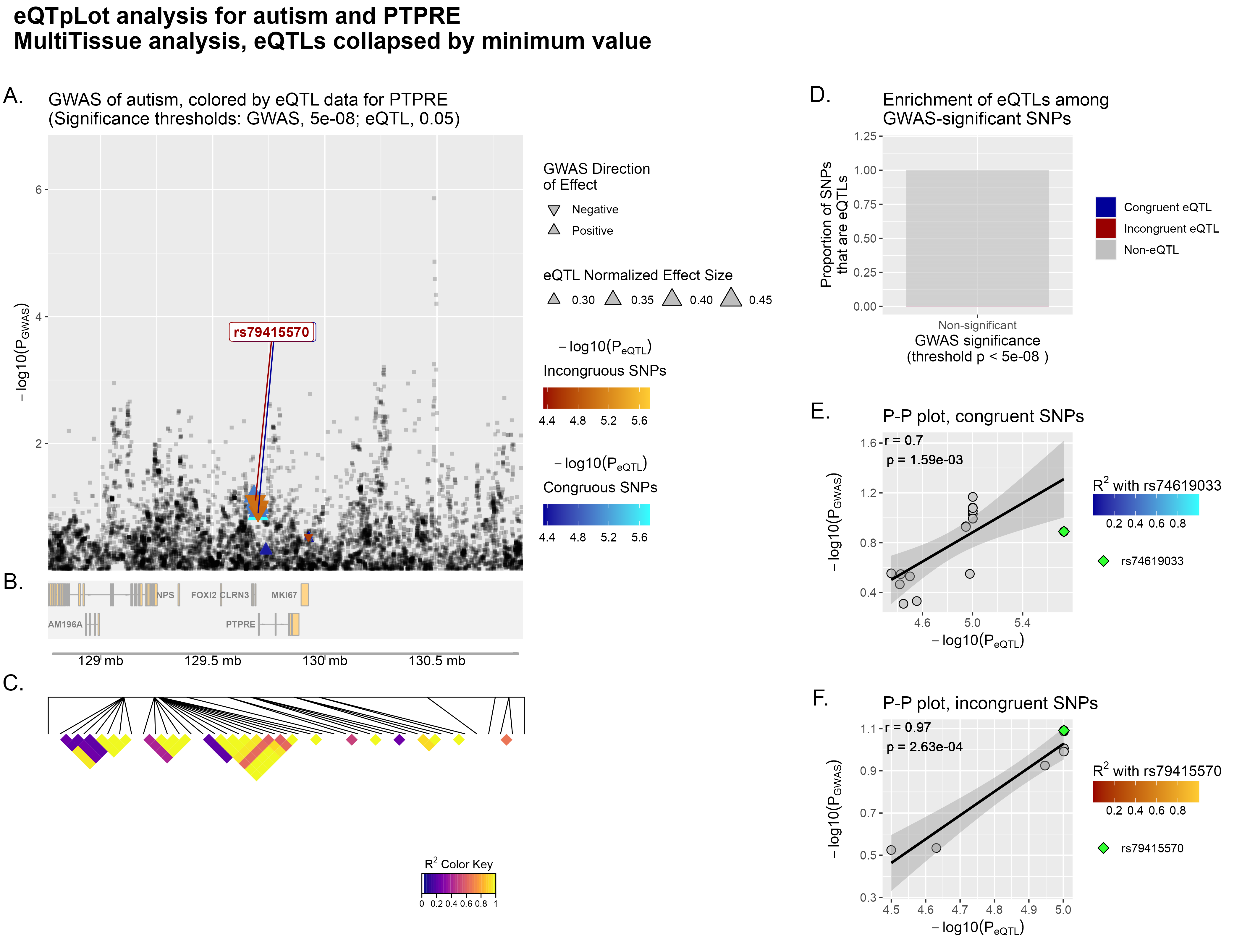


Supplementary Figure 41**:** eQTpLot for Multi Tissue analysis for *PTPRE* in ASD.


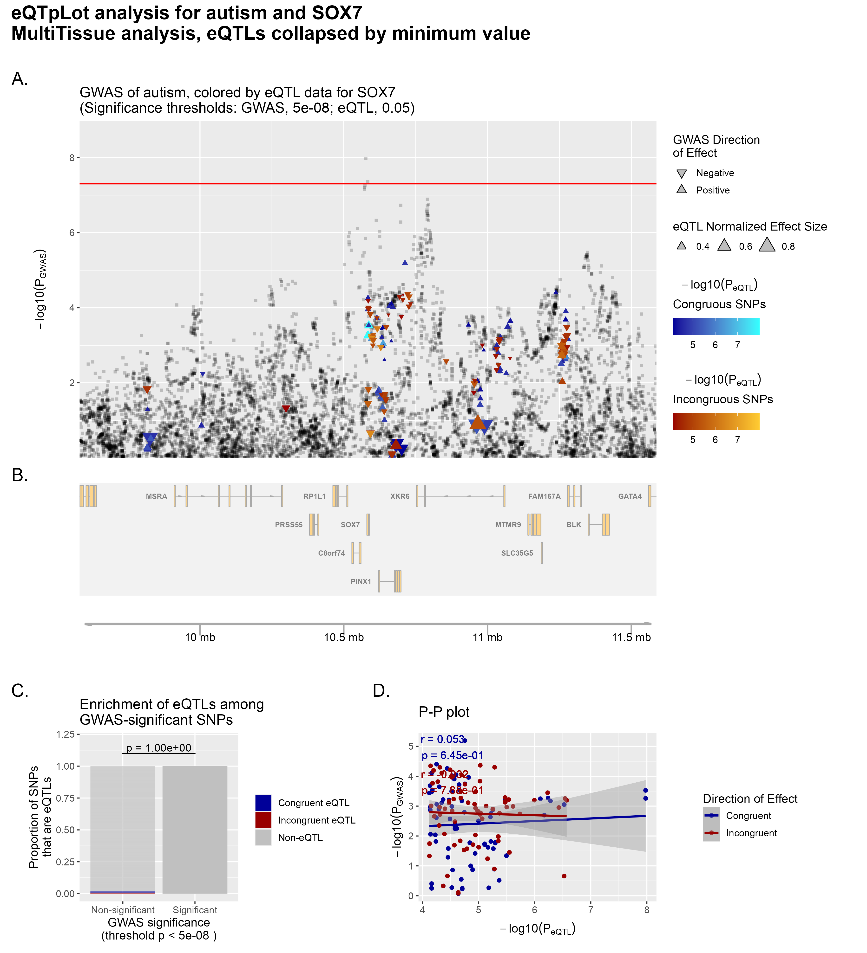


Supplementary Figure 42**:** eQTpLot for Multi Tissue analysis for *SOX7* in ASD. Linkage disequilibrium (LD) data is not included due to the fact that, after applying the supplied R2 and LD thresholds to filter the LD data, fewer than 2 SNPs that are also present in the GWAS summary statistics remain.


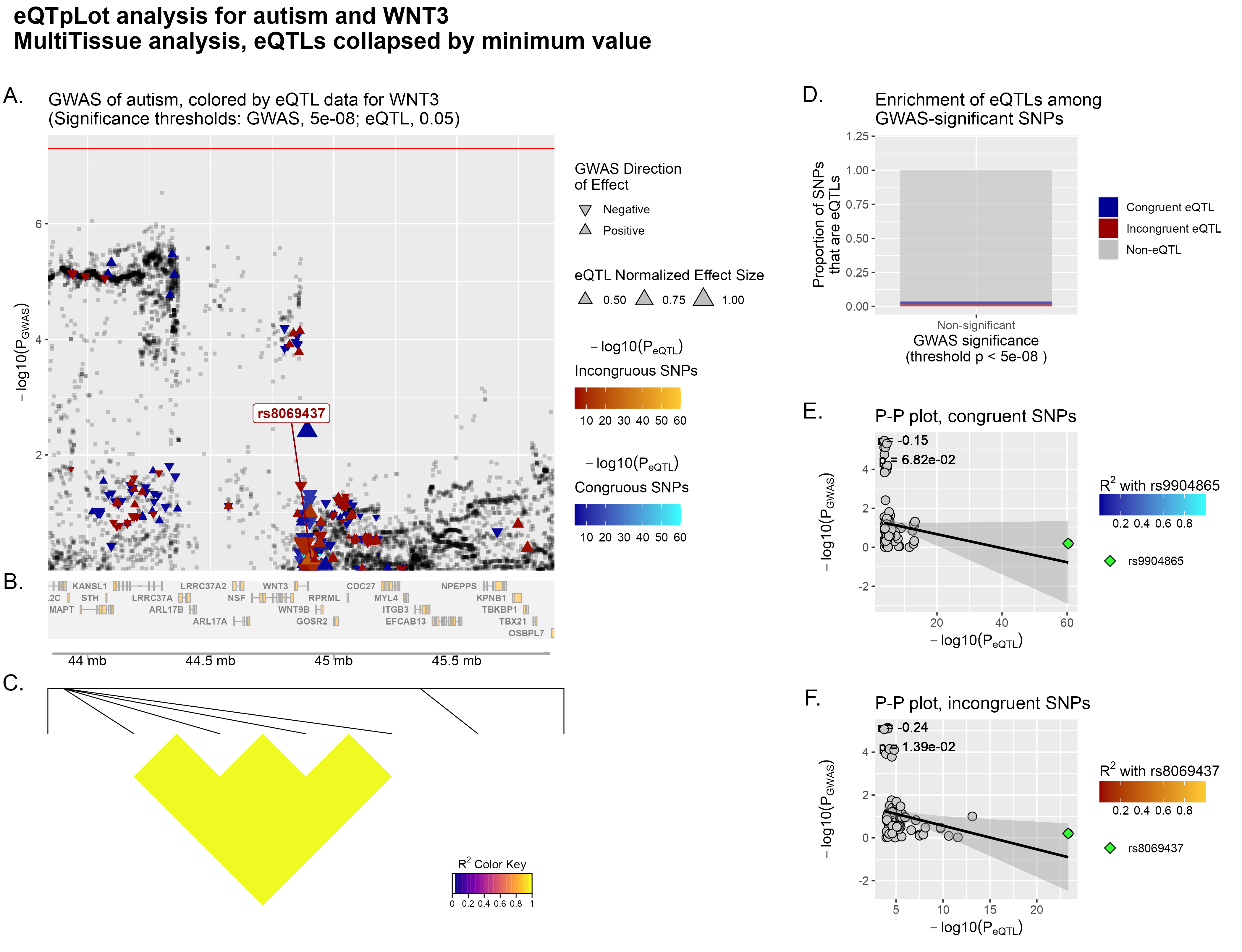


Supplementary Figure 43**:** eQTpLot for Multi Tissue analysis for *WNT3* in ASD.


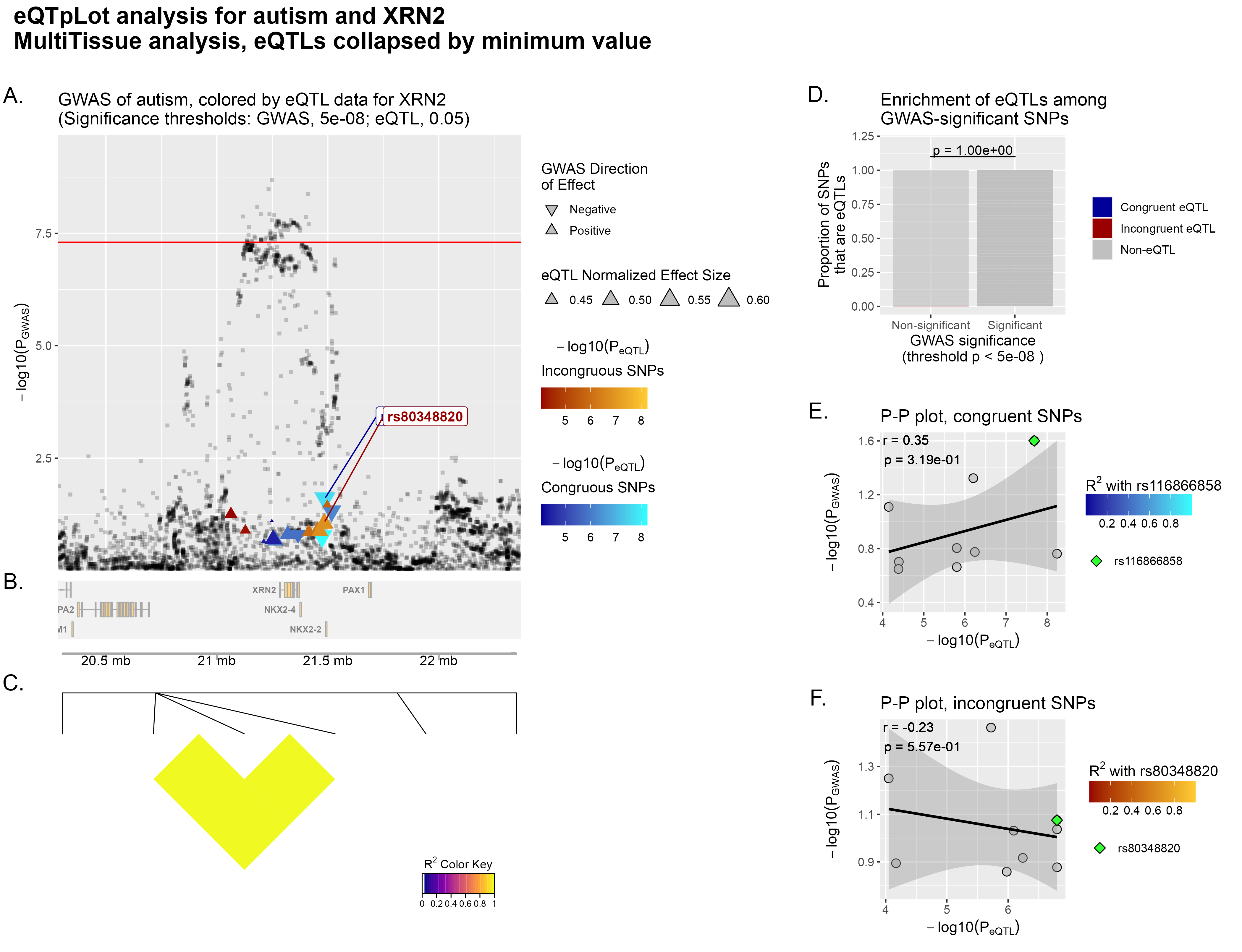


Supplementary Figure 44**:** eQTpLot for Multi Tissue analysis for *XRN2* in ASD.

# BIBLIOGRAPHY

1. Pardiñas AF, Holmans P, Pocklington AJ, et al. Common schizophrenia alleles are enriched in mutation-intolerant genes and in regions under strong background selection. *Nat Genet*. 2018;50(3):381-389. doi:10.1038/s41588-018-0059-2

2. Schizophrenia Working Group of the Psychiatric Genomics Consortium. Biological insights from 108 schizophrenia-associated genetic loci. *Nature*. 2014;511(7510):421-427. doi:10.1038/nature13595

3. Demontis D, Walters RK, Martin J, et al. Discovery of the first genome-wide significant risk loci for attention deficit/hyperactivity disorder. *Nat Genet*. 2019;51(1):63-75. doi:10.1038/s41588-018-0269-7

4. Howard DM, Adams MJ, Clarke TK, et al. Genome-wide meta-analysis of depression identifies 102 independent variants and highlights the importance of the prefrontal brain regions. Published online 2019:33.

5. Boughton AP, Welch RP, Flickinger M, et al. LocusZoom.js: interactive and embeddable visualization of genetic association study results. *Bioinformatics*. 2021;37(18):3017-3018. doi:10.1093/bioinformatics/btab186
